# Comprehensive review on COVID-19: etiology, pathogenicity, and treatment

**DOI:** 10.3389/fmed.2025.1569013

**Published:** 2025-09-23

**Authors:** Amr El Zawily, Sarah Eckert, Reed Adajar, Nardin Wagih, Abeer H. Elmaidomy, Abdelrahman M. Helmy, Mazen Mustafa, Mazhar Elshorbagi, Erin Ghali, Rehab G. Fadl, Jochen Bodem, Usama Ramadan Abdelmohsen, Marco Y. W. Zaki

**Affiliations:** ^1^University of Guelph, Department of Molecular and Cellular Biology, Guelph, ON, Canada; ^2^Department of Biology, University of Iowa, Iowa City, IA, United States; ^3^Department of Medicinal Chemistry, Faculty of Pharmacy, Deraya University, Minia, Egypt; ^4^Department of Pharmacognosy, Faculty of Pharmacy, Beni-Suef University, Beni-Suef, Egypt; ^5^Department of Pharmaceutics and Pharmaceutical Technology, Faculty of Pharmacy, Badr University in Assiut, Assiut, Egypt; ^6^Deraya Center for Scientific Research, Deraya University, Minia, Egypt; ^7^Department of Philosophy, Deraya University, Minia, Egypt; ^8^Department of Biochemistry, Faculty of Pharmacy, Minia University, Minia, Egypt; ^9^Institute of Virology and Immunobiology, University of Würzburg, Würzburg, Germany; ^10^Department of Pharmacognosy, Faculty of Pharmacy, Minia University, Minia, Egypt; ^11^Centre for Research and Sustainability, Deraya University, Minia, Egypt

**Keywords:** COVID-19, etiology, pathogenicity, pathophysiology, synthetic, natural products

## Abstract

With the unprecedented surge of severe COVID-19 cases in early 2020, researchers and medical professionals worked actively to identify effective viral infection treatments based on a scientific understanding of viruses. Over the past few years, an enormous amount of research has investigated the viral infection and replication processes following the first SARS-CoV-2 case. With this knowledge, many drugs have been explicitly created to inhibit viral replication or decrease the severity of the immune response. Additionally, scientists have utilized decades of research and techniques to expedite SARS-CoV-2 vaccine development. SARS-CoV-2, a positive-strand RNA virus, belongs to the Sarbecovirus subgroup of Betacoronaviruses. Its emergence is not unique; previous outbreaks like SARS and MERS have shaped our understanding of coronavirus-related diseases. Molecular clock analysis suggests that the ancestor of all current coronaviruses existed over 10,000 years ago, with subsequent evolution occurring around 3300–2400 BC. Researchers have explored synthetic and natural treatments alongside other antiviral therapies, corticosteroids, and immunotherapies. Additionally, using artificial intelligence and nano-based technologies enriched SARS-CoV-2 diagnosis and management. In this comprehensive review, we provide recent literature on COVID-19, exploring its evolving etiology, pathogenicity, and pathophysiology, alongside developments in synthetic and natural therapeutic strategies, vaccines, artificial intelligence in diagnosis, and nano-based technologies.

## Introduction

Coronavirus is a positive-strand RNA virus that infects many mammalian species, causing respiratory tract-related diseases of varying severity (1). The SARS-CoV-2 virus that caused the latest outbreak is a member of the Sarbecovirus subgroup of the Betacoronavirus (1). The evolution of the severe acute respiratory syndrome (SARS) caused by SARS-CoV-1 in 2003 and the Middle East Respiratory Syndrome (MERS) caused by MERS-CoV in 2012 preceded the outburst of the current SARS-CoV-2 pandemic (2). Other milder or sub-clinical coronavirus-associated diseases further support the idea that SARS-CoV-2 was not extraordinary and may reappear in the future (3). Molecular clock analysis revealed that the ancestor of all the current groups of coronaviruses existed more than 10,000 years ago, with different groups of coronaviruses evolving around 3300–2400 BC (1). The rapid SARS-CoV-2 transmission and the uncertainty of its related effects rushed the introduction of new treatment regimens to contain the pandemic. The review outlines the virus's etiology, pathogenicity, and pathophysiology. It details various synthetic and natural therapies exploring their efficacy in preventing or treating COVID-19 based on the available *in-silico, in-vitro, in-vivo*, and clinical data (Table 1). Finally, different types of SARS-CoV-2 vaccines, their effectiveness, safety, and potential future improvements, including artificial intelligence advancements and nano-based technologies, are thoroughly addressed.

**Table 1:** Comparison of COVID-19 vaccine platforms: mechanisms, advantages, limitations, and clinical status.

**Vaccine platform**	**Advantages**	**Limitations**	**Example/s**	**Developers**	**Phase and clinical trial registration number**
mRNA	- Rapid development - High efficacy (248)	-Require ultra-cold storage (248)	Comirnaty (BNT162b2)	Pfizer/BioNTech	Phase 3/4 (NCT04368728; NCT04760132)
			mRNA-1273 or Spikevax	Moderna	Phase 3/4 (NCT04470427; NCT04760132)
Non-replicating Viral Vector	-Strong cellular and humoral immune responses without the need for adjuvants (248)	Pre-existing immunity against vector may reduce efficacy (248)	Ad26.COV2–S (Adenovirus)	Johnson & Johnson's	Phase 4 (NCT04505722; NCT05075538)
Inactivated Virus	- Traditional method - Broad antigenic profile - Easy storage compared to mRNA vaccines (248)	- Variable efficacy - Weak response necessitates the use of booster doses (248)	BBIBP-CorV by Sinopharm	Sinopharm	Phase 4 (NCT04560881; NCT04863638)
			CoronaVac (PiCoVacc)	Sinovac Research and Development Co., Ltd.	Phase 4 (NCT04456595; NCT04756830; NCT04747821)
DNA	- Stable at higher temperatures compared to mRNA vaccines - Activate both humoral and cellular immune responses (248)	-Potential safety concerns with genetic integration and toxicity (248)	ZyCoV-D	Zydus Cadila	Phase 3 (CTRI/2022/06/043365)
Protein Subunit	- Targets key antigens - Safe for immunocompromised individuals/Non-infectious (248)	-May need adjuvants or repeat doses to boost response (248)	NVX-CoV2373 (Covovax or Nuvaxovid)	Novavax	Phase 3 (NCT04611802)
VLP	- Safe/non-infectious - Strong immune response (248)	-Complex manufacturing process compared to protein subunit vaccines (248)	COVID-19 (CoVLP)	Medicago Inc.	Phase 3 (NCT05040789)

## Evolution of SARS-CoV-2

### The origin of SARS-CoV-2

Most coronavirus strains are animal-derived. However, host coinfection with different variants of SARS-related coronaviruses can give rise to viral recombination, which is one hypothesis of how SARS-CoV-2 has evolved (4). The phylogenetic relativity between the SARS-CoV-2 and Yunnan's RaTG13 bat virus is noteworthy; the sequence similarity between both strains is around 96% (5). However, synonymous substitutions, which do not alter the amino acid sequence—between both strains reduced that similarity to only 83% (6). The error rate in coronavirus replication is significantly lower than that of influenza due to the viral proofreading exonuclease nsp14 (7, 8), and the synonymous evolutionary rate of SARS-CoV-2 ranges between 1.19–1.31 × 10^−3^/site/year (9–13). This leads to a divergence time between SARS-CoV-2 and RaTG13, between 18 and 71.4 years (12, 13). The advanced Bayesian phylogenetic method hypothesized that SARS-CoV-2 and RaTG13 did exist with their Most Recent Common Ancestor (MRCA) in 1969 (14), suggesting that both SARS-CoV-2 and RaTG13 might be divergent and pointing to the need to find other coronaviruses that are more similar to SARS-CoV-2 than RaTG13. The alignment of 68 different Sarbecovirus strains revealed multiple “recombination blocks” between recombination breakpoints in the SARS-CoV-2 genome, especially in areas translated to ORF1 and the S protein (14). Specifically, the ORF1b, the 5′ region of the S protein, and the nucleocapsid protein regions in both SARS-CoV-2 and RaTG13 showed more similarity than other coronavirus species (14). Interestingly, in these genomic regions, the 2019 Pangolin Guangdong coronavirus (15) showed a solid relationship to the ancestor of both SARS-CoV-2 and RaTG13 (14, 15). Strikingly, the SARS-CoV-2 variable loop region of the S protein, which contains six residues in the Angiotensin Converting Enzyme 2 (ACE-2) Receptor Binding Domain (RBD), had more substantial similarity to the sequence of Pangolin Guangdong coronavirus compared to that of RaTG13 (15). This either suggests a potential recombination between SARS-CoV-2 and the Pangolin Guangdong strain after the split of SARS-CoV-2 and RaTG13 lineages or a recombination between Pangolin Guangdong and the common ancestral strain of both SARS-CoV-2 and RaTG13 with the later gaining more sequence changes from the SARS-CoV-2 strain (14, 15). Phylogenetic analysis of the S protein also showed higher mutability for deletions, mutations, and recombination (16) in line with other studies that confirmed the divergence of the RBD region in the S protein of SARS-CoV-2 from the RaTG13 (17). Although it is hard to determine whether the evolution of SARS-CoV-2 occurred via direct transmission or an intermediate host, the evolution of the SARS-CoV-2 genome witnessed a series of recombination events from other viruses. Altogether, there are different tiers of complexity in the structure and evolution of the S protein, among other parts of the SARS-CoV-2 genome. Further investigation into the functional relevance of these events to the infection and progression process is warranted.

### SARS-CoV-2 variants

The evolution of new SARS-CoV-2 variants can be attributed to point mutations, recombination between SARS-CoV-2 variants, and host-mediated RNA editing through the Apolipoprotein B mRNA Editing enzyme, Catalytic polypeptide (APOBEC) and Adenosine Deaminase Acting on RNA (ADAR) enzymes (14). Emergent SARS-CoV-2 variants are hard to trace and classify due to low nucleotide substitutions and the lack of appropriate sequencing techniques in some countries witnessing their outbreak (18). The substitution of one amino acid residue (D614G) in the spike protein from the Wuhan-Hu-1 sequence (NC_045512.2) became 100% prevalent by June 2020. It was the first evidence of the SARS-CoV-2 evolutionary selective pressure (19). At the end of 2020, the UK reported that infection of the new Alpha (B.1.1.7) variant was first reported in September of the same year. This fast-rising lineage was associated with a higher rate of mutagenesis of its spike proteins. A few weeks later, South Africa reported the evolution of the Beta (B.1.351) variant that regionally predominated in just 2 months (10). In Brazil, the Gamma (P.1) variant occurred with a prevalence of 75% by October 2020 (20). In 2021, another variant, Delta (B.1.617.2), was reported in India and spread to many other countries (21). The transmission rate and the severity of the B.1.617.2 variant surpassed earlier virus variants (21). Given these genetic, epidemiological, transmission, and immune evading disparities, the World Health Organization (WHO), COVID-19 Genomic UK consortium, and the US Centres for Disease Control and Prevention (CDC) have classified the SARS-CoV-2 lineages into Variants Of Concern (VOCs) and Variants Of Interest (VOIs) (22, 23). VOCs are variants that induce more severe diseases with higher transmission rates and immune evasion potential. They were further subclassified into Alpha, Beta, Gamma, and Delta (Table 2). Less common variants, albeit with a similar mutational burden to the VOCs, were categorized under the “VOIs” classification (22, 23). The Omicron variant, which emerged in late 2021, infected primary adult upper airway tissue relative to Delta, resulting in higher transmissibility (24, 25).

**Table 2:** Recent evolution of SARS-CoV-2 variants (50, 300–302).

**Name**	**Location and time of first identification**	**Mutations**	**Effect**
Alpha	UK—late December 2020	17 mutations in viral genome; N501Y shows increased affinity of S protein to ACE2 receptors, enhancing viral attachment and cell entry	Increased severity
Beta	South Africa—December 2020	9 mutations in spike protein; K417N, E484K, and N501Y are in receptor binding domain (RBD) and increase binding affinity for ACE2 receptors	Increased risk of transmission; Reduced neutralization
Gamma	Brazil—early January 2021	10 mutations in the S protein; L18F, K417N, and E484K in RBD	Reduced neutralization
Delta	India—December 2020	10 mutations in S protein	Rapid spreading; reduced affinity for ACE2
Omicron	South Africa—November 2021	37 mutations in S protein; 15 mutations on RBD	13-fold increase in viral infectivity; increased ACE2 binding affinities
JN.1	August 2023	39 mutations in S protein	Rapid spreading; higher immune evasion property
KP.3	Feburary 2024	S protein mutations	–
KP.3.1.1	March 2024	S protein mutations	–
LB.1	April 2024	S protein mutations	–
XEC	June 2024	S protein mutations	Rapid spreading
LP.8.1	July 2024	S protein mutations	–
NB.1.8.1	January 2025	S protein mutations	–

The Omicron family of SARS-CoV-2 sub-variants represents the most genetically diverse and rapidly evolving lineage of the virus since its emergence in late 2021. The progenitor variant, B.1.1.529 (Omicron), was initially identified in South Africa. It exhibited over 50 mutations on the spike protein, which enhanced its transmissibility and increased its ability to evade immune responses (19).

Throughout 2022, sub-lineages including BA.1, BA.2, and BA.5 quickly replaced the Delta form, which was previously the most prevalent. As the virus developed further, novel sub-variants including EG.5 (Eris), JN.1, and XBB.1.5 (Kraken) appeared. These sub-variants all demonstrated additional adaptations in immune evasion and infection rates while generally retaining milder clinical presentations, particularly in vaccinated populations (19).

By 2024–2025, JN.1 had been the most common type globally. This was followed by the appearance of NB.1.8.1, which caused concern because of its quick expansion in China and its discovery in foreign visitors. Derived from JN.1, NB.1.8.1 and other more recent sub-lineages carry on the pattern of increased transmissibility and partial resistance to neutralizing antibodies. A runny nose, fatigue, fever, headaches, coughing, sore throat, dyspnoea, digestive problems, and loss of taste and smell are some of the symptoms (19). Table 2 outlines an up-to-date summary of the original prevalent and dangerous variants, and the newest prevalent variants that are either of interest or of concern.

## Etiology

The main risk factors of SARS-CoV-2 infection are male gender, older age, lower socioeconomic status, ethnicity, chronic disease, and country of birth [reviewed in ref. (26)]. It is essential to recognize that COVID-19 is an etiology to many diseases, such as cold agglutinin disease (CAD), autoimmune hemolytic anemia (AIHA), acute kidney injury (AKI), and more (27, 28). Identifying and treating these co-factors during viral infection may improve patient recovery. Many studies have shown that pre-existing conditions are risk factors for contracting COVID-19 and exacerbate symptoms during viral infection. Age and diabetes correlated with severe symptomology. Numbers were even higher when diabetes coexisted with comorbidities like hypertension (29, 30). Cardiovascular, respiratory, or hypertension comorbidities, those requiring ventilation, and those with elevated sequential organ failure assessment (SOFA) scores also have a higher likelihood not only of hospitalization in the ICU with acute respiratory distress syndrome (ARDS) but also a higher risk of death when contracting COVID-19 (31). A case study from early 2020 found that patients with bacterial or fungal co-infections were more likely to experience complications (32). Epilepsy appears to be more commonly associated with an increased risk of neurological manifestations and abnormal electrical activity in the brain when diagnosed with COVID-19 (33). Infected patients with chronic kidney disease often have the most impaired immune response out of patients with other comorbidities (34).

SARS-CoV-2 serves as an etiology itself to numerous inflammatory responses and disorders. Infected persons commonly experience cough, fatigue, and fever, although asymptomatic conditions may occur (35). The virus is known to lead to neurological syndromes, such as stroke, coma, anosmia, headache, and ageusia (35). Infections can directly affect glomeruli and renal tubules, cell-mediated immunity, and cytokine storms (36). Five to twenty-three percent of people with the coronavirus develop symptoms of acute kidney injury (AKI), which coincides with the activation of coagulation pathways, damage to the renal vascular endothelium, increased cytokines and cytokine storm, sepsis, circulatory disturbances, and hypoxemia (28). Numerous case studies show that foot manifestations (intense pain, numbness, itching, swelling, rashes, and/or altered gait) are symptoms of COVID-19 infection (37). Additionally, infection has been shown to damage the cardiovascular system, causing myocardial infarction, heart failure, cardiogenic shock, myocarditis, microthrombi, and stress cardiomyopathy (38).

## Pathogenesis of SARS-CoV-2

A typical SARS-CoV-2 infection involves viral invasion and multiplication, dysregulated immune response, multiple organ damage, and recovery (39). As the virus penetrates the host cells, it replicates, assembles, and most likely affects alveolar epithelial cells, leading to bilateral diffuse alveolar damage, vascular obstruction, patchy inflammatory cellular infiltration, intra-alveolar edema, hemorrhage, proteinaceous exudate, denudation, and reactive pneumocyte hyperplasia (40, 41). ARDS, sepsis, and multiple organ dysfunction syndrome (MODS) are all caused by the simultaneous release of numerous Pathogen-Associated Molecular Patterns (PAMPs) and Damage-Associated Molecular Patterns (DAMPs), eliciting immune cell infiltration. Although most patients eventually recover, some may experience chronic disease, persistent inflammation, systemic immunosuppression, and development of catabolic syndrome (42).

### Host cell invasion by SARS-CoV-2

**Figure 1:**
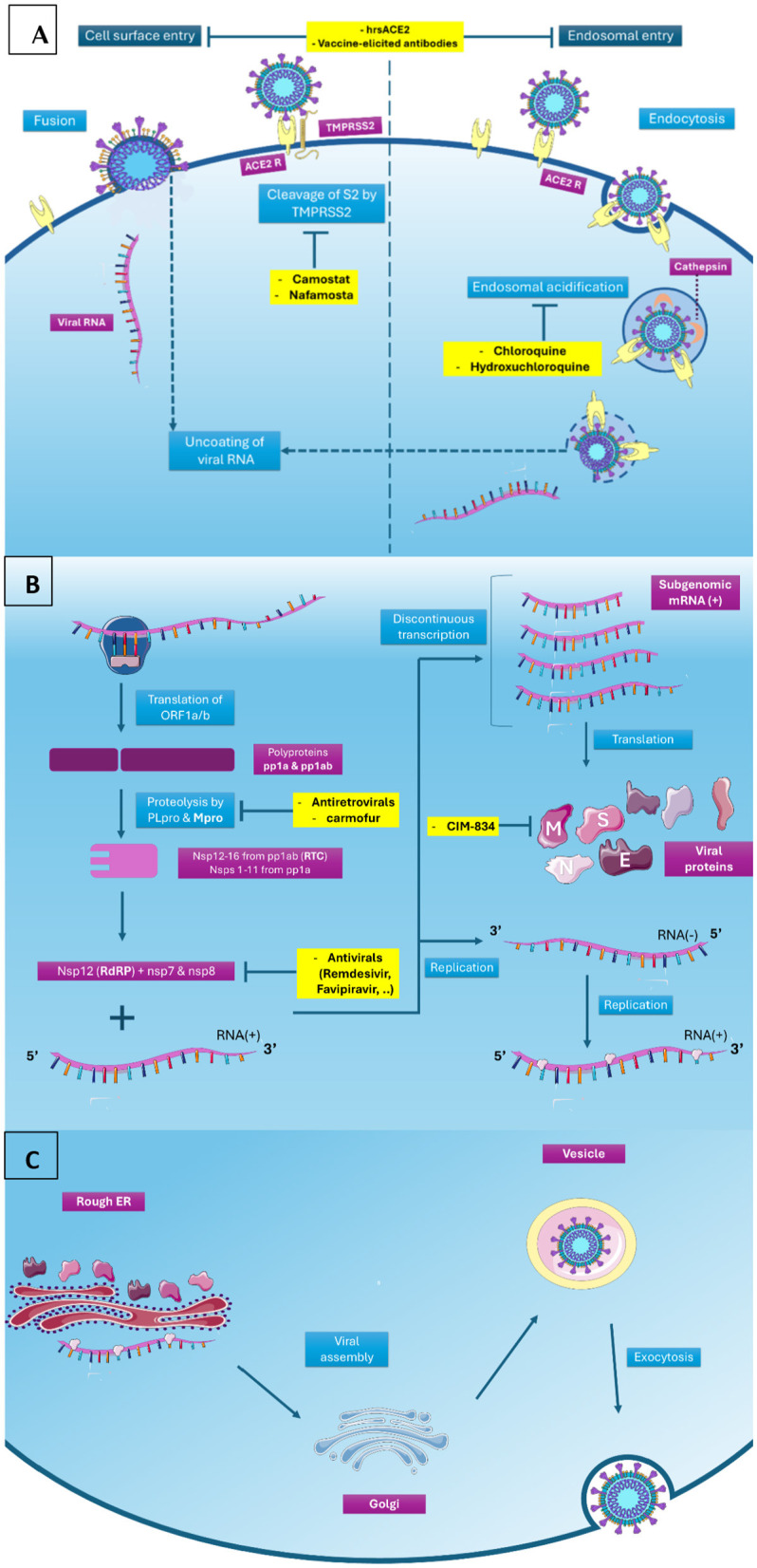
SARS-COV-2 life cycle and therapeutic targets. **(A)** Attachment and entry: SARS-CoV-2 can enter host cells either via endosomes or via fusion between the viral envelope and the host cell membrane. Both routes involve viral S protein binding to the entry receptor ACE2 on host cell, triggering conformational changes in S1 subunit and exposing the S2′ cleavage site in the S2 subunit. In the presence of TMPRSS2, it cleaves the S2′ site directly at the cell surface (cell surface entry). While in the absence of TMPRSS2 (endosomal entry), the virus–ACE2 complex is taken into the cell through clathrin-mediated endocytosis. consequently, cathepsins in acidic endolysosomes cleave the S2′ site. Fusion initiates as S1 dissociates from S2, inducing major conformational changes in the S2 subunit, and leading to protrusion of the FP forward into the target membrane OR as the cleavage of the S2′ site exposes the fusion peptide (FP). Viral RNA is released into the cytoplasm after formation of fusion pores. (1) Agents that disrupt interaction between the S protein and ACE2 receptor can inhibit both entry pathways. Ex: human recombinant soluble ACE2 (hrsACE2), an ACE2 mimetic, have been shown to inhibit the attachment of the virus to the cells by misleading viruses to bind to a pseudo receptor, and antibodies released by vaccination block virus binding to ACE2. Serine protease inhibitors camostat mesylate and nafamostat restrict the TMPRSS2-mediated entry pathway. Antimalarials hydroxychloroquine and chloroquine block endosomal acidification, which is necessary for cathepsin activity, consequently limiting the cathepsin-mediated entry pathway (103). **(B)** Translation and transcription: The translation of viral ORF1a/b by host ribosomes produces two polyproteins, pp1a and pp1ab. proteolytic cleavage of pp1a and pp1ab by 2 cysteine proteases located within nsp3 (PLpro) and nsp5 (Mpro) releases 16 non- structural proteins, nsp1–11 (from pp1a) and nsp1–10, and nsp12–16 (from pp1ab), which are important for the viral replication and packaging of a new generation of viruses (103). Nsp12–16 form the replication-transcription complex (RTC). Nsp12 (RdRP), alongside its two cofactors nsp7 and nsp8, drives replication and transcription to produce full-length genomic and sub-genomic RNAs (sgRNAs). Nsp14 (Proofreading exonuclease) ensures error correction. Nsp13 (Helicase/NTPase and RNA 5′-triphosphatase activity), Nsp14 (N7-methyltransferase), and Nsp16+ Nsp10 (2′-O-methyltransferase complex) are required for Viral RNA capping to mimic host mRNA, increasing RNA stability and immune evasion (103). Ritonavir and Lopinavir (Antiretrovirals): decrease the viral load by inhibiting viral protease Mpro. Carmofur (1-hexylcarbamoyl-5-fluorouracil): an approved anticancer drug, have been shown to potently inhibit Mpro activity in vitro. (3) Remdesivir, Favipiravir, Galidesivir, and Levovir (Antivirals): halt the replication of the viral genome within the host cell by inhibiting the viral coded enzyme RNA-dependent RNA polymerase (RdRp). Ciclesonide (Glucocorticoid): It inhibits viral replication by interacting, directly or indirectly, with viral nsp15 (103). **(C)** Viral RNA and structural proteins (M, E, N, and S) are assembled into virions in host ER and Golgi apparatus, where M + E proteins form the viral envelope and N protein enhances RNA packaging. The mature viruses get released via exocytosis to infect other cells (103). CIM-834: binds and stabilizes the M protein in its short form in vitro, thereby inhibiting its oligomerization, which is crucial for successful viral particles assembly (103). ACE2 R, Angiotensin-converting enzyme 2 receptor; TMPRSS2, Transmembrane protease, serine 2; ORF, open frame reading; Nsp, non-structural proteins. Mpro, main protease; PLpro, papain-like protease; RdRP, RNA-dependent RNA polymerase; Rough ER, rough endoplasmic reticulum.

SARS-CoV-2 has four main structural proteins that contribute to viral infection. The spike (S) protein mediates the binding of the virus to the ACE2 receptor on host cells, the envelope (E) protein activates immunopathology in infection, the membrane (M) protein stabilizes the viral structure and its budding and release, and the nucleocapsid (N) protein takes part in viral replication (43). Three non-structural viral proteins play a critical role in viral replication and the immune response: RNA-dependent RNA polymerase (RdRp), main protease (Mpro), and papain-like protease (PLpro) (44) (Figure 1). RdRp plays a significant role in the replication of -ssRNA viruses, and Mpro and PLpro are cysteine proteases that process the polyproteins (45).

First, the viral S protein, primed by host transmembrane protease serine 2 (TMPRSS2), binds the ACE2 host cell receptor (46) (Figure 1). The S protein has two subunits: S1 and S2. The S1 subunit contains the ACE2-binding site, and the S2 subunit causes fusion between the viral envelope and the host cell membrane (46). The S1/S2 subunit boundary encodes a furin cleavage site, increasing the viral transmission rate and pathogenicity (46). Viral entry promotes cytokine release and contributes to cytokine storm (47). When infected with COVID-19, the host humoral response lasts ten or more days through IgA, IgM, and IgG antibodies (43). Detecting these three antibodies has demonstrated high accuracy in diagnosing COVID-19 (48).

Research shows a 79% homology between SARS-CoV and SARS-CoV-2 (49). Some studies indicated that the receptor binding domain (RBD) of SARS-CoV and −2 are identical. In contrast, other studies have demonstrated that SARS-CoV-2 has an affinity 5–20 times greater than SARS-CoV for the ACE2 receptor due to five amino acid residue substitutions that form additional hydrogen bonds and salt bridge interactions (43, 50). The higher ACE2 receptor concentrations lead to more efficient viral entry and higher replication rates (43). Lung and small intestine epithelial cells with high ACE2 expression are first infected (51, 52). Nineteen to twenty residues of ACE2, mainly in the N-terminal helix, are in contact with 17–19 residues of the SARS-CoV-2 RBD and interface through 13 hydrogen bonds and two salt bridges (53). Y505A is the most critical residue for SARS-CoV-2, as a single point mutation (Y505A) of the viral RBD was enough to eliminate any possible binding to the ACE2 receptor (53). SNPs in ACE2, such as the absence of ACE2 N546 in three out of 10,000 humans, alter expression and glycosylation states, which could raise the severity of symptoms (50, 54).

SARS-CoV-2 can enter the cell via endocytosis or by direct fusion with the plasma membrane, depending on the activation of the S-protein by the cellular TMPRSS2 protease (55). Upon entry into the host cell, viral RNA is released and subsequently replicated by biosynthesis, wherein maturation follows, and virions are released from the cell (56) (Figure 1). Generically, micro RNAs (miRNAs) are known to either increase or suppress viral RNA replication. Through RNA interference, miRNAs bind to complementary sequences of viral RNA and induce the formation of a silencing complex that inhibits the viral RNA and its protein expression. Long non-coding RNAs (lncRNAs) regulate gene expression in cytokine storms and can affect the expression of downstream targets by sponging miRNAs (56).

### Immune response to SARS-CoV2 infection

The components of the host immune system seek to clear foreign microbes. While innate immune cells work broadly to eradicate invading particles, adaptive immune cells produce antibodies that recognize and deactivate extracellular antigens. In contrast, T-cells specifically target and kill infected cells or help other immune cells remove the infection. To eradicate the invading viruses, host innate immune cells secrete many chemical messengers, including proinflammatory cytokines and chemokines (57). The release of type I interferons (IFNs) among other proinflammatory cytokines is mediated through the activation of Pathogen Recognition Receptors (PRRs) by SARS-CoV-2 genetic material in a mechanism regulated by both Signal Transducer and Activator of Transcription 1 (STAT1) and Myeloid Differentiation Main Response Protein (MyD88) (58). Retinoic acid-inducible gene I-Like Receptors (RLRs) also sense the viral replicative intermediates and trigger IFN response that activates lung proinflammatory macrophages, which induce vascular leakage (58). The release of Viroporin-3a, an integral protein on the surface of SARS-CoV-2, activates nucleotide oligomerization domain (NOD)-like receptor protein 3 (NLRP3) inflammasome pathway and IL-1β secretion from bone marrow-derived linking PAMP-PRR signaling in macrophages with cytokine release (59). On the other hand, damaged/dead host cells release their endogenous contents, which are recognized by PRRs, further aggravating the inflammatory response and creating a vicious cycle (60). Epithelial cells can fuel this inflammatory niche by producing other cytokines like MCP1/CCL2, CCL3, CXCL1, CXCL3, CXCL10, IL-8/CXCL8, IL-1β, and TNF-α, which draw macrophages and induce further tissue damage (61). These epithelial cells are spatially located in the vicinity of the HLA-DR-low/S100A-high macrophages, resulting in sustained contact between epithelial and immune cells (61). Eventually, tissue-quiescent macrophages are either polarized toward an inflammatory phenotype or replenished by infiltrating pro-inflammatory macrophages (62).

Uncertainty surrounds how T cell subsets contribute to SARS-CoV-2 pathogenesis and resolution. Mounting evidence suggests that the S protein is the chief antigenic protein that activates both humoral and cytotoxic T-cell responses. Xu and his colleagues showed that SARS-CoV-2-infected patients had significantly lower proportions of circulating CD4+ and CD8+ T lymphocytes, albeit being in a hyperactivation state. Moreover, higher percentages of cytotoxic CD8^+^T and T helper 17 (Th17) circulating cell subsets were linked to a severe inflammatory response (63). Along with the decline in peripheral T cell count, Diao et al. reported that SARS-CoV-2 also resulted in functional exhaustion of the remaining T cells. Given the imbalance between different T cell populations in many inflammatory disorders, more effort is needed to analyze the imbalance of the Th1/Th2 and Th17/regulatory T cell ratios in SARS-CoV-2 and disease progression (64). Specific IgA, IgM, and IgG responses comprise the host humoral defense mechanisms against the SARS-CoV-2 virus. Most COVID-19 patients develop a specific antibody (Ab) response 10 days or less after the onset of symptoms (65). Virologically neutralizing Abs are essential for the elimination of viruses. Tian et al. evaluated the newly developed 2019 coronavirus S protein (2019-nCoV) cross-reactivity against anti-SARS-CoV2 Abs (66). Collected from recently recovering patients, the study's authors demonstrated that the human CR3022 anti-SARS-CoV-2 monoclonal antibody bound to the 2019-nCoV RBD without overlapping with the ACE-2 binding site (66). They concluded that the CR3022 clone can be therapeutically used to tackle the current and emerging SARS-CoV-2 infections (66). In COVID-19-recovered patients, Ni and his group studied the cross-activation of SARS-CoV-2-specific humoral and cellular immunity (67). In this small cohort, neutralizing IgM and IgG against N protein and S-RBD had higher titer, which remained for at least 2 weeks. *In vitro*, assays revealed a shift toward high IFN-γ secreting N-specific T cells in SARS-CoV-2 convalescent subjects (67). Strikingly, SARS-CoV-2 neutralization Abs were statistically correlated with the number of N-specific T-cells, suggesting a dynamic association between humoral and cellular immunity during the battle against SARS-CoV-2 infection (67).

### Hypercytokinemia and organ damage

In high-risk patients, SARS-CoV-2 infection may result in systemic and pulmonary inflammation and eventually MODS. The primary indicator of severe or critical SARS-CoV-2 pneumonia is organ failure (68). Patients with severe SARS-CoV-2 infection most frequently experience ARDS, shock, acute myocardial injury, liver injury, kidney injury, and MODS (68). It is commonly accepted that severe ARDS is the primary pathophysiology of critical COVID-19. The direct harm caused to target cells by SARS-CoV-2 and incorrect host responses, such as the immune-inflammatory response, may elicit multi-organ involvement (69). SARS and SARS-CoV-2 infections are characterized by fulminant MOD and high proinflammatory cytokine responses that may lead to hypercytokinemia, also known as cytokine storm, a phenomenon characterized by uncontrolled inflammation (70). Lung macrophages are believed to be the leading cause of hypercytokinemia by producing IL2, IL7, IL10, G-CSF, IP10, MCP1, MIP1A, and TNFα (71). In a retrospective study led by Chen and his colleagues, patients with moderate and severe SARS-CoV-2 infections had higher serum IL-2R, IL6, IL10, and TNFα cytokine profiles together with high risk of developing secondary hemophagocytic lymphohistiocytosis (72). Exaggerated and uncontrollable inflammation brought on by viral replication can harm the heart, liver, kidney, and central nervous system, resulting in sepsis, shock, or multiple organ failure (73). Higher numbers of tissue-infiltrating Th17 and cytotoxic T lymphocytes and acquired immune-induced proinflammatory responses may play a significant role in hypercytokinemia-induced tissue damage (71). Additionally, local production of proteolytic enzymes like elastase, collagenase, cathepsin, and matrix metalloproteinase may contribute to tissue and organ damage (73). In patients with SARS-CoV-2 infection and other inflammatory pathological processes, organ malfunction and tissue damage may result from the oxidative stress inflicted by the inflammatory macrophages and polymorphonuclear cells (74).

Zhou *et al* reported that, in COVID ICU patients, the aberrant pathogenic T helper 1 cells secreting higher levels of GM-CSF alongside the increased percentage of CD14^+^CD16^+^ inflammatory monocytes secreting a significantly higher levels of IL-6 could result in an inflammatory storm and severe lung damage, leading to lung functional disability, ARDS, and quick mortality (74). Xu et al. pointed out that SARS-COV-2 can lead to liver injury directly by infecting liver cells or indirectly by excessive inflammatory responses (74). Similarly, several mechanisms were reported to be involved in COVID-19-associated kidney injury, including the direct viral infection and the excessive inflammatory responses (74). Additionally, Mao et al. observed that markedly elevated proinflammatory cytokines in patients with severe COVID-19 may exert neurotoxic effects, contributing to neurological manifestations and skeletal muscle injury (74).

### Immunosuppression

Lymphopenia, resulting from the increased lymphocyte infiltration into affected tissues, is a frequent symptom in COVID-19 patients and is associated with advanced disease stages and mortality. This state of immunosuppression can inhibit viral eradication and increase the likelihood of secondary infections, especially in SARS-CoV-2-hospitalized patients (5–15.5%) (75). A meta-analysis of 3,448 patients from 28 studies revealed that 14.3% of COVID-19 patients had subsequent secondary bacterial infections. These patients had higher SARS-CoV-2 viral loads, longer viral shedding, and reduced immune responses (76). This has a potential impact on cancer patients who are already or are going to receive anticancer drugs alone or in combination with immunosuppressives (77).

## Pathophysiology of SARS-CoV-2

### Respiratory system

There are three stages to the SARS-CoV-2 infection of the respiratory system. The first phase, which may be asymptomatic, affects cells lining the nasopharyngeal cavity without triggering a robust immune response (78). During the second stage, major airways are infected, and an early inflammatory reaction starts, whereas the virus invades type I and II pneumocytes in the third stage (78). While small cuboidal type II pneumocytes include “lamellar bodies” that secrete essential lung surfactants for normal alveolar functions, type I pneumocytes have a typical epithelial shape (79). Infection of type II pneumocytes upregulates antiviral genes, such as IFNs and specific ILs, and downregulates surfactant production genes (80). Escalated by high viral load, signaling in type II pneumocytes draws immune cells from the circulation and activates tissue-resident immune cells to activate pyroptosis in infected cells (78). This reaction worsens type I pneumocyte injury, causing the alveolar barrier to break down and cellular components to infiltrate the airways. The interstitium between the capillaries and the alveolar chamber expands as the immune response damages the alveolus and causes it to fill with a mixture of plasma exudate, dead cells, virus particles, inflammatory cells, and fibrin. Consequently, gas exchange is impaired, resulting in respiratory dysfunction that gives the disease, SARS, its name (78). Initially, it was believed that younger people are less likely to get infected because the lung tissue expression of ACE-2 increases with age. However, the data appears inconclusive due to the lack of age-dependent variation in the receptor expression (81, 82).

### Cardiovascular system

Thrombotic events, including disseminated intravascular coagulation and venous thromboembolism, are the primary comorbidities in COVID-19 patients (83). This is because SARS-CoV-2 infection induces severe hematological changes like leukopenia (80%), thrombocytopenia (30%), and anemia (12%) (84) together with a rise in inflammatory cytokines including IL-6, IL-2, IL-7, interferon-ɤ, and TNFα, and high plasma levels of the thrombotic risk markers D-dimer, procalcitonin, and C-reactive protein (84). These biochemical alterations induce chronic hypertension that, in combination with the prothrombotic state, dramatically raises the risk of thromboembolism (85). Co-factors, such as cardiovascular diseases (coronary heart disease) or chronic diseases, such as type-2 Diabetes Mellitus (T2DM), are correlated with poor outcomes (84). Importantly, autopsy sequencing proved that SARS-CoV-2 infection is not a direct cause of the heart failure observed in terminally sick patients (85). Instead, the SARS-CoV-2-induced systemic inflammation seems to be the primary cause of cardiac dysfunction, with tissue endothelia's key role in disease pathophysiology (84).

### Gastrointestinal tract

#### Dental complications

Originating from sputum or nasal cavities, SARS-CoV-2-infected individuals with typical symptoms had detectable viral loads in their saliva (86). Sequencing of tissue biopsies confirmed the positivity of the SARS-CoV-2 receptor in all oral cavity cell types and salivary gland epithelia of patients with COVID-19 infection, suggesting that the mouth may serve as both a point of infection and a virus reservoir (87) and explaining specific COVID-19 related symptoms like dysgeusia and ageusia (88). Over months, numerous lesions, including ulcers, erythema, and spontaneous bleeding, have been reported in the oral cavity of infected patients. Risk factors for dental complications included implants, xerostomia, poor oral hygiene, opportunistic infections, stress, vascular impairment, and COVID-19-related hyperinflammatory response (88).

#### Hepatic complications

##### Impact on disease course

According to Chan SL & Kudo M, different reports of COVID-19 stated that 2–11% of COVID-19 patients had comorbid liver diseases (89). In patients with compromised hepatic reserves, SARS-CoV-2 may cause additional hepatic injury or hepatic decompensation. Additionally, SARS-CoV2's potential immunosuppressive property might lead to viral reactivation in COVID-19 patients with chronic viral hepatitis (89) (Figure 1). Antiretrovirals such as lopinavir/ritonavir, antifungal agents, antibiotics, and other drugs required to treat SARS-CoV-2 and its associated inflammation may cause drug-induced liver injury or reactivate hepatitis B virus (89, 90). This drug-induced cytotoxic effect, alongside the suggested SARS-CoV-2 viral replication in hepatocytes, can damage the remaining parenchyma in patients with liver cirrhosis and worsen their prognosis (90). The COVID-19 lockdown caused social isolation, sedentary behavior, and an increase in processed food consumption, all of which are unfavorable metabolic antagonists in non-alcoholic fatty liver disease (NAFLD) patients (91).

##### Impact on patient care

During the physical distancing at the beginning of the COVID-19 pandemic, resources were diverted for the sickest patients to decrease hospital utilization. Stable patients' routine care was put off, lessening the spread of SARS-CoV-2 (92). Hepatocellular carcinoma (HCC) and varices screening were converted from all-comers to high-risk patients only. As a result, these delays increased the risk of variceal hemorrhage and, in approximately 25% of patients with biologically aggressive disease, raised the likelihood of HCC being discovered at a later stage (92). They also anticipated that “returning to normal” after physical distancing would be difficult with more decompensations and morbidity and care systems being overburdened by the backlog of put-off care. Patients who were incorrectly classified as low risk in the first wave would start to present with decompensations, requiring more resources and raising the possibility of cascade deferrals for the presently asymptomatic patients. Complications of delayed screening procedures, failure to diagnose HCC at earlier stages, and medical therapy complications due to lack of timely lab work all would lead to the transformation of the curable into the incurable (92). In an Italian study that included 42 HCC patients, it was reported that the pandemic caused a treatment delay of 2 months or longer in 11 patients (26%) & change in treatment plan from surgical resection to thermal ablations in 3 patients. It is unknown whether or not these delays and modifications will affect the long-term outcomes for these patients (93). Several factors contributed to a dramatic decrease in the number of liver transplantation procedures during the COVID-19 pandemic, including the cancellation of elective therapeutic procedures such as living donor liver transplantations, uncertainty regarding the feasibility of intensive care beds, blood products or ventilators required to accept deceased donor's organ for the waitlisted patients, uncertainty regarding the test-accuracy for SARS-CoV-2 and its transmission, conversion of several transplantation centers into COVID-19 units, and the quarantine travel restrictions leading to increase in waitlist mortality (92). Viral hepatitis was no exception in terms of the redirected attention and resources from other diseases to COVID-19. Since most elimination programs for viral hepatitis were put on hold or stopped altogether, the WHO's goal of eliminating HBV and HCV and decreasing their mortality by 2030 has been hindered. One study proposed that a 1-year delay in diagnosis and treatment of viral hepatitis will globally result in 72,300 deaths from HCV and an additional 44,800 HCC cases in the next 10 years (91).

#### Lower GIT complications

Approximately 20% of SARS-CoV-2-infected patients exhibit gastrointestinal symptoms such as nausea, vomiting, and diarrhea; this number jumps to 50% in hospitalized patients. It is now thought that SARS-CoV-2 directly infects enterocytes, with the intestinal epithelium having the highest ACE-2 expression level (94). This might explain why seriously diseased patients witness symptoms resembling those of inflammatory bowel diseases like Crohn's disease and irritable bowel syndrome and justify the presence of the viral genetic material in the feces of infected patients even if they didn't develop typical SARS-CoV-2 symptoms (94). Moreover, changes in the immune microenvironment within gastrointestinal (GI) tract organs and their microbiota resemble the proinflammatory characteristics of SARS-CoV-2 -infected lungs (94, 95).

### Central nervous system

Approximately 30% of infected individuals experience neurological symptoms, which can range from minor issues like headaches to serious ones like cerebrovascular infarction. Three potential causes of COVID-9's pathophysiology manifestation in the neurological system include chronic inflammation, underlying coagulopathy-induced thrombotic events, and direct infection of nervous system cells. There is evidence that SARS-CoV-2 crosses the blood-brain barrier since it has been detected in the patient's brain tissue, and upregulation of interferon signaling pathways of the neurovascular compartments has been shown (96). Furthermore, brain capillary endothelial-like cells were infected in *in vitro* models. Besides severe brain tissue infections, taste and smell loss are typically categorized as neurological complaints.

### Dermatologic manifestations

The COVID-19 cutaneous symptoms are primarily divided into six categories of injury: confluent erythematous/maculopapular/morbilliform rash, urticarial rash, papulovesicular exanthem, chilblain-like acral pattern, livedo reticularis/racemose-like pattern, and purpuric pattern (97). These injury patterns, which frequently include endothelial damage and perivascular inflammation of dermal arteries, are likely the result of cutaneous reactions to circulating viral antigens. Erythematous or maculopapular eruptions appear after the onset of systemic symptoms in nearly 50% of patients with SARS-CoV-2-related rashes. These lesions typically have pruritic symptoms, symmetrical distribution over the trunk, and centrifugal propagation. Despite the paucity of data, these eruptions appear to be caused by vascular damage and frequently have perivascular lymphocytic or neutrophilic infiltrates (97). The trunk and limbs are where urticarial rashes are most noticeable, and they may also be accompanied by angioedema or urticarial vasculitis (97). They occasionally appear before pulmonary illness presentations in individuals, although they typically appear simultaneously as other systemic symptoms. Papulovesicular exanthems frequently have vesicles dispersed around the trunk, either with or without pruritus. Biopsies show endothelial inflammation in dermal arteries and epidermal acantholysis with inflated keratinocytes (98). Endothelial damage and thromboses are frequently present in eruptions with the acral pattern, livedo reticularis, and racemose-like patterns. These eruptions may be brought on by IFN-mediated inflammatory responses or endothelial injury related to circulating viral particles (98). Chilblain-like lesions manifest as violaceous plaques that primarily affect the feet but can also occasionally involve the hands or ears. Unlike other COVID-19 dermatologic manifestations, these lesions are typically unpleasant or itchy and tend to affect people with little or no systemic symptoms (98). The livedo reticularis/racemose-like pattern is characterized by lace-like patches with a dusky blue discoloration and most likely signifies sluggish blood flow brought on by thrombosis of small superficial veins in the dermis (98).

## Pregnancy

It is crucial to assess the possibility of severe consequences from SARS-CoV-2 infection in pregnant women and fetuses/newborns. Clinical traits of pregnant COVID-19 carriers have been examined in several original investigations and systematic reviews (99). Notably, most studies compare pregnant women with COVID-19 to non-pregnant women of reproductive age in terms of clinical features and death rates. In contrast, SARS-CoV and other respiratory viruses have shown different symptoms (100). Some have hypothesized that this is due to the natural immunological adjustments during pregnancy, preventing COVID-19 from escalating to the hyperinflammatory phase. Although there is evidence of moderate COVID-19 in pregnant patients, a new analysis by the CDC implies that pregnant women may be at a higher risk for more severe consequences, suggesting that a higher percentage of pregnant women with COVID-19 require hospitalization compared to non-pregnant women (101). However, the lack of information indicating whether hospital admission was brought on by COVID-19 sickness or symptoms related to pregnancy limits this study and makes interpretation more difficult (101). In addition to these studies, there is mounting evidence that pregnant SARS-CoV-2-infected women experience enhanced rates of miscarriage and hypertension, indicating placental involvement (100). Most research has found no evidence that the placenta contains SARS-CoV-2 RNA. However, a recent case report revealed SARS-CoV-2 in the syncytiotrophoblast cells of a COVID-19 patient who was pregnant and had preeclampsia in the second trimester of the pregnancy. It also indicates that there is a distinct association between the laboratory profile seen in preeclamptic pregnant individuals and COVID-19, raising issues about common disease mechanisms (100). The latest research suggests that vertical transmission of SARS-CoV-2 is improbable, which is an important final point to make. However, as previously mentioned, SARS-CoV-2 can damage the placenta dramatically and consequently adversely affect fetal development. To more fully understand the clinical profile of COVID-19 during each trimester of pregnancy, additional study is required (100).

## Pediatrics

In a case study of more than 2,000 children with suspected or confirmed COVID-19, only 0.6% of the symptomatic individuals proceeded to ARDS or Multiple Organ Failure (MOF), while 5% exhibited dyspnea or hypoxemia (102). Interestingly, the laboratory profile of pediatric COVID-19 is distinctive from that of adults in cases with mild infections. However, a meta-analysis of 24 studies, including 624 pediatric cases with PCR-proven COVID-19, has shown common laboratory abnormalities in moderate and severe disease courses regarding CRP, procalcitonin, and LDH but not in lymphocyte count (102). One explanation of such discrepancy might be the disparate standards employed to interpret laboratory testing in pediatrics (102). Moreover, a recent study of a small cohort of previously healthy children and teenagers who developed an inflammatory profile linked to COVID-19 described a special cytokine pattern characterized by elevated IL-6 and IL-10 production, as well as increased IFN signaling components with no increase in TNF-α which is common among adults (103). Pediatric COVID-19 was also associated with GI symptoms and cutaneous signs (104). In response to the unmet need to accurately identify pediatric COVID-19, WHO and other regulatory authorities have created a preliminary case description known as Multisystem Inflammatory Disorder in Children and Adolescents (MIS-C) (103). Further studies are necessary to determine the applied protective measures against pediatric COVID-19 and the underlying causative mechanisms behind MIS-C in children.

## Lines of treatment for COVID-19

### SARS-CoV-2 lifecycle and therapeutic targets

For most people, COVID-19 can be treated like a common cold: rest, fluid intake, and over-the-counter medicine to reduce symptoms like fever and congestion. For those who experience more severe symptoms that require hospitalization, respiratory support is the primary focus of treatment with therapeutics. Multiple clinical trials were performed to test the efficacy of different treatment options, including antiviral therapies, corticosteroids, immunotherapies, dietary supplements, and more.

#### Antiviral therapeutics

Antiviral therapeutics were investigated to treat COVID-19 as they function in a variety of mechanisms (105). Depending on the disease severity, infection can be divided into three stages: non-severe, severe, and critical. In the non-severe stage, viral replication occurs enough that antiviral therapeutics can treat the infection (106). These antiviral agents can be categorized according to their mechanism of action: those that inhibit viral entry into host cells, those that block viral replication, and those that modulate the immune response. Among the drugs that specifically target viral replication are Paxlovid, remdesivir, molnupiravir, and ribavirin (106).

Remdesivir, in particular, has demonstrated clinical benefits (Figure 1). When used in combination with supportive care, it has been associated with shorter hospital stays and a significantly reduced mortality rate (105). In a study involving non-hospitalized patients who were at high risk for developing severe disease, a 3-day course of remdesivir led to an 87% reduction in the risk of COVID-19-related hospitalization or death from any cause by day 28, compared to those who received a placebo (106). According to WHO guidelines from July 14, 2022, antiviral drugs such as nirmatrelvir-ritonavir (Paxlovid^®^) are strongly recommended. Polymerase inhibitors, such as molnupiravir and remdesivir, are weak or conditional recommendations (107). Monoclonal antibody (mAb) therapies that target viral entry into host cells include casirivimab + imdevimab, bamlanivimab, sotrovimab, and bebtelovimab. These agents have been used both for treatment and prophylaxis of COVID-19. Their effectiveness can vary depending on circulating variants, and resistance can emerge, especially in immunocompromised individuals. Therefore, virological monitoring in these patients is crucial. In such cases, the use of combination monoclonal antibody therapies is highly recommended to enhance efficacy and reduce the risk of resistance development (107).

### Corticosteroids

The use of corticosteroids in treating COVID-19 has been the main question since the beginning of the pandemic. COVID-19 infection occasionally develops aggressive inflammatory responses that progress to life-threatening respiratory problems, including severe pneumonia, cytokine storm, and ARDS. It has been reported that patients in severe and critical stages have higher serum cytokine amounts (105, 108). Corticosteroids act as immunosuppressive agents for excessive inflammatory responses and prevent the progression of hyperinflammation. Once infection occurs, the virus infiltrates host cells through interactions with angiotensin-converting enzyme-2 (ACE-2) receptors, initiating an immune response. The release of cytokines in large quantities results in a cytokine storm. Cytokines such as interleukins (IL-1, IL-2, IL-6, IL-8, dan IL-12) and interferons (INF-α, INF-β, dan INF-γ) play a significant role in recruiting immune cells, including macrophages and lymphocytes (108, 109).

Corticosteroids suppress the activation and recruitment of these immune cells through their immunosuppressive effects, thereby preventing the deleterious effects of cytokine storms that can lead to dyspnea, multi-organ failure, and death (109).

Therefore, anti-inflammatory drugs such as dexamethasone, hydrocortisone, or prednisolone are essential for cytokine storm suppression (110). According to WHO guidelines on 13 January 2023, corticosteroids are recommended only in hospitalized patients (111). The data does not support usage in non-hospitalized patients (109).

### Immunomodulatory therapeutics

Regulation of immune responses against SARS-CoV-2 was one of the most important aspects of disease pathogenesis, particularly in severe and critical stages (112).

#### Interleukin-inhibitor: tocilizumab

After infection, cytokine release syndrome (CRS), with excessive immune responses and subsequent release of pro-inflammatory mediators, chemokines, and cytokines, is observed. This plays a crucial role in the severity of COVID-19. IL-6 plasma level was high, especially in severe cases. Tocilizumab is an anti-human IL-6 receptor monoclonal antibody which antagonizing the IL-6 receptor, both IL-6 soluble receptor (sIL-6R) and membrane receptor (mIL-6R) (113). It can bind the membrane-bound IL-6 receptor and inhibit signal transduction (114). Patients who developed COVID-19-related CRS could use tocilizumab with promising suppressive action (114). A cohort study discussed the pathological role for the interleukin-6 (IL-6) pathway in mediating structural and functional delirium-like phenotypes. They tested if tocilizumab is associated with a reduction in delirium/coma prevalence in critically ill patients with COVID-19. Delirium was assessed using the Confusion Assessment Method for the Intensive Care Unit (CAM-ICU), with a positive score indicating delirium. Tocilizumab was associated with a significantly greater number of days alive without delirium/coma (113).

#### Janus kinase inhibitor: baricitinib

The pathophysiology of COVID-19 involves a signaling pathway based on the Janus kinases (JAKs) and activator of transcription (STAT) pathways (115). Janus kinase inhibitors (JAKi) have been approved for various immune-mediated inflammatory diseases (115). Baricitinib is a selective JAKi inhibitor with a proven anti-inflammatory effect (303). An international, multicenter, double-blind, randomized, placebo-controlled trial evaluated the benefits and safety of baricitinib with standard of care (corticosteroids and remdesivir) for hospitalized patients. Thus, it has been suggested that baricitinib could be a good option to decrease mortality when used with standard of care (116).

A retrospective study suggested survival and safety are significantly better for baricitinib compared to tocilizumab in severe COVID-19. This study included 400 hospitalized patients with severe COVID-19 (116).

### Chloroquine and hydroxychloroquine

Chloroquine and Hydroxychloroquine had an extensive record of malaria treatment and its prevention (117) (Figure 1). Although they have been recommended in COVID-19 treatment based on experiments in Vero E6 and A549-ACE2+ cells (118–121), it has been shown that chloroquine is inactive in the relevant lung adenocarcinoma Calu-3 cell line (122, 123). Furthermore, many clinical trials have failed to prove any positive effects of these drugs on clinical status or lower mortality (124). When hospitalized patients with respiratory illness were treated with hydroxychloroquine, compared with a placebo, there was no clinical significance in improving clinical status. Therefore, these results do not support the use of these drugs in COVID-19 treatment (125). WHO guidelines on March 24, 2023, strongly recommended against administering hydroxychloroquine to prevent COVID-19-related outcomes (126).

### Anti-coagulants

COVID-19 leads to high levels of mortality because it damages some vital organs, including the cardiovascular and coagulation systems (127). In severe and/or critical stages, patients with pneumonia are highly prone to thrombotic complications (128). Anticoagulants, especially low molecular weight heparin, are associated with better prognosis in severe COVID-19 patients and decreased mortality risk (129).

### Dietary supplements

Various vitamins and minerals, such as vitamin C and zinc, support patients' immune status. Vitamin D deficiency might increase susceptibility to diseases (130). Vitamin C is an antioxidant that supports the immune system and protects against infections, which may render it an effective treatment addition (131). Zinc is a trace mineral that has a role in response to viral infection. Zinc deficiency affects immune function by impairing the formation and maturation of lymphocytes (132). A study showed that people with zinc deficiency have a higher risk of respiratory diseases (133). Vitamin D is known for its role in protecting bones, and it supports the development of some immune cells (131). A randomized clinical trial found that a high dose of vitamin D decreased CD4+ T-cell activation, worsening viral infections (134).

## Natural and synthetic compounds in the treatment of COVID-19

Since the emergence of the pandemic, scientific efforts have been devoted to working out the best treatment regimen for COVID-19. Computational analysis alongside assays on human cells and in mammalian experimental models generated an immense repository of useful information and presented novel antiviral strategies. Mining the data for COVID-19-tested drugs in 199 peer-reviewed articles published between 2020 through 2023 identified 231 natural and 169 synthetic compounds that played protective and/or therapeutic roles against COVID-19. Among these compounds, 292 were tested using *in silico* software, 50 were validated *in vitro*, 58 were tested both *in vivo* and *in vitro*, and 22 underwent clinical trials. In the following section, we sought to compile some details regarding these compounds as in Supplementary Table S1. Natural products have proven efficiency against COVID-19. Several natural products from different sources (plants, microbes, and marine) were effective against COVID-19. Natural products have been either tested as extracts or in the pure form of the compounds (135–153, 168, 170).

### *In silico* analysis

Currently, artificial intelligence is being used in the drug discovery of natural products. It has been applied to predict the macromolecular targets of natural products, the structural characterization of natural products, and the selection of natural products as drug candidates. Computational drug discovery approaches such as docking, clustering, bioactivity fingerprints, pharmacophores, and machine learning shed light on the possible targets for natural products. Moreover, the potential binding modes and the affinity of natural products could be studied via molecular docking and molecular dynamics (154). Molecular docking software, such as Auto Dock Vina, Gold, Glide, MOE, and AutoDock software, provides indispensable information about the binding of small molecules to a specific protein macromolecule in its biologically active 3D form. Given the advances of artificial intelligence and machine learning, bioinformaticians and biostatisticians were able to analyze the interaction between natural and synthetic compounds with SARS-CoV-2 key proteins.

#### M^*pro*^ target protein

Auto Dock Vina software has identified Hypericin (155), Isohypericin (155), and Anthrachinolinchinon (156) as top hits against M^pro^ protein. Docking studies run by PyRx software showed that Bemcentinib (157) effectively inhibits M^pro^. While Gold software ranked Rutin (158) and Epirubicin (158) with the highest docking scores, Glide score software flagged compounds Pectolinarin (159) and Quercetagetin (160) are potential therapeutic candidates against M^pro^. Indomethacin (161) and Bislatumlide A (162) had the top docking scores using AutoDock software. MOE software suggested Methyl rosmarinate (163) as the top hit. Finally, Acetoside and Luteolin 7-rutinoside (164) had highest docking scores using iGEMDOCK, while 2,3-Dihydroamentoflavone (165) was highlighted by MTi AutoDock software.

#### ACE2 protein

Hesperidin was the best hit using Auto Dock Vina, while Kobophenol A and Andrographolide ranked first using AutoDock (304). Furthermore, 5-O-Feruloyl-quinic acid had the highest docking score against ACE2 protein using PyRx, while Isothymol strongly bound to ACE2 protein, as suggested by MOE (305).

#### Nsp15 protein

AutoDock software identified Indomethacin (161) and Demethoxycurcumin (166) as the compounds with the highest docking scores, while Catechin-7-o-gallate (167) had the top score as calculated by PyRx. Analyzing different compounds against Nsp15 protein using AutoDock Vina detected Cnicin (168) and Glyasperin A (169) to have the highest scores.

#### Nsp16 protein

Compounds that showed the highest docking scores in the *in silico* studies were: Daphnorin (170) and Glycycoumarin (170), using Autodock Vina; Amentoflavone (171) and Baicalin (171) using PyRx; Indomethacin (161) using AutoDock.

#### TMRSST protein

The best hits are Myricetin (172), Meloxicam (172), and Columbin (172) using Autodock Vina; Withanoside-IV (173), Withanoside V (173) using LeDock (174).

#### NSP10-NSP16

The best hits are Withanolide (175) and Dolutegravir (175) using Autodock Vina and Chlorogenin (167) using PyRx.

#### PLpro protein

The compounds with highest docking scores were: Schaftoside (176) and 1-Hydroxyaleuritolic acid 3-p-hydroxybenzoate (177) using AutoDock Vina; UKR1129266 (178) using C-docker; Cichoriin (179) using COVID-19 Docking Server; Theaflavin 3-gallate (TF2a) (180) using smina server; and Indomethacin (161) using AutoDock.

#### RdRp target

Amentoflavone (167), Emblicanin A (181), and Cyanin (167), were the top 3 hits using PyRx; Taiwanhomoflavone A (182), Cnicin (168), and Nympholide A (182) were identified using AutoDock Vina; Indomethacin (161) was detected by AutoDock; Theaflavin 3,3′ digallate(TF3) (180) using smina server; Cichoriin (179) using COVID-19 Docking Server; and Tetrahydroxycurcumin (183), Andrographidine C (184), and Silibinin (185) using Glide.

#### S-protein

Glycyrrhizin (169) was the best hit using AutoDock Vina. Agathisflavone (167), and Catechin-7-o-gallate (167) had the top docking scores using PyRx. Indomethacin (161), Speciophylline (186), and Uncarine F (186) were highlighted using AutoDock. Silibinin (185) was chosen using Glide. Finally, Argus lab and CLUSPRO 2.0. identified 1-(2,3- dihydrobenzo[b](1, 4)dioxin-6-yl)-2-(furan-2-yl)-4,5-diphenyl-1 *H*-imidazole (DDFDI) (187) and Urtica dioica agglutinin (188) as top hits, respectively.

### *In vitro* studies

Alongside computer-based analyses, many research groups focused on analyzing the effect of different natural and synthetic compounds using wet laboratory settings with the hope of discovering or repurposing an existing compound to treat patients with COVID-19.

#### M^*pro*^ target protein

For example, (1R,2S,5S)-6,6-dimethyl-N-((S)-1-oxo-3-((S)-2-oxopyrrolidin-3-yl)propan-2-yl)-3-(2-(4- (trifluoromethoxy) phenoxy) acetyl)-3-azabicyclo[3.1.0]hexane-2-carboxamide (189), Silibinin (185), and GC376 (7) (190) compounds had IC_50_ of 0.015 μM, 0.021 μM, and 0.15 μM against M^pro^ target protein, respectively. Compounds GC376 (190), (1R,2S,5S)-6,6-dimethyl-N-((S)-1-oxo-3-((S)-2-oxopyrrolidin-3-yl)propan-2-yl)-3-(2-(4-(trifluoromethoxy)phenoxy)acetyl)-3-azabicyclo [3.1.0] hexane-2-carboxamide, and (1S,3aR,6aS)-2-(2-(2,4-dichlorophenoxy)acetyl)-N-((S)-1-oxo-3-((S)-2-oxopyrrolidin-3-yl)propan-2-yl)octahydrocyclopenta[c]pyrrole-1-carboxamide (189) had the lowest IC_50_ against SARS-CoV-2 targeting M^pro^ with IC_50_ of 0.70 μM, 1.2 nM, and 1.1 nM, respectively.

#### ACE-2 protein

Compounds effective against ACE-2 protein included Licarin B and Licarin A with IC_50_ of 430.11 nM and 3.59 μM, respectively (191).

#### S-protein

Pomegranate peel extract (PPE) (192), Kobophenol A (193), and Silibinin (185) have the lowest IC_50_ against S-protein with IC_50_ of 0.049 mg/ml, 1.81 μM, 0.029 μM, respectively. Cysteamine HCl (194) had the lowest IC_50_ against SARS-CoV-2 targeting S-protein.

#### PLpro target

Dihydrotanshinone I (195), Sepantronium bromide (196), Schaftoside (176), and UKR1129266 (178) had inhibitory activity against PLpro with IC_50_ of 0.59 μM, 2.47 μmol/L, 3.91 μmol/L, and 0.90 μM, respectively.

#### RdRp target

Silibinin (185) showed the lowest inhibitory activity against RdRp with IC_50_ of 0.042 μM.

#### TMRSST protein

Withanoside-IV showed *in vitro* activity against SARS-CoV-2 targeting TMRSST with %inhibition of 45.03% and 44.79% for E (envelope) and N (nucleocapsid), respectively (173).

### *In vivo* studies

Based on the successful role against COVID-19 *in vitro*, the antiviral effect of some natural and synthetic compounds was tested in experimental mammalian models.

#### M^*pro*^ target protein

Indeed, 6-Amino-1-(4-chlorophenyl)-4-(2,4-dichlorophenyl)-3-methyl-1,4- dihydropyrano[2,3-c]pyrazole-5-carbonitrile (197), FB2001 (198), Nirmatrelvir (199, 200), and Ensitrelvir (201) inhibited SARS-CoV-2 *in vivo* studies.

#### ACE-2 protein

Dalbavancin had *in vivo* activity in rhesus macaque models with *P*-value of 0.0042 (202). PLpro protein

Aurintricarboxylic acid (203) had a good inhibitory activity for PLpro protein *in vivo* studies.

#### RdRptarget

(3R,4R,5R)-2-(4-aminopyrrolo[2,1-f][1,2,4]triazin-7-yl)-2-cyano- 5-((isobutyryloxy)methyl)tetrahydrofuran-3,4-diylbis (2-methylpropanoate) (204) and Molnupiravir (205, 206) had *in vivo* activity against SARS-CoV2.

#### S-protein

H84T-BanLec (207) showed inhibitory activity against SARS-CoV-2 *in vivo* studies using a golden Syrian hamster model with a *P*-value of < 0.05 targeting S-protein.

### Clinical trials

#### Vaccines

In January 2020, Chinese scientists completed and submitted the first SARS-CoV-2 genome sequence to the NIH's GenBank database (208). Prior to the COVID-19 pandemic, the fastest vaccine development on record was for mumps in the mid-20th century, which took four years (209). Remarkably, less than a year after the SARS-CoV-2 sequence was published, Pfizer-BioNTech released the first COVID-19 vaccine for individuals aged 16 and older in December 2020 (210). This unprecedented speed was made possible by decades of prior research, substantial funding, and expedited regulatory pathways (211). Currently, five major platforms dominate COVID-19 vaccine development: mRNA, non-replicating adenovirus vectors, inactivated virus, protein-based, and DNA vaccines (212, 213) (Table 1).

Among these, mRNA vaccines are categorized into three types: circular RNA (circRNA), non-replicating mRNA (NRM), and self-amplifying mRNA (SAM) (214). These vaccines deliver mRNA into the cytosol of host cells, where it serves as a template for antigen production, triggering an immune response (215). Despite being studied for over two decades, mRNA technology faced challenges due to ribonuclease activity and mRNA instability. These issues were overcome with the development of lipid nanoparticle (LNP) delivery systems and chemical modifications to the mRNA itself (214, 216). mRNA vaccines do not require a live virus and can be designed within days of sequencing a viral genome (217). The use of LNPs has further enhanced the stability and delivery of mRNA vaccines, and their non-integrating nature eliminates the risk of infection (218).

Circular RNA (circRNA) is a covalently closed eukaryotic nucleoside ring that provides protection from degradation via endonucleases because of a lack of termini, yielding a more stable molecule than a linear RNA molecule (219, 220). CircRNA does not possess 5′ or 3′ ends, and naturally occurring circRNA can be protein-coding as well as non-coding (220). CircRNA is generated through back-splicing of pre-mRNA and, until recently, was viewed as a splicing error by-product (221). Current circRNA platforms circumvent cap-dependent translation of RNA by designing templates with either an upstream m6A modification or by introducing an internal ribosome entry site (219). Current research groups have successfully engineered circRNA COVID-19 vaccines that demonstrate prolonged antigen translation due to enhanced stability, as compared to linear mRNA vaccines (222). Interestingly, naturally occurring circRNA has been shown to alter gene expression through the regulation of nuclear transcription, RNA-binding proteins, and microRNA (223).

Both non-replicating mRNA (NRM) and self-amplifying mRNA (SAM) vaccines are linear and possess a 5′ 7-methylguanosine cap, 3′ poly(A) tail, a coding sequence inside an open reading frame, and a 5′ and 3′ untranslated region (224, 225). The 5′ cap both serves as a binding site for eukaryotic translation initiation complex cap-binding protein (eIF4E) and aids in protecting the mRNA transcript from nuclease-mediated hydrolysis (225). The poly(A) tail shields the transcript from degradation while also regulating translational efficiency based on the tail's length (215).

However, SAMs have a large open reading frame with desired antigen genes replacing structural genes from the original viral genome, which eliminates the risk of viral infection while also contributing to SAMs overall larger size when compared to NRM (226). Additionally, SAM vaccines possess viral non-structural protein genes, which encode for the replication machinery capable of producing the RNA transcript (227). Comparatively, NRM vaccines may require higher treatment doses when compared to SAM vaccines because the amount of antigen manufactured is directly dependent on the available mRNA transcripts provided by the treatment. Conversely, SAM vaccines can duplicate mRNA transcripts, leading to higher antigen expression within treated cells while also enhancing the innate immune response by propagating adjuvants (227, 228). However, NRM vaccine's advantage over its SAM counterpart stems from its shorter and more simplistic transcript, which most COVID-19 vaccines available are based on (214).

Adenoviruses (AVs) can elicit a cellular immune response, humoral immune response, or a combination of both when infecting host cells (229) (Table 1). AVs contain double-stranded DNA within an icosahedral capsid lacking an envelope (230). AV vaccines are capable of stimulating PRRs, such as toll-like receptors, which promote an innate immune response without the need for adjuvants. However, the inflammatory response is not extreme enough to cause a cytokine storm (231). Additionally, AV vaccines are incapable of replication because the E1 and E3 gene cassettes—which are imperative for viral replication—are replaced with the desired antigen sequence (232). One current COVID-19 AV vaccine is based on the type 5 vector and is designed to be inhaled, mimicking the transmission of COVID-19 into humans (233).

Inactivated virus (IV) vaccines are a traditional approach to generating an immune response that has been used in milestone treatments, such as Jonas Salk's inactivated polio vaccine (234). The immunogen for IV vaccines is the whole virus, which is inactivated with heat, chemicals, or radiation (235, 236); this confers an advantage when considering humoral responses because an abundance of epitopes from the whole virus can in turn generate many unique antibodies when compared to an mRNA vaccine that only causes expression of spike proteins (235) (Table 1).

Protein subunit vaccines utilize viral products to trigger an immune response within a host (237) (Table 1).

COVID-19 subunit vaccines typically target antigenic parts of the virus-like the RBD—or the full-length spike proteins—like S1 (238). The inclusion of only a viral product—not the entire virus—can reduce the chance of an unintended autoimmune or inflammatory reaction (237). Additionally, adjuvants can be conjugated with epitopes in a subunit vaccine to enhance the immune response (239) (Table 1).

DNA vaccines are like the previously discussed RNA vaccines. However, the nucleic acid in DNA vaccines encoding viral antigens is translocated into the nucleus for transcription after entering the host cell (216, 240). This poses a possible risk of integration into the host genome compared to RNA vaccines, which typically require freezer storage (306). DNA vaccines have improved stability and maybe a better candidate for treatment in resource-limited areas (240–242). DNA vaccines can also activate both humoral and cellular immune responses (243) (Table 1).

As of March 30, 2023, the type, number, and relative percent of the 183 candidate vaccines in the clinical phase include protein subunit (59, 32%), viral non-replicating vector (25, 14%), DNA (17, 9%), inactivated virus (22, 12%), RNA (43, 24%), and others (244). Additionally, there are 199 vaccines in pre-clinical development (244). There are currently two types of vaccines approved by the Food and Drug Administration (FDA): mRNA and protein subunit vaccine. Moderna and Pfizer-BioNTech produce the mRNA vaccine, while Novavax manufactures the protein subunit vaccine. However, J&J/Janssen produced an FDA-approved viral vector vaccine, which expired in May 2023 (245). The efficacy of Pfizer-BioNTech's vaccine is 95%, Moderna's is 94.1%, and J&J/Janssen's was 83.5% (246).

COVID-19 vaccines are diligently constructed to meet the FDA's standards; however, side effects have been reported (247). Common side effects are influenced by age, but for adults, they may include: swelling and pain at the injection site, chills, fever, tiredness, etc. (248). Albeit rare, adverse events may occur following vaccination. This includes anaphylaxis, thrombosis, myocarditis, pericarditis, and Guillain-Barré Syndrome (249).

## Artificial intelligence

The advancement of data science has significantly contributed to a breakthrough led by artificial intelligence (AI) applications such as machine learning (ML) or deep learning (DL) in various aspects, especially in the medical and pharmaceutical fields. In this context, with the viral infection outbreak causing COVID-19 in 2019, this pandemic has posed a multi-directional challenge, whether in medicine, defense, information technology, or even politics and ideologies. The COVID-19 pandemic has dramatically accelerated the adoption of AI applications in emergencies, particularly in tracing epidemiological peaks or operating within complex scenarios involving healthcare (250). The pandemic has demonstrated that the diversity of AI-driven tools' potential can eradicate health disparities on the one hand and upgrade the efficiency of health systems on the other. Studies have shown that AI interventions during the pandemic outbreak have improved various pathways, including identifying and diagnosing cases using trained deep learning algorithms, which provide results around the clock, about 135 times faster than a radiologist (251). Using neural networks and ML models also contributed to implementing preventive measures such as social distancing, identifying people without a protective face mask through their eye line, and alerting citizens to avoid crowded public places or to wear face masks (252). Furthermore, AI applications have delivered new tools to track the spread of the virus, particularly in hotspot areas. For example, in Japan, scientists developed a disease spread simulation model and GPS data miner to analyze the hotspot detection of COVID-19 infection using mobile phone location data (253). Also, a study reported that ML models have been relied upon to carry out the tasks of early identification and diagnosis of the infection based on clinical information, not CT images. One of these ML tools has been published online (https://intensivecare.shinyapps.io/COVID19/) (254). In the same context, several studies have shown the promising use of DL and ML models to detect the coronavirus by utilizing ML and DL algorithms for routine blood tests as an alternative to using the time- and effort-consuming CT scanning technique (255). However, the role of AI was not limited to detection and diagnostic tools. It also contributed to the accurate monitoring of patients during the outbreak of COVID-19. AI was used to automate the contactless scanning process of patients by using data provided by visual sensors such as RGB, Time-of-Flight (TOF) pressure imaging, or thermal cameras (256). In terms of psychological monitoring, researchers further developed previously used ML models to assess the presence of depression and anxiety and predict the most susceptible cases, especially among university students, to avoid more severe cases of mental health decline (257, 258). Furthermore, ML methods (i.e., Support Vector Machine, Artificial Neural Networks, Random Forest, Decision Tree, Logistic Regression, and K-Nearest Neighbor) also contributed to the management of health systems by predicting the clinical characteristics of patients to identify health risks and predicting mortality as researchers relied in some cases on analyzing some clinical criteria such as oxygen level, the genome type, and patient's phenotypic comorbidity, or socio-demographic data to predict the course of the disease (259). The use of AI algorithms has also accelerated drug testing. For instance, a deep learning system created by Google DeepMind (Alpha Fold) provided critical information about protein structures related to COVID-19 for use in vaccine discovery, which would take much longer if implemented through traditional experimental methods (251).

Although the COVID-19 pandemic has successfully accelerated the use of AI applications in the health sector by machine and deep learning models, human skills and laboratory or clinical experiments are still critical to ensure the accuracy of the data provided by AI techniques. It is also notable that, while representing a crucial leap in scientific research, results based on data collection may raise ethical concerns, particularly regarding privacy and discrimination.

## Nanotechnology vs. COVID-19

The roll-out of nano-biosensors and vaccines for COVID-19 demonstrates the crucial role of nanotechnology in combating the public health crisis (260, 261).

### Nano-biosensors for COVID-19

Diagnostic testing for COVID-19 is typically done with RT-qPCR, serological, or antigen tests. Using nano-biosensors is a cheaper, faster, and more effective method recently developed that can detect viral infection. Nano-biosensor fabrication might be described simply as the physical and/or chemical immobilization of biological molecules such as proteins, antibodies, nucleic acids, enzymes, and cells on the surface of a transducer. This biological component interacts selectively with the counter antibodies, antigens, or proteins, resulting in various changes (e.g., mechanical, electrochemical, thermal, and optical) measured by the transducer. Surface modification with nanoparticles of different shapes and sizes effectively boosts the detection sensitivity and accuracy (260, 262).

Because of their distinctive optical and plasmonic features, gold nanoparticles have piqued the interest of researchers working on COVID-19 nano-biosensor production (263). A gold nanoparticle-based nano-biosensor based on the aggregation behavior of gold nanoparticles was reported for the colorimetric detection of COVID-19 (264). Another nano-biosensor based on 4-aminothiophenol-functionalized gold nanoparticles was developed for quick COVID-19 diagnosis, relying on the interaction of the COVID-19 antigen with the functionalized gold nanoparticles (265). Magnetic nanoparticles, such as negatively charged zinc ferrite nanoparticles, have been discussed for COVID-19 detection by interaction with viral RNA concentration (266). Furthermore, a lateral flow immunoassay kit based on selenium nanoparticles was introduced as a simple, quick test for detecting the COVID-19 virus based on color change (267). On top of that, carbon nanotubes were used to create a variety of smartphone-based COVID-19-detecting biosensors (268, 269). Nano-biosensors are more sensitive, accurate, faster, dependent, and practical for simple applications compared to existing diagnostic techniques for COVID-19 detection (such as RT-PCR, CT imaging, serological immunoassay, etc.) (260, 270–272).

### Nano-based COVID-19 vaccines

Nanovaccinology proved invaluable in battling the COVID-19 pandemic, supplying the globe with viable vaccine formulations against COVID-19 in an incredible amount of time. Nanotechnology aided in developing nanoparticle platforms for administering molecular vaccines that protect against premature degradation and off-targeting. To obtain stimuli-responsiveness and high immunogenicity, the nanoparticle vaccines might be modified by changing the physicochemical properties, including targeting moieties on their surface and/or co-delivery adjuvants (273–276). Particulate antigen approaches and nano adjuvants are used in numerous cutting-edge vaccinations. Generally, the nano-platforms used for vaccination could be summarized as virus-like particles (VLPs), polymeric nanoparticles (PNPs), inorganic nanoparticles, virosomes, and lipid nanoparticles (LNPs).

VLPs, the most popular platform, comprise the virus surface proteins that are highly organized in symmetrical architectures without the genetic material, leading to safe and productive vaccinations (277, 278). The few studies conducted to develop VLPs containing the structural proteins of SARS-CoV-2, such as the spike protein, have shown tolerability and immunogenicity (279, 280).

PNPs are formed by self-assembly of architecturally structured synthetic polymers such as poly lactide-co-glycolic acid or natural proteins. PNPs are utilized as cargo to sustain vaccine release and/or as adjuvants to activate the immune system (281, 282). In that regard, Ufovax's self-assembling protein nanoparticle (1c-SApNP) vaccine platform technology has been exploited to develop a vaccine against SARS-CoV-2 (283).

Inorganic NPs that use gold and silica are advantageous in terms of the high loading capacity of the antigen, which can encapsulate into the mesoporous structures and is stabilized by hydrophobic or electrostatic forces. However, the toxicity, non-biodegradability, and bioaccumulation of the inorganic nanoparticles are questionable (284, 285). In a brief investigation, gold NPs were explored as a nanocarrier platform and adjuvant for COVID-19 immunization, yet the vaccination improvement was poor. (286). On the other hand, biodegradable mesoporous silica nanoparticles were employed as a nanocarrier for ten different epitopes of the spike (S) glycoprotein of SARS-CoV-2, and the *in vivo* results revealed high activation of particular immune responses (287).

Virosomes are liposomes that are characterized by carrying conjugated antigenic epitopes on their surface. Virosomal vaccines against hepatitis A and influenza viruses are already marketed under the trade names Epaxal^®^ and Inflexal^®^ (288, 289). Despite being able to provide effective vaccination, the trials to create a COVID-19 vaccine candidate based on virosomes were limited (290, 291).

LNPs played a key role in nanovaccinology during the pandemic because they enabled the delivery of the highly effective and affordable COVID-19 vaccine's mRNA (292, 293). LNPs have an added benefit in nanovaccinology because they protect mRNA from phagocyte absorption and endogenous nuclease destruction while also facilitating the diffusion of charged hydrophilic mRNA through hydrophobic cell membranes for the expression of proteins (293, 294). LNPs for mRNA distribution are highly symmetrical, hydrophobic structures made mostly of ionizable cationic lipids, which are employed to circumvent the toxicity disadvantage of regular cationic lipids. The ionizable cationic lipids are distributed primarily in the hydrophilic core, forming internal hydrophobic areas that ionize during acidic manufacturing conditions, allowing electrostatic encapsulation of mRNA. In contrast, these ionizable cationic lipids at physiological pHs become uncharged and thus more biocompatible (293, 295, 296). PEGylated lipids are also employed to cover the core, reducing opsonization and so avoiding uptake by phagocytes, and increasing vaccine circulation within the blood (297, 298). Moderna (mRNA-1273) and Pfizer-BioNTech (BNT162b2) are the two mRNA-based LNP COVID-19 vaccines that have been emergently licensed by the US-FDA to address this crisis (293). Both vaccines are made of ionizable cationic lipid, PEGylated lipid, cholesterol, and distearoylphosphatidylcholine and encapsulate mRNA encoding the SARS-CoV-2 spike protein, which undergoes translation in the cytoplasm into the SARS-CoV-2 spike protein, triggering a reaction by the immune system (273, 299).

## Conclusions and future perspectives

The rapid global response to the COVID-19 pandemic has led to significant advancements in our understanding of the virus and its treatment. Researchers and medical professionals have collaborated to identify effective treatments, develop vaccines, and leverage artificial intelligence and nano-based technologies for diagnostic aid. The evolution of COVID-19, from its etiology to pathogenicity and pathophysiology, has been thoroughly explored in recent literature. Both synthetic and natural treatments have been investigated, and the scientific community remains vigilant in addressing the ongoing challenges posed by SARS-CoV-2. As we continue to learn and adapt, the collective efforts of scientists worldwide will play a crucial role in mitigating the impact of COVID-19 and preventing future outbreaks. The COVID-19 pandemic, caused by the positive-strand RNA virus SARS-CoV-2, has presented significant challenges to global health. The virus's new strains, classified into variants of concern and variants of interest, including Alpha, Beta, Gamma, and Delta variants, have further complicated the situation. The etiology of COVID-19 shows that symptoms may be accentuated with comorbidities like age. Additionally, COVID-19 has been associated with acute respiratory disease, acute kidney injury, cardiovascular damage, and other complications. It exacerbates systemic inflammation, which can be detrimental for those with preexisting conditions like coronary heart disease. The virus may also induce multiorgan damage, repression of the immune system, hypercytokinemia, and more.

Various treatments have been implemented from antiviral therapies, corticosteroid therapies, immunotherapies, dietary supplements, and more. Additionally, artificial intelligence, in conjunction with other technologies, has been used to aid in the fight against the virus. Despite the promise this new technology holds for detecting and monitoring infections, it is only in its developing stages and must be further tested before it is fully implemented with regard to other communicable diseases. In the case of pregnant women, there is evidence of greater rates of miscarriage and hypertension, indicating placental involvement. However, the majority of research has found no evidence that the placenta contains SARS-CoV-2 RNA. The latest research indicates that vertical transmission of SARS-CoV-2 is improbable, but pregnant women may be at a higher risk for more severe consequences. As we continue to battle this pandemic, it is crucial to keep researching and developing new strategies and treatments. The collective efforts of scientists worldwide will play a crucial role in mitigating the impact of COVID-19 and preventing future outbreaks. The dynamic association between humoral and cellular immunity during the battle against SARS-CoV-2 infection is an area of interest for future research. The lessons learned from this pandemic will undoubtedly be valuable in preparing for and managing future health crises.
