# Supplementary material for: Comprehensive review on COVID-19: etiology, pathogenicity, and treatment
Source: Front Med (Lausanne). 2025 Sep 23;12:1569013. doi: 10.3389/fmed.2025.1569013 (PMC12502089; doi:10.3389/fmed.2025.1569013)
Supplement: Supplementary file 1 [file Data_Sheet_1.pdf]

Table S1

n	Cpd	Class	Source	Target	results	Ref
1.	Glycyrrhizin	Terpene	*Glycyrrhiza glabra*	Mpro	**Docking score (Auto Dock Vina)** -8.1 kcal/mol	[1]
					**In vitro against SARS-CoV-2 in Vero E6 cells**<br>**EC_{50}** 0.44 mg/ml<br>**In vitro against Mpro**<br>**At 2000 µM (1.6 mg/mL)**: complete inhibition of Mpro activity<br>**At 30 µM (0.024 mg/mL)**: reduced Mpro activity by 70.3%	[2]
				Mpro<br>PLpro<br>N-protein	**Docking score (AutoDock Vina in PyRx)**<br>Mpro -8.7 kcal/mol<br>PLpro -7.9 kcal/mol<br>N-protein -7.9 kcal/mol<br>**Total Binding Energy**<br>**Mpro**<br>MMGBSA -24.42 kcal/mol<br>MMPBSA -10.80 kcal/mol<br>**PLpro**<br>MMGBSA -48.69 kcal/mol<br>MMPBSA -38.17 kcal/mol<br>**N-protein**<br>MMGBSA -30.05 kcal/mol<br>MMPBSA -25.95 kcal/mol	[3]
				S-protein	**Docking score (Autodock vina)** -9.2 kcal/mol<br>**ΔG (MM-PBSA)** -331.723 **kJ/mol**	[4]
				E-protein	**Docking score (PyRX)** -9.1 kcal/mol	[5]
				RBD of S-protein	**Docking score (AutoDock Vina 1.1.2)**<br>WT -8.5 kcal/mol<br>Delta -8.7 kcal/mol<br>Omicron -8.4 kcal/mol	[6]
2.	Tryptanthrine	Alkaloid	*Strobilanthes cusia*	Mpro	**Docking score (Auto Dock Vina)** -8.2 kcal/mol	[1]
3.	Rhein	Anthraquinone	*Aloe barbadensis*		**Docking score (Auto Dock Vina)** -8.9 kcal/mol	
4.	Berberine	Alkaloid	*Berberis aristata*		**Docking score (Auto Dock Vina)** -8.1 kcal/mol	
5.	Boceprevir	Carboxamide	Synthetic	Mpro	**In vitro against Mpro**<br>**IC_{50}** 8.0±1.5 µM<br>**In vitro against SARS-CoV-2 in Vero cells**<br>**IC_{50}** 15.57 µM	[7]
					**Docking score (GOLD version 5.8)** 64.21<br>**ΔG (MM-GBSA)** -34.7 Kcal/mol	[8]
6.	GC376	Oxopyrrolidin			**In vitro against Mpro**<br>**IC_{50}** 0.15±0.03 µM<br>**In vitro against SARS-CoV-2 in Vero cells**<br>**IC_{50}** 0.70 µM	[7]
7.	Telaprevir	α-ketoamide derivative	Synthetic	Mpro	**Docking score (AutoDockVina)** -5.9 kcal/mol	[9]
8.	Aminoglutethimide	Pyridine derivative			**Docking score (AutoDockVina)** -5.6 kcal/mol	
9.	Apixaban				**Docking score (AutoDockVina)** -7.4 kcal/mol	
10.	Buspirone				**Docking score (AutoDockVina)** -6.9 kcal/mol	
11.	Lenalidomide				**Docking score (AutoDockVina)** -6.5 kcal/mol	

12.	Pomalidomide				**Docking score (AutoDockVina)** -6.6 kcal/mol	
13.	Tert-butyl (1-((S)-1-(((S)-4-(benzylamino)-3,4-dioxo-1-((S)-2-oxopyrrolidin-3-yl)-butan-2-yl)amino)-3-cyclopropyl-1-oxopropan-2-yl)-2-oxo-1,2-dihydropyridin-3-yl)carbamate	Pyrrolidin derivative	Synthetic	Mpro	**In vitro against Mpro**<br>**IC_{50}** 0.67 ±0.18 μM	[10]
14.	Ebselen	Benzoselenazole	Synthetic	Mpro	**In vitro against Mpro**<br>**IC_{50}** 0.67 ± 0.09 μM<br>**In vitro against SARS-CoV-2 in Vero E6 cells**<br>**EC_{50}** 4.67 ± 0.80 μM	[11]
15.	Quercetin	Flavonoid	*Citrus fruits*	Mpro	**In vitro against Mpro**<br>**K_i** 7.4 μM	[12]
				RdRp<br>Mpro	**Docking score (AutoDock Vina)**<br>RdRp −6.1 kcal/mol<br>Mpro −7.4 kcal/mol	[13]
				Mpro<br>Nsp15	**Docking score (AutoDock4 Tool)**<br>Mpro -6.58 Kcal/mol<br>Nsp15 -6.49 Kcal/mol	[14]
				RdRp<br>Mpro	**Docking score (MOE 2019.01)**<br>RdRp -7.2 Kcal/mol<br>Mpro -8.5 Kcal/mol	[15]
			Forsythia suspensa	Mpro	**In vitro against Mpro**<br>**IC_{50}** 26.15 μM	[16]
16.	Baicalin	Flavonoid	*Scutellaria amoena*	Mpro	**Docking score (the Schrodinger software suite (Maestro, version 11.8.012)(Glide score)** −8.776<br>**In vitro against Mpro**<br>**IC_{50}** 34.71 μM	[17]
				Nsp16	**Docking score (Autodock Vina tool compiled in the PyRx 0.8)** -8.7 kcal/mol	[18]
17.	Herbacetin		*Ripariosida hermaphrodita*	Mpro	**Docking score (the Schrodinger software suite (Maestro, version 11.8.012) (Glide score)** -8.738<br>**In vitro against Mpro**<br>**IC_{50}** 53.90 μM	[17]
18.	Pectolinarin		*Kickxia elatine*		**Docking score (the Schrodinger software suite (Maestro, version 11.8.012) (Glide score)** −10.969<br>**In vitro against Mpro**<br>**IC_{50}** 51.64 μM	
19.	Bonducellpin D	Terpene	*Guilandina bonduc*	Mpro	**Docking score (AutoDock4.2 software)** −9.28 kcal/mol	[19]
20.	TriS-heparin	Polysaccharide	Synthetic	S-protein	**In vitro against SARS-CoV-2 (NCCP43326) in VERO cells**<br>**EC_{50}** 5 μM	[20]
21.	Heparin				**In vitro against SARS-CoV-2 (NCCP43326) in VERO cells**<br>**EC_{50}** 2.1 μM	
22.	RPI-27		*Saccharina japonica*		**In vitro against SARS-CoV-2 (NCCP43326) in VERO cells**<br>**EC_{50}** 0.08 μM	
23.	Remdesivir	Adenosine analogue	Synthetic	RdRp	**In vitro against nCoV-2019 BetaCoV/Wuhan/WIV04/2019 in Vero E6 cells**<br>**EC_{50}** 0.77 μM	[21]
					**In vitro against SARS-CoV-2 in Vero E6**<br>**EC_{50}** 23.15 μM	[22]
					**In vitro against**<br>**EC_{50}**<br>**Original virus (SARS-CoV-2/human/Denmark/DK-AHH1 /2020) in**<br>**Vero E6** 1.5 μM	[23]

					**Huh7.5** 0.03 μM<br>**Adapted virus in**<br>**Vero E6** 2.9 μM<br>**Huh7.5** 0.05 μM<br>**Calu-1** 0.29<br>**A549** 0.31	
					**In vitro against SARS-CoV-2 in VeroE6-eGFP cells**<br>**EC_{50}** 5.4 μM<br>**Caco-2 cells**<br>**EC_{50}** 1.3 μM	[24]
24.	5,7,3′,4′-Tetrahydroxy-2’-(3,3-dimethylallyl) isoflavone	Flavonoid	*Psorothamnus arborescens*	Mpro	**Docking score (MOE)** −16.35 kcal/mol	[25]
25.	Myricitrin	Flavonoid	*Myrica cerifera*		**Docking score (MOE)** −15.64 kcal/mol	
		Flavonoid	*Myrica esculenta*	Mpro<br>RdRp<br>ACE2	**Docking score (Autodock vina)**<br>Mpro -8.9 kcal/mol<br>RdRP -7.9 kcal/mol<br>ACE2 -7.5 kcal/mol	[26]
26.	Methyl rosmarinate	Hydroxycinnamic acid	*Hyptis atrorubens Poit*	Mpro	**Docking score (MOE)** −15.44 kcal/mol	[25]
27.	3,5,7,3′,4′,5′-hexahydroxy flavanone -3-O-beta-D-glucopyranoside	Flavonoid	*Phaseolus vulgaris*		**Docking score (MOE)** −14.42 kcal/mol	
28.	(2S)-Eriodictyol 7-O-(6″-O-galloyl)-beta-D-glucopyranoside	Flavonoid	*Phyllanthus emblica*		**Docking score (MOE)** −14.41 kcal/mol	
29.	Calceolarioside B	Hydroxycinnamic acid	*Fraxinus sieboldiana*		**Docking score (MOE)** −14.36 kcal/mol	
30.	Myricetin 3-O-beta-D glucopyranoside	Flavonoid	*Camellia sinensis*		**Docking score (MOE)** −13.70 kcal/mol	
31.	Licoleafol	Flavonoid	*Glycyrrhiza uralensis*		**Docking score (MOE)** −13.63 kcal/mol	
32.	Amaranthin	Pyridine	*Amaranthus tricolor*		**Docking score (MOE)** −12.67 kcal/mol	
33.	Atazanavir	Peptide	Synthetic	Mpro<br>RdRp<br>Helicase<br>EndoRNAse	**K_d =**<br>Mpro 94.94 nM<br>RdRP 21.83 nM<br>Helicase 25.92 nM<br>EndoRNAse 50.32 nM	[27]
34.	Lopinavir	Dicarboxylic acid diamide	Synthetic	Mpro	**In vitro against SARS-CoV-2 in Vero E6**<br>**EC_{50}** 26.63 μM	[22]
					**Docking score (PyRX/AutoDock Vina)** −8.0 kcal/mol<br>**In vitro against SARS-CoV-2 in Vero-E6 cells**<br>**EC_{50}** 30.1 μM	[28]
35.	Homorringtonine	Alkaloid	*Cephalotoxus fortune*	-	**In vitro against SARS-CoV-2 in Vero E6**<br>**EC_{50}** 2.55 μM	[22]
36.	Emetine	Alkaloid	*Psychotria ipecacuanha*		**In vitro against SARS-CoV-2 in Vero E6**<br>**EC_{50}** 0.46 μM	
37.	Remdesivir and Emetine in combination	-	-	-	**% Inhibition**<br>Remdesivir (6.25 μM) and emetine (0.195 μM) = 64.9%	
38.	Thimerosal	Organomercurial compound	Synthetic	Mpro	**In vitro against Mpro**<br>**IC_{50}** 0.6 ± 0.1 μM	[29]
39.	Phenylmercuric acetate	Arylmercury compound	Synthetic		**In vitro against Mpro**<br>**IC_{50}** 0.4 ± 0.06 μM	
40.	Bronopol	Nitrogenous	Synthetic		**In vitro against Mpro**<br>**IC_{50}** 4.4 ± 0.6 μM	
41.	Tannic acid	polyphenol	*Caesalpinia spinosa*		**In vitro against Mpro**<br>**IC_{50}** 2.1 ± 0.2 μM	

42.	Hematoporphyrin	Dicarboxylic acid	Synthetic		**In vitro against Mpro**<br>**IC_{50}** 3.9 ± 0.6 μM	
43.	3,4-didesmethyl-5-deshydroxy-3'-ethoxyscleroin	Benzophenone	Synthetic		**In vitro against Mpro**<br>**IC_{50}** 10.6 ± 1.3 μM	
44.	Evans blue	Azo dye	Synthetic		**In vitro against Mpro**<br>**IC_{50}** 0.2 ± 0.06 μM	
45.	Chicago Sky Blue	Disazo dye	Synthetic		**In vitro against Mpro**<br>**IC_{50}** 7.7 ± 1.6 μM	
46.	Alteramide A	Alkaloid	*Pseudoalteromonas* sp.	RdRp	**Docking score (Autodock Vina)** -9.0 kcal/mol	[30]
47.	Isotirandamycin B	Tirandamycins	*Streptomyces sp.*	Nsp16	**Docking score (Autodock Vina)** -8.4 kcal/mol	
48.	Tirandamycin A				**Docking score (Autodock Vina)** -8.5 kcal/mol	
49.	Tirandamycin B				**Docking score (Autodock Vina)** -8.3 kcal/mol	
50.	Eravacycline	Tetracycline	Synthetic	Mpro	**Docking score (Glide)** −8.8 kcal/mol<br>**ΔG(MMPBSA-WSAS)** −7.7 ± 0.5 kcal/mol	[31]
51.	Streptomycin	Aminoglycoside	*Streptomyces griseus*		**Docking score (Glide)** −6.9 kcal/mol<br>**ΔG(MMPBSA-WSAS)** −3.8 kcal/mol	
52.	TK 2.7.0	Oxopyrrolidin	Synthetic		**Docking score (Glide)** −8.8 kcal/mol<br>**ΔG(MMPBSA-WSAS)** −12.9 kcal/mol	
53.	N-(3-cyclohexyl-1-((1-hydroxy-1-(methylthio)-3-(2-oxopyrrolidin-3-yl)propan-2-yl)amino)-1-oxopropan-2-yl)-1H-indole-2-carboxamide	Pyrrolidin	Synthetic	Mpro	**In vitro against Mpro**<br>**IC_{50}** 0.053 ± 0.005 μM<br>**In vitro against SARSCoV-2 in Vero E6 cells**<br>**EC_{50}** 0.53 ± 0.01 μM	[32]
54.	N′,N″-[oxybis(4,1-phenylenecarbonyl)]bis(3-methoxybenzohydrazide)<br>**(7781334)**	Hydrazide	Synthetic	ACE2	**Docking results (MOE)** −5.87 ± 0.49 kcal/mol	[33]
55.	2,2′-{1,4-butanediylbis[(4-ethyl-4H-1,2,4-triazole-5,3-diyl)thio]}bis(1-phenylethanone)<br>**(7676800)**	Triazole			**Docking results (MOE)** −5.84 ± 0.60 kcal/mol	
56.	2,2′-{1,4-butanediylbis[(4-ethyl-4H-1,2,4-triazole-5,3-diyl)thio]}bis(1-phenylethanone)<br>**(7956590)**	Triazole			**Docking results (MOE)**−5.83 ± 0.55 kcal/mol	
57.	N,N′-[methylenebis(2-hydroxy-4,1-phenylene)]bis[2-(3,4-dimethoxyphenyl)acetamide]<br>**(7782787)**	Acetamide			**Docking results (MOE)** −5.77 ± 0.36 kcal/mol	
58.	Ethyl 4-({[(4-allyl-5-{2-[(2,4-dimethylphenyl)amino]-2-oxoethyl}-4H-	Triazole			**Docking results (MOE)** −5.69 ± 0.55 kcal/mol	

	1,2,4-triazol-3-yl)thio]acetyl}amino)benzoate **(7938481)**					
59.	2-(4-methoxyphenyl)-N-{[4-methyl-5-({2-[(5-methyl-4-phenyl-1,3-thiazol-2-yl)amino]-2-oxoethyl}thio)-4H-1,2,4-triazol-3-yl]methyl}acetamide **(7517329)**	Acetamide			**Docking results (MOE)** −5.69 ± 0.48 kcal/mol	
60.	N,N′-1,6-hexanediylbis[2-(4-isopropylphenoxy)acetamide] **(7787375)**	Acetamide			**Docking results (MOE)** −5.65 ± 0.55 kcal/mol	
61.	N′,N″-[oxybis(4,1-phenylenecarbonyl)]bis(2-chlorobenzohydrazide) **(7783270)**	Hydrazide			**Docking results (MOE)** −5.62 ± 0.48 kcal/mol	
62.	7134636, N-(5-{[2-(dibenzo[b,d]furan-3-ylamino)-2-oxoethyl]thio}-1,3,4-thiadiazol-2-yl)-2-methoxybenzamide **(7134636)**	Benzamide			**Docking results (MOE)** −5.56 ± 0.48 kcal/mol	
63.	N,N′-1,2-phenylenebis[2-(4-ethylphenoxy)acetamide] **(7390655)**	Acetamide			**Docking results (MOE)** −5.54 ± 0.57 kcal/mol	
64.	Ethyl 3-({[(5-{[(3,4-dimethoxybenzoyl)amino]methyl}-4-methyl-4H-1,2,4-triazol-3-yl)thio]acetyl}amino)benzoate **(7652337)**	Triazole			**Docking results (MOE)** −5.54 ± 0.38 kcal/mol	
65.	N,N′-(oxydi-4,1-phenylene)bis[2-(2-methoxyphenoxy)acetamide] **(6898502)**	Acetamide			**Docking results (MOE)** −5.53 ± 0.53 kcal/mol	
66.	N,N′-4,4′-biphenyldiylbis[2-(2-methoxyphenoxy)acetamide] **(6936307)**	Acetamide			**Docking results (MOE)** −5.53 ± 0.56 kcal/mol	
67.	1-[(3,4-dimethoxyphenyl)acetyl]-N,N′-bis(2-thienylmethyl)-1H-1,2,4-triazole-3,5-diamine **(7974985)**	Triazole			**Docking results (MOE)** −5.53 ± 0.55 kcal/mol	

68.	N,N′-1,2-propanediylbis[2-(4-tert-butylphenoxy)acetamide] **(7153800)**	Acetamide			**Docking results (MOE)** −5.52 ± 0.65 kcal/mol	
69.	4,4′-oxybis[N-(2-ethoxyphenyl)benzamide] **(7588589)**	Benzamide			**Docking results (MOE)** −5.51 ± 0.55 kcal/mol	
70.	2-chloro-N-{2-[5-({2-[(3-cyano-4,5,6,7-tetrahydro-1-benzothien-2-yl) amino]-2-oxoethyl}thio)-4-ethyl-4H-1,2,4-triazol-3-yl]ethyl}benzamide **(7844832)**	Benzamide			**Docking results (MOE)** −5.51 ± 0.47 kcal/mol	
71.	N-{2-[4-allyl-5-({2-oxo-2-[(4-phenyl-1,3-thiazol-2-yl)amino]ethyl} thio)-4H-1,2,4-triazol-3-yl]ethyl}-4-methylbenzamide **(7845662)**	Benzamide			**Docking results (MOE)** −5.51 ± 0.47 kcal/mol	
72.	N-[2-(3,4-dimethoxyphenyl)ethyl]-2-{4-[(isobutylamino)sulfonyl]-2-methylphenoxy}acetamide **(7987131)**	Acetamide			**Docking results (MOE)** −5.51 ± 0.50 kcal/mol	
73.	N~2~-[4-(benzyloxy)phenyl]-N~1~-(4-{[(2,6-dimethylphenyl) amino]sulfonyl}phenyl)-N~2~-(methylsulfonyl)glycinamide **(7939484)**	Glycinamide			**Docking results (MOE)** −5.50 ± 0.57 kcal/mol	
74.	Rutin	Flavonoid	*Tragia involerta*	Mpro	**Docking score (iGEMDOCK)** -133.06 kcal/ mol	[34]
					**Docking score (MTi AutoDock)** −15.63 kcal/mol<br>**ΔG (MM/GBSA)** −70.41±4.81 kcal/mol	[35]
					**Docking score (GOLD version 5.8)** 74.55<br>**ΔG (MM-GBSA)** -43.0 Kcal/mol	[8]
				E-protein	**Docking score (PyRx)** −9.3 kcal/mol	[36]
75.	Luteolin 7-rutinoside	*Polyketide*	*Hygrophila auricualata*	Mpro	**Docking score (iGEMDOCK)** −134.6 kcal/ mol	[34]
76.	Acetoside	Glycoside	*Clerodendrum serratum*		**Docking score (iGEMDOCK)** −153.06 kcal/ mol	
77.	Chebulagic acid	*Tannin*	*Terminalia chebula*		**Docking score (iGEMDOCK)** −124.3 kcal/ mol	
78.	Syrigaresinol	Lignan	*Sausurea lappa*		**Docking score (iGEMDOCK)** −120.03 kcal/ mol	
79.	Acanthoside B	Glycoside	*Sida acuta*		**Docking score (iGEMDOCK)** -122.21 kcal/ mol	
80.	Violanthin	Flavonoid	*Adhatoda vasica*		**Docking score (iGEMDOCK)** −114.9 kcal/ mol	

81.	Andrographidine C	Flavonoid	*Andrographis paniculate*	Mpro	**Docking score (iGEMDOCK)** −101.8 kcal/ mol	
				RdRp	**Docking score (Glide v7.8)** -8.12 kcal/mol	[37]
82.	Myricetin	Flavonoid	*Syzygium aromaticum*	Mpro	**Docking score (iGEMDOCK)** −99.96 kcal/ mol	[34]
					**Docking score (Glide)** −8.473<br>**In vitro against Mpro**<br>**IC_{50}** 3.684 ± 0.076 μM	[38]
				TMPRSS2	**Docking score (Autodock Vina)** −8.3 kcal/mol	[39]
				Mpro	**Docking score (Glide)** -8.364<br>**In vitro against Mpro**<br>**IC_{50}** 2.86 μM	[40]
				Mpro<br>Nsp15	**Docking score (AutoDock4 Tool)**<br>Mpro -6.15 Kcal/mol<br>Nsp15 -6.52 Kcal/mol	[14]
83.	Gingerenone -A	Phenolic	*Zingiber Officianle*	Mpro	**Docking score (iGEMDOCK)** −93.9 kcal/ mol	[34]
84.	Tinosporinone	Furan derivative	*Tinospora cordifolia*		**Docking score (iGEMDOCK)** −83.42 kcal/ mol	
85.	Geraniol	Terpene	*Ceolus ambonicus*		**Docking score (iGEMDOCK)** −62.87 kcal/ mol	
86.	Nootkatone	Terpene	*Cypreus rotundus*		**Docking score (iGEMDOCK)** −62.4 kcal/ mol	
87.	Asarianin	Sterol	*Piper longum*		**Docking score (iGEMDOCK)** −79.94 kcal/ mol	
88.	Gamma Sitosterol	*Sterol*	*Anacyclus pyrethrum*		**Docking score (iGEMDOCK)** −81.94 kcal/ mol	
89.	((2E,6E)-2-[(3-hydroxyphenyl)methylidene]-6-[(3-methoxyphenyl)methylidene]cyclohexan-1-one)<br>**(ZINC07333416)**	Phenolic	Synthetic	Mpro	**Docking score (AutoDock 4.2)** - 8.72 kcal/mol	[41]
90.	[Pd(ONS-SVSL3-OCH3)(PPh3)]	Palladium(II) Complex	Synthetic	Mpro	**Docking score (Autodock 4.2)** −9.38 kcal/mol	[42]
91.	Caffeic acid	Hydroxycinnamic acid	*Vitis amurensis*	M-protein	**Docking score (PyRx)** −8.4 kcal/mol	[36]
				Mpro	**Docking score (MOE 2019.01)** -5.7 Kcal/mol	[15]
92.	Grazoprevir	Quinoxaline derivative	Synthetic	N-protein	**Docking score (PyRx)** −8.5 kcal/mol	[36]
93.	Bisdesethylchloroquine	4-aminoquinoline	Synthetic	ACE2	**Docking score (AutoDock Vina)** −6.1 kcal/mol	[43]
94.	N-(1,3-dihydroxy-2-(hydroxymethyl)propan-2-yl)-7,8-dihydroxy-2-oxo-2H-chromene-3-carboxamide	Coumarin	Synthetic	Mpro	**Docking score (AutoDock Vina)** –6.6 kcal/mol<br>**ΔG (MM/GBSA)** −24.8 ± 0.12 kcal/mol	[44]
95.	7-hydroxy-4-((4-(2-hydroxyethyl)piperazin-1-yl)methyl)-2H-chromen-2-one				**Docking score (AutoDock Vina)** –6.7 kcal/mol<br>**ΔG (MM/GBSA)** −24.7 ± 0.07 kcal/mol	
96.	N-(1,3-dihydroxy-2-(hydroxymethyl)propan-2-yl)-11-oxo-2,3,6,7-tetrahydro-1H,5H,11H-pyrano[2,3-				**Docking score (AutoDock Vina)** –7.1 kcal/mol<br>**ΔG (MM/GBSA)** −22.8 ± 0.10 kcal/mol	

	f]pyrido[3,2,1-ij]quinoline-10-carboxamide					
97.	7-hydroxy-4-(piperidin-1-ylmethyl)-2H-chromen-2-one				**Docking score (AutoDock Vina)** –6.6 kcal/mol<br>**ΔG (MM/GBSA)** −22.6 ± 0.08 kcal/mol	
98.	7-hydroxy-4-(morpholinomethyl)-2H-chromen-2-one				**Docking score (AutoDock Vina)** –6.6 kcal/mol<br>**ΔG (MM/GBSA)** −20.5 ± 0.11 kcal/mol	
99.	5-(morpholinomethyl)quinolin-8-ol	Quinoline derivative			**Docking score (AutoDock Vina)** –6.5 kcal/mol<br>**ΔG (MM/GBSA)** −20.5 ± 0.11 kcal/mol	
100.	P-coumaric acid	Hydroxycinnamic acid	*Apis mellifera*	RdRp<br>Mpro	**Docking score (AutoDock Vina)**<br>**RdRp** −5.3 kcal/mol<br>**Mpro** −5.6 kcal/mol	[13]
101.	Kaempferol	*Flavonoid*			**Docking score (AutoDock Vina)**<br>**RdRp** −6.2 kcal/mol<br>**Mpro** −7.8 kcal/mol	
102.	(1R,2R)-2-N-(7-Chloroquinolin-4-yl)cyclohexane-1,2-diamine **(ZINC38050615)**	Quinolin	Synthetic	S-protein	**Docking score (AutoDockTools-1.5.6**) -7.29 kcal/mol	[45]
103.	Inophyllum A	Coumarin	*Calophyllum inophyllum*	Mpro	**Docking score (Autodock vina1.1.2)** -8.4 kcal/mol	[46]
104.	3-hydrazono-2-(3-phenyl-2,3,3a,4,5,11-hexahydrochromeno [2,3-g]indazol-11-yl) cy clohex-1-enol		Synthetic		**Docking score (Autodock vina1.1.2** -7.9 kcal/mol	
105.	4-(11-(6-hydrazono-2-hydroxycyclohex-1-en-1-yl)-2,3,3a,4,5,11-hexahy drochro meno[2,3-g]indazol-3-yl)phenol		Synthetic		**Docking score (Autodock vina1.1.2)** -7.9 kcal/mol	
106.	4-(6-(4-(acridin-9-ylamino)phenyl)-2-amino-2H-1,3-oxazin-4-yl)-2-methoxyphenol	Oxazine	Synthetic	Mpro	**Docking score (Glide)** -7.829<br>**ΔG (Prime/MM-GBSA approach)** -57.1818 kcal/mol	[47]
107.	3-(6-(4-(acridin-9-ylamino)phenyl)-2-amino-2H-1,3-oxazin-4-yl)phenol				**Docking score (Glide)** -6.78<br>**ΔG (Prime/MM-GBSA approach)** -54.6839 kcal/mol	
108.	6-(4-(acridin-9-ylamino)phenyl)-4-(2,5-dimethoxyphenyl)-2H-1,3-oxazin-2-amine				**Docking score (Glide)** -6.65<br>**ΔG (Prime/MM-GBSA approach)** -54.4053 kcal/mol	
109.	4-(6-(4-(acridin-9-ylamino)phenyl)-2-amino-2H-1,3-oxazin-4-yl)benzene-1,3-diol				**Docking score (Glide)** -6.37<br>**ΔG (Prime/MM-GBSA approach)** 53.7761 kcal/mol	
110.	6-(4-(acridin-9-ylamino)phenyl)-4-(2-methoxyphenyl)-2H-1,3-oxazin-2-amine				**Docking score (Glide)** -6.12<br>**ΔG (Prime/MM-GBSA approach)** -59.0951 kcal/mol	

111.	Conivaptan	Benzazepine derivative	Synthetic	ExoN domain of Nsp14	**Docking score (AutoDock VINA)** -8.6 kcal/mol<br>**ΔG (MM-GBSA)** -85.86 ± 0.68 kcal/mol	[48]
112.	Hesperidin	*Disaccharide*	*Citrus fruits*		**Docking score (AutoDock VINA) -**8.6 kcal/mol<br>**ΔG (MM-GBSA) -**119.07 ± 0.69 kcal/mol	
				Mpro<br>RdRp<br>ACE2	**Docking score (Autodock vina)**<br>MPro -8.5 kcal/mol<br>RdRP -8.7 kcal/mol<br>ACE2 -9.8 kcal/mol	[26]
113.	2-(2-formylphenoxy)-N-(thiazol-2-yl)acetamide *(***HL Enol/form**)	Thiazole	Synthetic	Mpro<br>RBD S-protein	**Docking score (MOE ver. 2018)**<br>**Mpro** -5.9171 Kcal/mol<br>**RBD S-protein** -5.1185 Kcal/mol	[49]
114.	1-[(2R,3R,2S,5R)-3,4-dihydroxy-5-(hydroxymethyl)oxolan-2-yl]-5-ethynyl-1,2,3,4-tetrahydropyrimidine-2,4-dione (analogous of Idoxuridine)	Nucleoside analog			**Docking score (MOE ver. 2018)**<br>**Mpro** -6.1962 Kcal/mol<br>**RBD S-protein** -5.1074 Kcal/mol	
115.	Withanoside-IV	Phenanthrene	*Withania somnifera*	TMPRSS	**Docking score (Glide)** -6.92 kcal/mol<br>**ΔG (MM/GBSA)** - 42.80 ± 7.46 kcal/ mol<br><br>**%inhibition after 48h at 10 µM**<br>**E (envelope)** 45.03%<br>**N (nucleocapsid)** 44.79%	[50]
116.	Hypericin	<u>Naphthodianthrone</u>	*Hypericum perforatum*	Mpro	**Docking score (Autodock Vina)** -11 kcal/mol	[51]
117.	Isohypericin				**Docking score (Autodock Vina)** -11 kcal/mol	
118.	Isoorientin	Flavonoid	<u>*Carex fraseriana*</u>	IL-6	**Docking Score (AutoDock Vina)** -7.7 kcal/mol<br>**ΔG (MMGBSA)**−28.4598 kcal/mol	[52]
119.	Lupeol	Terpene	<u>*Ficus septica*</u>		**Docking Score (AutoDock Vina)** -7.4 kcal/mol<br>**MMGBSA** −19.2682 kcal/mol	
120.	Bislatumlide A	Terpene	*Sarcophyton latum*	Mpro	**Docking Score (AutoDock4.2.6)** –9.6 kcal/mol<br>**ΔG (MM-GBSA)** -44.8 kcal/mol	[53]
121.	Eucalyptol	Essential oil	*Eucalyptus globulus*	Mpro	**Docking score (AutoDock 4.2 version)** –5.86 Kcal/mol.	[54]
122.	Cyanin	Anthocyanin	*Zingiber officinale*	Mpro<br>RdRp	**Docking score (PyRx 0.8 containing AutoDock Vina)**<br>Mpro −7.7 kcal/mol<br>RdRp −7.9 kcal/mol	[55]
123.	Amentoflavone	Flavonoid	*Mangifera indica*	RdRp<br>Nsp9<br>Nsp3<br>Nsp10-Nsp16<br>S-protein	**Docking score (PyRx 0.8 containing AutoDock Vina)**<br>RdRp −9.3 kcal/mol<br>NSP9 −8.3 kcal/mol<br>NSP3 −7.4 kcal/mol<br>NSP10-NSP16 −8.5 kcal/mol<br>S-protein −8.2 kcal/mol	
				Nsp16	**Docking score (Autodock Vina tool compiled in the PyRx 0.8)** -9.3 kcal/mol	[18]
124.	Agathisflavone	Flavonoid	*Anacardium occidentale*	Mpro<br>RdRp<br>NSP3<br>S-protein	**Docking score (PyRx 0.8 containing AutoDock Vina)**<br>Mpro −8.2 kcal/mol<br>RdRp −8.9 kcal/mol<br>NSP3 −6.6 kcal/mol<br>S-protein −8.2 kcal/mol	[55]
125.	Catechin-7-o-gallate	Phenolic	*Camellia sinensis*	Nsp15<br>Nsp3<br>S-protein	**Docking score (PyRx 0.8 containing AutoDock Vina)**<br>NSP15 −8.5 kcal/mol<br>NSP3 −6.6 kcal/mol<br>S protein −7.3 kcal/mol<br>RdRp −7.9 kcal/mol	

126.	Chlorogenin	Terpene	*Solanum torvum*	Mpro<br>NSP9<br>NSP10-NSP16	**Docking score (PyRx 0.8 containing AutoDock Vina)**<br>Mpro −7.7 kcal/mol<br>NSP9 −8.2 kcal/mol<br>NSP10-NSP16 −7.6 kcal/mol	
127.	Selinexor	Triazole	Synthetic	XPO1 inhibitor	**in vitro against SARS-CoV-2 (2019-nCoV/USA-WA1/2020) in Vero E6 cells**<br>**EC_{50}** 10 nM<br>**EC_{90}** 100 nM<br>therapeutic index (TI) = 35<br><br>**In vitro in Vero E6 cells**<br>selinexor inhibited viral infection when given prophylactically, at the time of viral infection and also up to 24 h following cell infection with IC90 < 10 nM<br><br>**In vivo using Ferrets (*Mustela putorius furo*)** treated with selinexor (5 mg/kg) twice daily for 3 days<br>mean viral RNA of 3.8 log10 viral copies/ gram of lung tissue ($p = 0.0335$)	[56]
128.	Withanoside V	Steroid	*Withania somnifera*	Mpro	**Docking score (Glide)** -8.96 Kcal/mol<br>**ΔG (MM-GBSA)** -87.01 ± 5.01 Kcal/mol	[57]
					**Docking score (AutoDock Vina-based YASARA)**<br>10.32 kcal/mol	[58]
				TMPRSS<br>Mpro	**Docking score (Glide)**<br>**TMPRSS**- 7.96 kcal/mol<br>**Mpro** -10.10 kcal/mol<br>**ΔG (MM/GBSA)**<br>**TMPRSS** -36.19 ± 7.83 kcal/ mol<br>**Mpro**- -35.70 ± 5.17 kcal/ mol	[50]
129.	3-(dimethylsulfamoyl)-N-[3-(furan-2-ylmethylsulfamoyl)phenyl]benzamide	Benzamide	Synthetic	ACE2	**Docking score (CDOCKER)** 63.66<br>**ΔG (MMPBSA)** −63.002 ± 27.25 kJ/mol	[59]
130.	5-(N-ethyl-N-methylsulfamoyl)-N-(1-((4-(6-hydroxypyridin-2-yl)-2-methylbutyl)amino)-3-methyl-1-oxobutan-2-yl)-2-(pyridin-4-yl)benzamide		Synthetic	MPro	**Docking score (CDOCKER)** 50.2016<br>**ΔG (MMPBSA)** −159.83 ± 96.42 kJ/mol	
131.	Ellagic acid	Tannin	*Prunica granatum*	Mpro	**Docking score (AutoDockVina)** - 8.4 kcal/mol	[60]
				RdRp<br>Mpro	**Docking score** (AutoDock Vina)<br>**RdRp** −6.4 kcal/mol<br>**Mpro** −7.5 kcal/mol	[13]
132.	Arjunic Acid	Saponin	*Terminalia arjuna*	Mpro	**Docking score (AutoDockVina)** - 8.1 kcal/mol	[60]
133.	Theasapogenol B		*Camellia sasanqua Aesculus*		**Docking score (AutoDockVina)** - 8.1 kcal/mol	
134.	Euscaphic Acid		*Folium Eriobotryae*		**Docking score (AutoDockVina)** - 8.0 kcal/mol	
135	E)-4-[2-(2-Oxoindolin-3-	Oxoindole	Synthetic	**-**	**In vitro (hCoV-19/Egypt/NRC-03/2020)**<br>**IC_{50}** 3.799 μM	[61]

	ylidene)acetyl]phenyl methanesulfonate					
136	(E)-N-(Ethylsulfonyl)-N-{4-[2-(2-oxoindolin-3-ylidene)acetyl] phenyl}ethanesulfonamide				**In vitro (hCoV-19/Egypt/NRC-03/2020)**<br>**IC_{50}** 3.417 μM	
137.	Epigallocatechin gallate (EGCG)	Phenolic	*Camellia sinensis*	Mpro	**Docking score (AutoDock Vina)** -7.6 kcal/mol<br>**ΔG (MM-GBSA)** -48.92 kcal/mol	[62]
138.	Epicatechingallate (ECG)				**Docking score (AutoDock Vina)** -8.2 kcal/mol<br>**ΔG (MM-GBSA)** -43.56 kcal/mol	
139.	Gallocatechin-3-gallate (GCG)				**Docking score (AutoDock Vina)** -9.0 kcal/mol<br>**ΔG (MM-GBSA)** -53.54 kcal/mol	
140.	Sinapic acid	Phenolic	*Brassica oleracea*	E-protein<br>ADP ribose phosphatase	**Docking score (AutoDock Vina)**<br>E-protein -8.9 kcal/mol<br>ADP ribose phosphatase -7.1 kcal/mol<br>**ΔG (FEP)**<br>E-protein -8.4 kcal/mol<br>ADP ribose phosphatase -9.3 kcal/mol<br>**In vitro against hCoV-19/Egypt/NRC-3/2020 in Vero-E6**<br>**IC_{50}** 2.69 μg/mL	[63]
141.	Pentamidine	Guanidinobenzoyl- or Aminidinobenzoyl-Containing Drugs	Synthetic	TMPRSS2	**Docking score (LeDock)** -8.73 kcal/mol<br>**ΔG (MMGBSA)** -34.86 ± 3.96 kcal/mol	[64]
142.	Hexamidine				**Docking score (LeDock)** -8.47 kcal/mol<br>**ΔG (MMGBSA)** -30.86 ± 3.38 kcal/mol	
143.	Anatibant				**Docking score (LeDock)** -8.60 kcal/mol<br>**ΔG (MMGBSA)** -40.77 ± 4.06 kcal/mol	
144.	WX-UK1				**Docking score (LeDock)** -8.57 kcal/mol<br>**ΔG (MMGBSA)** -39.23 ± 4.77 kcal/mol	
145.	Fradafiban				**Docking score (LeDock)** -7.32 kcal/mol<br>**ΔG (MMGBSA)** -26.20 ± 5.19 kcal/mol	
146.	Otamixaban				**Docking score (LeDock)** -8.50 kcal/mol<br>**ΔG (MMGBSA)** -35.30±2.95 kcal/mol	
147.	Avoralstat				**Docking score (LeDock)** -9.35 kcal/mol<br>**ΔG (MMGBSA)** -36.71 ± 3.97 kcal/mol	
148.	PCI-27483				**Docking score (LeDock)** -9.44 kcal/mol<br>**ΔG (MMGBSA)** -29.82 ± 6.29 kcal/mol	
149.	Propamidine				**Docking score (LeDock)** -7.93 kcal/mol<br>**ΔG (MMGBSA)** -32.49 ± 2.77 kcal/mol	
150.	Dabigatran				**Docking score (LeDock)** -9.51 kcal/mol<br>**ΔG (MMGBSA)** -30.65 ± 3.61 kcal/mol	
151.	Hydroxystilbamidine				**Docking score (LeDock)** -7.56 kcal/mol<br>**ΔG (MMGBSA)** -14.92 ± 3.54 kcal/mol	
152.	Camostat				**Docking score (LeDock)** -8.02 kcal/mol<br>**ΔG (MMGBSA)** -30.42 ± 3.02 kcal/mol	
153.	Citicoline	Cytidine	Natural	Mpro	**Docking results (AutoDock Vina tool)** -7.0 ±0.19 kcal/mol<br>**ΔG (MMGBSA)** -25.53 kcal/mol	[65]
154.	Uridine triacetate	Pyrimidine nucleotide	Synthetic		**Docking score (AutoDock Vina tool)** -6.2 ±0.26 kcal/mol<br>**ΔG (MMGBSA)** -7.07 kcal/mol	
155.	6-O-b-Dglucopyranosyl-5-hydroxyangelicin	Coumarin	*Lawsonia inermis*	Mpro	**Docking score (AutoDock Vina)** -8.7 kcal/mol	[66]
156.	Bavacoumestan A	Sterol	*Lawsonia inermis*		**Docking score (AutoDock Vina)** -8.7 kcal/mol	
157.	Inophyllum G2	Xanthone	*Lawsonia inermis*	ACE2	**Docking score (AutoDock Vina)** -8.0 kcal/mol	
158.	Daphnorin	Coumarin	*Lawsonia inermis*	Nsp16<br>S-protein	**Docking score (AutoDock Vina)**<br>**Methyl transferase** -9.8 kcal/mol<br>**S-protein** -8.2 kcal/mol	
159.	Glycycoumarin	Coumarin	*Glycyrrhiza glabra*	Nsp16	**Docking score (AutoDock Vina)** -9.2 kcal/mol	

160.	Pterostilbene	Stilbenoid	<u>*Vitis rupestris*</u>	-	**In vitro against The SARS-CoV-2 strain NL/2020 in Vero E6**<br>**EC_{50}** 19 µM<br>**EC_{90}** 47µM<br>**In air-liquid interface (ALI) cultured human primary bronchial epithelial cells:**<br>virus titer was reduced with 1.2 Log (corresponding to 87.5% reduction)	[67]
161.	Anthrachinolinchinon	Quinolone	Synthetic	Mpro	**Docking score (AutoDockVina tool 1.5.6)** − 10.1 kcal/mol	[68]
162.	3-(4-chlorophenyl) isoxazol-5-yl) methyl-(2_,3_)-2,3 dihydroxyolean-12 en-28-oate	Chlorinated isoxazole linked maslinic acid	Synthetic	Mpro	**Docking score (**MOE 2019.012) -8.00 kcal/mol<br>**In vitro activity against NRC-03-nhCoV in Vero E6**<br>**IC_{50}** 4.12 µM	[69]
163.	Cnicin	Terpene	*Cnicus benedictus* extract	RdRp<br>ADP ribose phosphatase<br>Nsp15<br>NRP-1<br>AAK1<br>GAK	**Docking scores (AutoDock Vina)**<br>RdRP -9.7 kcal/mol<br>ADP ribose phosphatase -9.2 kcal/mol<br>nsp15 -9.8 kcal/mol<br>NRP-1 -11.5 kcal/mol<br>AAK1 -9.1 kcal/mol<br>GAK -8.2 kcal/mol<br>**ΔG (FEP)**<br>RdRP -10.3 kcal/mol<br>ADP ribose phosphatase -10.1 kcal/mol<br>Nsp15 -9.3 kcal/mol<br>NRP-1 -10.9 kcal/mol<br>AAK1 −9.5 kcal/mol<br>GAK −8.6 kcal/mol<br>**in vitro activity against hCoV-19/Egypt/NRC-3/202o in Vero E6**<br>**IC_{50}** 1.18 µg/mL	[70]
164.	Pomegranate peel extract (PPE)	Polyphenol-rich extracts	*Punica granatum*	RBD Of S-protein<br>ACE2<br>Mpro	**In vitro against SPIKE-ACE2**<br>**IC_{50}** 0.049 mg/ml.<br>**% Inhibition of Mpro** at 0.2 mg/ml = 80%	[71]
165.	Phenformin	<u>Biguanide</u>	Synthetic	PLpro	**Docking score (Glide)** -7.232<br>**ΔG (MM/GBSA)** -56.6 kcal/mol	[72]
166.	Quercitrin	<u>Flavonoid</u>	*Fagopyrum tataricum*		**Docking score (Glide)** -7.757<br>**ΔG (MM/GBSA)**-40.9 kcal/mol	
167.	Ritonavir	<u>Thiazole</u>	Synthetic		**Docking score (Glide)** -7.393<br>**ΔG (MM/GBSA)**- -37.6 kcal/mol	
168.	Deoxycylindrospermopsin	Polyketide	*Cylindrospermopsis sp.*	PLpro<br>Mpro	**Docking score (AutoDock Vina)**<br>Mpro -8.6 ± 0.02 kcal/mol<br>PLpro -7.9 ± 0.04 kcal/mol<br>**Binding energy (MM-PBSA)**<br>Mpro -45.44 ±1.66 kcal/mol<br>PLpro -41.39±1.59 kcal/mol	[73]
169.	Molnupiravir	Ribonucleoside analogue	Synthetic	RdRp	**In vitro against Adapted virus in**<br>**EC_{50}**<br>**Huh7.5** 8.5 µM<br>**Calu-1** 2.7 µM<br>**A549** 1.3 µM	[23]
					**In vivo in immunodeficient mice implanted with human lung tissue (human lung-only mice (LoM):**<br><br>**Therapeutic effect**<br>At 24h after exposure to SARS-CoV-2 twice daily (*P* = 0.0002) and virus titres reduced by 4.4 logs<br>At 48h after exposure to SARS-CoV-2 twice daily (*P* = 0.0019) and virus titres reduced by 96% (1.5 logs)<br><br>**Prophylaxis effect**<br>12h before exposure to SARS-CoV-2	[74]

					virus titres reduced by over 100,000-fold in 2 independent experiments (*P* = 0.0002 and *P* = 0.0068).	
					**In vitro in Calu-3 cells against Wild type (WT) SARS-CoV-2 (isolate BetaCoV /Munich /BavPat1 /2020) and the Omicron variant**<br>**IC_{50}**<br>WT 1.965 µM<br>Omicron variant 0.7556 µM	[75]
					**In vivo using rhesus macaque model treated with 75 mg/kg (n = 8) or 250 mg/kg (n = 8) twice daily by oral gavage for 7 days**	[76]
170.	Nilotinib	Pyrimidine derivative	Synthetic	Mpro	**Docking score (PyRX/AutoDock Vina)** −9.2 kcal/mol<br>**In vitro against SARS-CoV-2 in Vero-E6 cells**<br>**EC_{50}** 2.6 µM	[28]
171.	Bemcentinib	Benzocycloheptene			**Docking score (PyRX/AutoDock Vina)** −10.4 kcal/mol<br>**In vitro against SARS-CoV-2 in Vero-E6 cells**<br>**EC_{50}** 1.1 µM	
172.	(3R,4R)-4-Amino-1-[[4-[(3-methoxyphenyl)amino]pyrrolo[2,1-f][1,2,4]triazin-5-yl]methyl]piperidin-3-ol **(BMS-690514)**	Piperidin			**Docking score (PyRX/AutoDock Vina)** −8.0 kcal/mol<br>**In vitro against SARS-CoV-2 in Vero-E6 cells**<br>**EC_{50}** 32.8 µM	
173.	Gitoxin	Cardiac glycoside	*Digitalis purpurea*	S-protein	**Gibbs free energy(ΔG) (Autodock 4.2)** −17.84 kcal/mol	[77]
174.	Dicumarol	Coumarin	*Viola arvensis*		**Gibbs free energy(ΔG) (Autodock 4.2)** −12.05 kcal/mol	
175.	Diosgenin	Saponin	*Ophiopogon intermedius*		**Gibbs free energy(ΔG) (Autodock 4.2)** −10.75 kcal/mol	
176.	Spirostan	Steroid	*Allium suvorovii*	Mpro	**Gibbs free energy(ΔG) (Autodock 4.2)** −9.36 kcal/mol	
177.	N-(3-acetylglycyrrhetinoyl)-2-aminopropanol	Terpene	Semisynthetic		**Gibbs free energy(ΔG) (Autodock 4.2)** −8.75 kcal/mol	
178.	Glyasperin A	Flavonoid	*Glycyrrhiza glabra*	Nsp15	**Docking score (Autodock vina)** - 9.2 kcal/mol<br>**ΔG (MM-PBSA)** -124.036 **kJ/mol**	[4]
179.	Dihydrotanshinone I	Terpene	*Salvia miltiorrhiza*	PLpro	**In vitro against the SARS-CoV-2 in VERO E6 cells**<br>**EC_{50}** 8 µM<br>**In vitro against PLpro**<br>**IC_{50}** 0.59 µM	[78]
180.	2,3-Dihydroamentoflavone	Flavonoid	*Cycas revoluta*	Mpro	**Docking score (MTi AutoDock)** -12.47 kcal/mol<br>**ΔG (MM/GBSA)** −52.35±4.54 kcal/mol	[35]
181.	Podocarpusflavon-B	Flavonoid	*Selaginella sinensis*		**Docking score (MTi AutoDock)** −13 kcal/mol<br>**ΔG (MM/GBSA)** −38.92±0.59 kcal/mol	
182.	Quercimeritrin 6"-O-L-arabinopyranoside	Flavonoid	*Knautia montana*		**Docking score (MTi AutoDock)** −13.3 kcal/mol<br>**ΔG (MM/GBSA)** −62.01±5.15 kcal/mol	
183.	Sepantronium bromide	Imidazolium-based compound	Synthetic	PLpro	**In vitro against PLpro**<br>**IC_{50}** 2.47 µmol/L.<br>**In vitro against SARS-CoV-2 (nCoV-2019 BetaCoV /Wuhan/WIV04/2019) in Vero E6 cells**<br>**EC_{50}** 170 nmol/L	[79]
184.	Kobophenol A	Stilbenoid	*Caragana sinica*	Block interaction between the ACE2 receptor and S1-RBD	**In vitro against spike (RBD)**<br>**IC_{50}** 1.81 ± 0.04 µM<br>**In vitro against SARS-CoV-2 in VeroE6-EGFP cells**<br>**EC_{50}** 71.6 µM.<br>**ΔG (MM/PBSA)**<br>ACE2/spike interface −19.0 ± 4.3 kcal/mol<br>ACE2 pocket −24.9 ± 6.9 kcal/mol<br>**Docking score (AutoDock version 4.2)**	[80]

					ACE2/spike interface −11.15 kcal/mol<br>ACE2 binding pocket −9.98 kcal/mol	
185.	(1R,2S,5S)-6,6-dimethyl-N-((S)-1-oxo-3-((S)-2-oxopyrrolidin-3-yl)propan-2-yl)-3-(2-(4- (trifluoromethoxy) phenoxy) acetyl)-3-azabicyclo[3.1.0]hexane-2-carboxamide	Pyrrolidin	Synthetic	Mpro	**In vitro against Mpro**<br>**IC_{50}** 15.2±0.4 nM<br>**In vitro against SARS-CoV-2 in *HPAEpiC***<br>**IC_{50}** 1.2± 0.1 nM<br>**In vivo using mice** infected with dose of SARS-CoV-2:<br>**Dose of SARS-CoV-2 (2 × 106 TCID50)**<br>Dose of MI-09 [50 mg/kg p.o. twice daily (bid) or 50 mg/kgi.p. once daily (qd)]<br>MI-30 (50mg/kg i.p. qd)<br>**The mean viral RNA loads in the lung tissue**<br>At 1 dpi,P < 0.05<br>At 3 and 5 dpi, undetectable<br><br>**Dose of SARS-CoV-2 (5 × 106 TCID50)**<br>Dose of 100 mg/kg for both i.p. and p.o. administration of MI-09 and MI-30<br>**The mean viral RNA loads in the lung tissue**<br>At 3 dpi, P < 0.05,<br>At 5 dpi undetectable	[81]
186.	(1S,3aR,6aS)-2-(2-(2,4-dichlorophenoxy)acetyl)-N-((S)-1-oxo-3-((S)-2-oxopyrrolidin-3-yl)propan-2-yl)octahydrocyclopenta[c]pyrrole-1-carboxamide	Pyrrolidin	Synthetic	Mpro	**In vitro against Mpro**<br>**IC_{50}** 17.2± 0.6 nM<br>**In vitro against SARS-CoV-2 in *HPAEpiC***<br>**IC_{50}** 1.1± 0.2 nM	
187.	Leporin A	Pyridone	*Aspergillus* sp.	Mpro	**Docking score (AutoDock)** -9.4 kcal/mol	[82]
188.	3-[[6-[3-[4,5-dihydroxy-6-[[(E)-3-(4-hydroxyphenyl)prop-2-enoyl]oxymethyl]-3-[3,4,5-trihydroxy-6-(hydroxymethyl)oxan-2-yl]oxyoxan-2-yl]oxy-7-hydroxy-2-(4-hydroxyphenyl)chromenylium-5-yl]oxy-3,4,5-trihydroxyoxan-2-yl]methoxy]-3-oxopropanoic acid **(Compound ID 131751762)**	Anthocyanidin glycoside	Natural	Mpro	**Docking score (Glide)** -10.30 kcal/mol<br>**ΔG (MM-GBSA)** -68.40 kcal/mol	[83]
189.	[6-[2-[2-(3,4-dihydroxyphenyl)-7-hydroxy-5-[3,4,5-trihydroxy-6-(hydroxymethyl)oxan-2-yl]oxychromenylium-3-yl]oxy-4,5-dihydroxy-6-(hydroxymethyl)oxan-3-yl]oxy-3,4,5-trihydroxyoxan-2-yl]methyl 4-hydroxybenzoate **(Compound ID 131831710)**			Mpro	**Docking score (Glide)** -13.59 kcal/mol<br>**ΔG (MM-GBSA)** -45.66 kcal/mol	

190.	Broussochalcone A	Phenolic	*Broussonetia papyrifera*	Mpro	**Docking score (AutoDock Vina)** -8.1 kcal/mol<br>**ΔG (MM-GBSA)** -50.91 kcal/mol	[84]
191.	Papyriflavonol A				**Docking score (AutoDock Vina)** -7.9 kcal/mol<br>**ΔG (MM-GBSA)** -47.28 kcal/mol	
192.	3'-(3-methylbut-2-enyl)-3',4',7-trihydroxyflavane				**Docking score (AutoDock Vina)** -8.2 kcal/mol<br>**ΔG (MM-GBSA)** -51.59 kcal/mol	
193.	Broussoflavan A				**Docking score (AutoDock Vina)** -7.8 kcal/mol<br>**ΔG (MM-GBSA)** -41.32 kcal/mol	
194.	Kazinol F				**Docking score (AutoDock Vina)** -8.1 kcal/mol<br>**ΔG (MM-GBSA)** -56.23 kcal/mol	
195.	Kazinol J				**Docking score (AutoDock Vina)** -8.0 kcal/mol<br>**ΔG (MM-GBSA)** -41.98 kcal/mol	
196.	2-(4-chlorobenzyl)benzo[d][1,2]selenazole-3(2H)-one	Benzoisoselenazolone	Synthetic	Mpro	**In vitro against Mpro**<br>**IC_{50}**.824 μM<br>**In vitro against SARS-CoV-2 (nCoV-2019BetaCoV/Wuhan/WIV04/2019) in Vero E6 cells**<br>**EC_{50}** 1.8 μM	[85]
197.	Isothymol	Terpene	*Ammoides verticillata*	ACE2	**Docking score (MOE)** -5.7853 kcal/mol	[86]
198.	Trichostatin A	Hydroxamic acid	*Streptomyces platensis*	Mpro	**Docking score (CovalentDock)** -12.6<br>**In vitro against Mpro**<br>**IC_{50}** 37.97±3.68 μM<br>**In vitro against SARS-CoV-2 in VeroE6 cells**<br>**EC_{50}** 1.5±0.3 μM<br>**In vitro against SARS-CoV-2 in Caco-2 cell**<br>**EC_{50}** 2.7±0.8 μM	[87]
199.	Crocin	Carotenoid	*Crocus Sativus L*	Mpro	**Docking score (Autodock vina)** -8.2 Kcal/mol	[88]
200.	Digitoxigenin	Cardenolide	*Nerium Oleander*		**Docking score (Autodock vina)** -7.2 Kcal/mol	
201.	β-Eudesmol	*Terpene*	*Lauris Nobilis L*		**Docking score (Autodock vina)** -7.1 Kcal/mol	
202.	Sesamin	Lignan	*Sesamum indicum L*	Mpro	**Docking score (AutoDock Vina)** −6.7 kcal/mol<br>**ΔG (MM-PBSA)** −145.511 ± 17.054 kJ/mol	[89]
203.	Sesaminol	Furofuran			**Docking score (AutoDock Vina)** −6.6 kcal/mol<br>**ΔG (MM-PBSA)** −149.078 ± 9.043 kJ/mol	
204.	Sesamolin	Benzodioxole			**Docking score (AutoDock Vina)** −6.4 kcal/mol<br>**ΔG (MM-PBSA)** −211.240 ± 14.034 kJ/mol	
205.	Sesamolinol	Phenol			**Docking score (AutoDock Vina)** −6.1 kcal/mol<br>**ΔG (MM-PBSA)** −199.110 ± 15.881 kJ/mol	
206.	Alizarin	Dihydroxyanthraquinon	*Rheum emodi*	The three active sites of RNA binding domain of nucleocapsid phosphoprotein (N)	**Docking score (Schrodinger's maestro 12.0)**<br>**site A** −8.441 Kcal/ mol<br>**site B** −6.598 Kcal/ mol<br>**site C** -8.841 Kcal/ mol<br>**ΔG (prime MM/GBSA)**<br>**site A**-31.2891 Kcal/ mol<br>**site B**-33.59 Kcal/ mol<br>**site C** -33.84 Kcal/ mol	[90]
207.	Aloe-emodin				**Docking score (Schrodinger's maestro 12.0)**<br>**site A** −8.508 Kcal/ mol<br>**site B** −6.433 Kcal/ mol<br>**site C** -8.508 Kcal/ mol<br>**ΔG (prime MM/GBSA)**<br>**site A** -25.45 Kcal/ mol<br>**site B** -36.92 Kcal/ mol<br>**site C** -32.61 Kcal/ mol	

208.	Anthrarufin				**Docking score (Schrodinger's maestro 12.0)**<br>**site A** −8.456 Kcal/ mol<br>**site B** −6.354 Kcal/ mol<br>**site C** -8.538 Kcal/ mol<br>**ΔG (prime MM/GBSA)**<br>**site A** -38.099 Kcal/ mol<br>**site B** -34.9567 Kcal/ mol<br>**site C** -39.84 Kcal/ mol	
209.	Lurasidone	Organosulfur compounds	Synthetic	Mpro<br>PLpro<br>S-protein<br>Helicase<br>RdRp	**Docking score (AutoDock Vina)**<br>Mpro −8.4 kcal/mol<br>PLpro −7.3 kcal/mol<br>S-protein −8.1 kcal/mol<br>Helicase −9 kcal/mol<br>RdRp −8.2 kcal/mol<br>**ΔG (MM-PBSA)**<br>Mpro −28.37 kcal/mol<br>PLpro −16.31 kcal/mol<br>S-protein −10.94 kcal/mol<br>Helicase −34.60 kcal/mol<br>RdRp −19.53 kcal/mol	[91]
210.	Lurasidone exo				**Docking score (AutoDock Vina)**<br>Mpro −8.2 kcal/mol<br>PLpro −6.8 kcal/mol<br>S-protein −8.1 kcal/mol<br>Helicase −8.9 kcal/mol<br>RdRp −8 kcal/mol<br>**ΔG (MM-PBSA)**<br>Mpro −18.22 kcal/mol<br>PLpro −16.93 kcal/mol<br>S-protein –22.37 kcal/mol<br>Helicase −29.47 kcal/mol<br>RdRp −14.72 kcal/mol	
211.	(2r,15r)-2-[(1-aminoisoquinolin-6-Yl)amino]-4,15,17-trimethyl-7-[1-(1h-tetrazol-5-Yl)cyclopropyl]-13-Oxa-4,11-diazatricyclo[14.2.2.1~6,10~]henicosa-1(18),6(21),7,9,16,19-hexaene-3,12-dione	Quinoline derivative	Synthetic	Mpro	**Docking score (Autodock vina)** -10.6 kcal/mol	[92]
212.	N-[[3-(3-hydroxyprop-1-ynyl)phenyl]methyl]-4-methoxypentanamide	Pentamide	Synthetic		**Docking score (Autodock vina) -**10.2 kcal/mol	
213.	[4-(4-Fluorobenzoyl)phenyl]-(4-fluorocyclohexyl)methanone	Methanone	Synthetic		**Docking score (Autodock vina)** -9.6 kcal/mol	
214.	Resveratrol	Polyphenol	*Vitis Vinifera*	ACE2	**In vitro against with SARS-CoV-2(BetaCoV/Shenzhen/SZTH-003/2020) in Vero**<br>**EC_{50}** 4.48 μM.	[93]
					**Docking score (Autodock vina)** -8.0 Kcal/mol<br>**The total binding energy:**<br>MMPBSA -20.2657 kcal/mol<br>MM-GBSA -23.8889 kcal/mol	[94]
					**In vitro against The SARS-CoV-2 strain NL/2020 in Vero E6**<br>**EC_{90}**119 μM<br>**EC_{50}** 66 μM<br><br>**In PBEC differentiated under ALI**	[67]

					**culture conditions**<br>**At 48 hpi :**<br>Virus titer reduced with 2.1 Log (corresponding to 99.3% reduction)	
215.	Epigallocatechin-3-gallate (EGCG)	Polyphenol catechin	*Camellia sinensis*	Mpro	**Docking score (AutoDock Vina 1.1.2)** −7.9 kcal/mol<br>**In vitro against Mpro**<br>**IC_{50}** 0.874±0.005 μM.	[95]
216.	Cichoriin	Coumarin	*Taraxacum officinale*	RdRp (RTP site)<br>ExoN<br>Nsp3 (AMP site)<br>PLpro	**Docking score (COVID-19 Docking Server)**<br>RdRp -9.5<br>ExoN -8.8<br>Nsp3 -7.1<br>PLpro -8.3	[96]
217.	10-Hydroxyusambarensine	Alkaloids	*Strychnos usambarensis (Loganiaceae)*	Mpro	**Docking score (AutoDock vina 4.2)** -10.0 kcal/mol	[97]
218.	Cryptoquindoline		*Cryptolepis sanguinolenta (Periplocaceae)*		**Docking score (AutoDock vina 4.2)** -9.7 kcal/mol	
219.	6-Oxoisoiguesterin	Terpene	*Salacia madagascariensis*		**Docking score (AutoDock vina 4.2)** -9.1 kcal/mol	
220.	22-Hydroxyhopan-3-one		*Cassia siamea (Fabaceae)*		**Docking score (AutoDock vina 4.2)** -8.6 kcal/mol	
221.	Carnosol	Terpene	*Rosmarinus officinalis*	Mpro	**Docking score (Autodock)** -8.2 kcal/mol	[98]
222.	Arjunglucoside-I	Glycoside	*Terminalia chebula*		**Docking score (Autodock) -**7.88 kcal/mol	
223.	Rosmanol	Terpene	*Rosmarinus officinalis*		**Docking score (Autodock)** -7.99 kcal/mol	
224.	Nafamostat	Guanidines derivatives	Synthetic	TMPRSS2	**Docking score (Autodock Vina)** −7.8 kcal/mol	[39]
					**Docking score (LeDock)** -8.61 kcal/mol<br>**ΔG (MMGBSA)** -30.83 ± 3.33 kcal/mol	[64]
225.	Meloxicam	Benzothiazine	Synthetic		**Docking score (Autodock Vina)** −8.3 kcal/mol	[39]
226.	Ganodermanontriol	Terpene	*Ganoderma lucidum*		**Docking score (Autodock Vina)** −8.1 kcal/mol	
227.	Columbin	Terpene	*Tinospora cardifolia*		**Docking score (Autodock Vina)** −8.2 kcal/mol	
228.	Proanthocyanidin A2	Tannins	*Litchi chinensis*		**Docking score (Autodock Vina)** −7.9 kcal/mol	
229.	Jatrorrhizine	Alkaloid	*Tinospora cardifolia*		**Docking score (Autodock Vina)** −7.5 kcal/mol	
230.	Baicalein	Flavonoid	*Scutellaria baicalensis*	Mpro	**Docking score (Glide)** -8.277<br>**In vitro against Mpro**<br>**IC_{50}** 0.39 μM<br>**In vitro against SARS-CoV-2 in Vero cells**<br>**EC_{50}** 2.9 μM	[40]
				TMPRSS2	**Docking score (Autodock Vina)** −7.7 kcal/mol	[39]
231.	Ethanol extract	Flavonoid	*Scutellaria baicalensis*	Mpro	**In vitro against Mpro**<br>**IC_{50}** 8.52 μM /ml<br>**In vitro against SARS-CoV-2 in Vero cells**<br>**EC_{50}** 0.74 μM /ml	[40]
232.	Scutellarein	Flavonoid	*Genus Scutellaria and Erigerontis herba*		**Docking score (Glide)** -8.823<br>**In vitro against Mpro**<br>**IC_{50}** 5.8 μM	
233.	Dihydromyricetin	Flavonoid	*Ampelopsis japonica*		**Docking score (Glide)** -7.966<br>**In vitro against Mpro**<br>**IC_{50}** 1.20 μM	

234.	Quercetagetin	Flavonoid	*Eriocaulon buergerianum*		**Docking score (Glide)** -9.109<br>**In vitro against Mpro**<br>**IC_{50}** 1.24 µM	
235.	1-Hydroxyaleuritolic acid 3-p-hydroxybenzoate	Terpene	*Maprounnea Africana.*	Mpro<br>PLpro	**Dockind score (Autodock Vina)**<br>Mpro -8.5 kcal/mol<br>PLpro -8.0 kcal/mol	[99]
236.	(1R,2S,5S)-N-{(1S)-1-Cyano-2-[(3S)-2-oxopyrrolidin-3-yl]ethyl}-6,6-dimethyl-3-[3-methyl-N-(trifluoroacetyl)-L-valyl]-3-azabicyclo[3.1.0]hexane-2-carboxamide (PF-07321332)<br>**(Nirmatrelvir)**	Pyrrolidin	Synthetic	Mpro	**In vitro against SARS-CoV-2 (USA_WA1/2020) in Vero E6 with CP-100356**<br>**EC_{50}** 74.5 nM<br>**EC_{90}** 155 nM<br>**in A549-ACE2 cells**<br>**EC_{50}** 77.9 nM<br>**EC_{90}** 215 nM<br>**in dNHBE cells day 3**<br>**EC_{50}** 61.8 nM<br>**EC_{90}** 181 nM<br>**in dNHBE cells day 5**<br>**EC_{50}** 32.6 nM<br>**EC_{90}** 56.1 nM<br><br>**In vivo** using mouse-adapted SARS-CoV-2 (SARS-Cov-2 MA10) model treated orally (300 mg/kg and 1000 mg/kg. 4 days post infection,<br>**Mean lung titers**<br>300 mg/kg 3.533 ± 0.187 CCID50 log10/ml<br>1000 mg/kg 3.02 ± 0.423 CCID50 log10/ml	[100]
					**In vitro in Calu-3 cells against Wild type (WT) SARS-CoV-2 (isolate BetaCoV /Munich /BavPat1 /2020) and the Omicron variant**<br>**IC_{50}**<br>WT 0.1765 µM<br>Omicron 0.02462 µM	[75]

					**in vitro against**<br>**Vero E6-GFP cells**<br>Bavpat 90 ± 10 nM<br>B.1.1.7 270 ± 40 nM<br>B.1.351 140 ± 40 nM<br>B.1.1.28.1 280 ± 20 nM<br>B.1.617.2 210 ± 30 nM<br>**A549**<br>Bavpat 100 ± 70 nM<br>B.1.1.7 110 ± 60 nM<br>B.1.351 70 ± 20 nM<br>B.1.1.28.1 120 ± 40 nM<br>B.1.617.2 260 ± 50 nM<br><br>**In vivo** using Syrian hamsters were intranasally infected with the SARS-CoV2 **beta variant** and orally treated with PF-332 either at 125 or 250 mg/kg/dose BID for 4 consecutive days whereby treatment was initiated immediately before infection<br><br>The reduction in viral RNA levels in the lungs<br>At 125 mg/kg 1.1 log10 (P = 0.0007)<br>At 250 mg/kg 5.8 log10 (P < 0.0001)<br><br>The reduction of infectious viral titers<br>At 125 mg/kg 0.7 log10 (P = 0.03)<br>At 250 mg/kg 4.4 log10 (P < 0.0001)<br><br>**In vivo** infected with **delta VoC**<br>at 250 mg/kg BID<br>The reduction in viral<br>RNA levels in the lungs 2.5 log10 (P= 0.0022)<br>The reduction of infectious<br>viral titers 4.2 log10 (P =0.0022)	[101]
237.	Delta-viniferin	Stilbene	*Vitis vinifera*	Mpro<br>RdRp<br>ACE2	**Docking score (Autodock vina)**<br>MPro -8.4 kcal/mol<br>RdRP -8.3 kcal/mol<br>ACE2 -8.4 kcal/mol	[26]
238.	Taiwanhomoflavone A	Flavonoid	*Cephalotaxus wilsoniana*		**Docking score (Autodock vina)**<br>MPro -9.6 kcal/mol<br>RdRP -9.8 kcal/mol<br>ACE2 -7.8 kcal/mol	
239.	Lactucopicrin 15 oxalate	Terpene	*Lactuca Virosa*		**Docking score (Autodock vina)**<br>MPro -8.2 kcal/mol<br>RdRP -7.6 kcal/mol<br>ACE2 -8.3 kcal/mol	
240.	Nympholide A	Flavonoid	*Nymphaea lotus*		**Docking score (Autodock vina)**<br>MPro -7.8 kcal/mol<br>RdRP -9.3 kcal/mol	
241.	Afzelin	Flavonoid	*Nymphaea odorata*		**Docking score (Autodock vina)**<br>MPro -8.8 kcal/mol<br>RdRP -8 kcal/mol<br>ACE2-7.1 kcal/mol	
242.	Biorobin	Flavonoid	*Ficus benjamina*		**Docking score (Autodock vina)**<br>MPro -9.3 kcal/mol<br>RdRP -9.1 kcal/mol<br>ACE2 -7.4 kcal/mol	
243.	Phyllaemblicin B	Terpene	*Phyllanthus emblica*		**Docking score (Autodock vina)**<br>MPro -8 kcal/mol<br>RdRP -9.3 kcal/mol<br>ACE2 -7 kcal/mol	
244.	Dalbavancin	Lipoglycopeptide	Semi-synthetic	ACE2	**Docking score (LibDock)** -68.78 kcal/mol<br>**In vitro against SARS-CoV-2 in Vero E6 cells**	[102]

					**EC_{50}** ~12 nM.<br>**In vivo in mouse and rhesus macaque models:**<br>**HACE2 mice**<br>A single dose of dalbavancin (130 mg/kg intraperitoneal) viral load decreased to<br>At 24 hpi ~103 copies /µg $P < 0.01$<br>At 72 hpi ~102 copies/µg $P < 0.05$<br>**Rhesus macaque model**<br>P=0.0042	
245.	Cinnamic acid	Carboxylic acid	*Bromeliad*	ACE2	**Docking score (Autodock 1.5.6)** −5.20 kcal/mol	[103]
246.	Thymoquinone	1,4-benzoquinone	*Nigella Sativa*		**Docking score (Autodock 1.5.6)** −4.71 kcal/mol	
247.	Andrographolide **(Kalmegh)**	Terpene	*Andrographis Paniculata*		**Docking score (Autodock 1.5.6)** −4.00 kcal/mol	
				IL-6	**Docking Score (AutoDock4.2.6)** –9.6 kcal/mol<br>**ΔG (MM-GBSA)** -44.8 kcal/mol	[52]
248.	Curcumin	Phenolic	*Curcuma longa*	Mpro	**Docking score (PyRx-Python Prescription 0.8)**<br>–6.50 ± 0.36 kcal/mol<br>**ΔG(kcal/mol)**<br>MM/PBSA –11.29 ± 0.25 kcal/mol<br>MM/GBSA –18.21 ± 0.25 kcal/mol	[104]
249.	Apigenin 7-glucoside-4'-p-coumarate	Apigenin analogue	*Matricaria chamomilla*	Mpro	**Docking score (AutodockVina)** −8.8 kcal/mol	[105]
250.	β-boswellic acid	Terpene	*Boswellia serrata*	E-protein	**Docking score (PyRX)** -9.1 kcal/mol	[5]
251.	<u>Limonin</u>	Terpene	<u>*Citrus tankan*</u>	RBD of S-protein	**Docking score (AutoDock Vina 1.1.2)**<br>WT -8.2 kcal/mol<br>Delta -8.3 kcal/mol<br>Omicron -8.3 kcal/mol	[6]
252.	Combination of molnupiravir and nirmatrelvir	-	-	Mpro	**Synergy score for molnupiravir and nirmatrelvir combination using SynergyFinder**<br>WT 4.532<br>Omicron 4.131	[75]
253.	Schaftoside	Flavonoid	*Glycyrrhiza uralensis*	Mpro<br>PLpro	**Docking score (AutoDock Vina)**<br>Mpro -8.4 kcal/mol<br>PLpro -8.5 kcal/mol<br>**In vitro against** :<br>**IC_{50}**<br>Mpro 1.73 ± 0.22 µmol/L<br>PLpro 3.91 ± 0.19 µmol/L<br><br>**In vitro against SARS-CoV-2 (nCoV2019BetaCoV/ Wuhan / WIV04/2019) in Vero E6 cells**<br>**EC_{50}** 11.83 ± 3.23 mmol/L	[106]
254.	Licorice-saponin A3	Terpene	*Glycyrrhiza uralensis*	Nsp7	**Docking score (Autodock Vina)** −8.7 kcal/mol<br>**In vitro against SARS-CoV-2 (nCoV-2019BetaCoV/Wuhan/WIV04/2019) in Vero E6 cells**<br>**EC_{50}** 75 nM	[107]
255.	Glycyrrhetinic acid			RBD of S-protein	**Docking score (Autodock Vina)** -7.29 kcal/mol<br>**In vitro against SARS-CoV-2 (nCoV-2019BetaCoV/Wuhan/WIV04/2019) in Vero E6 cells**<br>**EC_{50}** 3.17 µM<br>**In vitro against S-protein**<br>**IC_{50}** 10.9 µM	
256.	Silibinin	Flavonolignan	*Silybum marianum*	S-protein<br>Mpro<br>RdRp	**Docking score (Schrödinger suite 12.7 using Glide score)**<br>S protein -7.78 kcal/mol<br>Mpro -7.05 kcal/mol<br>RdRP -7.15 kcal/mol<br>**In vitro against** :<br>**S protein**<br>**IC_{50}** 0.029 µM<br>**Mpro**	[108]

					**IC_{50}** 0.021 µM<br>**RdRP**<br>**IC_{50}** 0.042 µM<br>**In vitro against SARS-CoV-2 strain NRC-03-nhCoV in Vero-E6 cells**<br>**IC_{50}** 31.2 µM	
257.	1,2,3,4,6-O-Pentagalloylglucose (PGG)	Phenolic	*Punica granatum L.*	RdRp<br>Mpro	**In vitro against SARS-CoV-2 (βCoV/KOR/KCDC03/2020) in Vero cells**<br>**IC_{50}** 15.02 ± 0.75 µM<br>**In vitro against RdRp**<br>**IC_{50}** 5.098 ± 1.089 µM<br>**In vitro against Mpro**<br>**IC_{50}** 25.26 ±1.04 µM	[109]
258.	(3R,4R,5R)-2-(4-aminopyrrolo[2,1-f][1,2,4]triazin-7-yl)-2-cyano-5-((isobutyryloxy)methyl)tetrahydrofuran-3,4-diyl bis(2-methylpropanoate**)**	Adenosine nucleoside	Synthetic	RdRp	**in vitro against SARS-CoV-2-fLUC in normal human bronchial epithelial (NHBE)**<br>**EC_{50}** 125± 0.4 nM<br><br>**In vivo in mice infected with 10^4 PFU SARS-CoV-2 MA10 treated with 10 mg/kg, or 30 mg/kg of the drug 8 hours post infection twice daily**<br>**Reduction of viral lung titers**<br>10 mg/kg (p=0.0001)<br>30 mg/kg (p<0.000)<br>**Reduction of lung congestion**<br>10 mg/kg (p=0.0006)<br>30 mg/kg (p<0.0001)<br>**Only mice treated with 30 mg/kg had significantly reduced acute lung injury (ALI) scores (p=0.0002)**	[110]
259.	Daidzin	Flavonoid	<u>*Thermopsis lanceolata*</u>	Nsp16	**Docking score (Autodock Vina tool compiled in the PyRx 0.8)** -8.3 kcal/mol	[18]
260.	Luteoloside	Flavonoid	*Lonicera japonica*		**Docking score (Autodock Vina tool compiled in the PyRx 0.8)** -8.3 kcal/mol	
261.	(Z)-N'-(2,2-dimethyl-5-oxo-3,4-dihydro-2H-benzo[h]chromen-6(5H)-ylidene)nicotinohydrazide	Naphthoquinones	Synthetic	Nsp9	**Docking score (ChemPLP score)** 56.34<br>**ΔG (MM-PBSA)** -55.681± 31.008 kJ/mol	[111]
262.	2-(3-methylbut-2-en-1-yl)-3-((nicotinoyloxy)amino)naphthalene-1,4-dione				**Docking score (ChemPLP score)** 64.23<br>**ΔG (MM-PBSA)** -43.432±15.626 kJ/mol	
263.	Piperine	N-acylpiperidine	*Piper boehmeriifolium*	Mpro<br>RBD of S-protein	**Docking score (Autodock vina)**<br>**Mpro** -7.3 kcal/mol<br>**RBD of S-protein** -6.4 kcal/mol<br>**ΔG (MMPBSA)**<br>Mpro -37.971 ± 0.271 kJ/mol<br>RBD of S-protein -5.533 ± 0.893 kJ/mol	[112]
264.	Somniferine	Alkaloid	*Withania somnifera*	Mpro	**Docking score (AutoDock Vina-based YASARA)** 9.62 kcal/mol	[58]
265.	Tinocordiside	Glycoside	*Tinospora cordifolia*		**Docking score (AutoDock Vina-based YASARA)** 8.10 kcal/mol	
266.	Vicenin		*Ocimum sanctum*		**Docking score (AutoDock Vina-based YASARA)** 8.97 kcal/mol	
267.	Isorientin 4'-O-glucoside 2''-O-p-hydroxybenzoagte				**Docking score (AutoDock Vina-based YASARA)** 8.55 kcal/mol	
268.	Ursolic acid	Terpene			**Docking score (AutoDock Vina-based YASARA)** 8.52 kcal/mol	

269.	Peonidin 3-O-glucoside	Anthocyanin	<u>*Ribes uva-crispa*</u>	Mpro	**Docking score (Autodock vina)** -9.4 kcal/mol<br>**ΔG (MM-PBSA)** -274.623 ± 27.613 kJ/mol	[113]
270.	Kaempferol 3-O-β-rutinoside	Flavonoid	*Selliguea feei*		**Docking score (Autodock vina)** -9.3 kcal/mol<br>**ΔG (MM-PBSA)** -343.212 ± 33.998 kJ/mol	
271.	4-(3,4-Dihydroxyphenyl)-7-methoxy-5-[(6-O-β-D-xylopyranosyl-β-D-glucopyranosyl)oxy]-2H-1-benzopyran-2-one	Pyran	*Hintonia standleyana*		**Docking score (Autodock vina)** -9.2 kcal/mol<br>**ΔG (MM-PBSA)** -259.970 ± 46.377 kJ/mol	
272.	Quercetin-3-D-xyloside	*Flavonoid*	*Prunus salicina*		**Docking score (Autodock vina)** -9.1 kcal/mol<br>**ΔG (MM-PBSA)** -268.184 ± 31.135 kJ/mol	
273.	Quercetin 3-O-α-L-arabinopyranoside	Flavonoid	*Ruprechtia polystachya*		**Docking score(Autodock vina)** -9.0 kcal/mol<br>**ΔG (MM-PBSA)** -229.655 ± 27.4 kJ/mol	
274.	Luteolin	Flavonoids	<u>*Verbascum lychnitis*</u>	Mpro	**Docking score (Autodock-4.2.6)** -8.14 kcal/mol<br>**ΔG (MM-PBSA)** -85.34 kJ/mol	[114]
275.	Viomycin	Peptide	*Streptomyces puniceus*	Mpro	**Dockig score (LeDock)** -13.6 kcal/mol<br>**ΔG (MM/PBSA)** −434.558 ± 41.901 kJ/mol	[115]
276.	Capastat	Peptide	*Streptomyces capreolus*		**Dockig score (LeDock)** -12.6 kcal/mol<br>**ΔG (MM/PBSA)** -385.087 ± 57.161 kJ/mol	
277.	Carfilzomib	Peptide	Synthetic		**Dockig score (LeDock)** -12.8 kcal/mol<br>**ΔG (MM/PBSA)** -103.171 ± 30.589 kJ/mol	
					**Docking score (Glide)** −8.6 kcal/mol<br>**ΔG(MMPBSA-WSAS)** −13.8 kcal/mol	[31]
278.	Saquinavir	Quinolin			**Dockig score (LeDock)** -12.4 kcal/mol<br>**ΔG (MM/PBSA)** −222.202 ± 19.214 kJ/mol	[115]
279.	Methanol extract	-	*Andrographis paniculate*	RdRp	**In vitro for (E4) against SARS-CoV2 in VeroE6 cells**<br>**IC_{50}**<br>E gene 1.18 µg<br>N gene 1.16 µg	[37]
280.	Indomethacin	<u>Indole</u> derivative	Synthetic	**Viral receptors:**<br>Mpro<br>RdRp<br>S-protein<br>PLpro<br>NSP16<br>NSP15<br><br>**Host receptors:**<br>Human Insulin-Degrading Enzyme<br><br>MZM-REP Domains of Mind bomb 1<br><br>Human Glutathione Peroxidase 1<br><br>Catalytic and Ubiquitin-associated domains of MARK1/PAR-1	**Docking score (AutoDock Tools 1.5.6 for AutoDock 4.2)**<br>Mpro -11.52 kcal/mol<br>RdRp -10.9 kcal/mol<br>S-protein -9.44 kcal/mol<br>PLpro -8.4 kcal/mol<br>NSP16 -8.36 kcal/mol<br>NSP15 -8.05 kcal/mol<br>Human Insulin-Degrading Enzyme -11.98 kcal/mol<br>MZM-REP Domains of Mind bomb 1 -10.57 kcal/mol<br>Human Glutathione Peroxidase 1 -9.65 kcal/mol<br>Catalytic and Ubiqutin-associated domains of MARK1/PAR-1 -9.53 kcal/mol<br>ACE2 -9.22 kcal/mol<br>Human Sirtuin homolog 5 -9.2 kcal/mol<br>Kinase and Ubiquitin-associated domains of MARK3/Par-1 -9.18 kcal/mol<br>NTF2 domain of Ras GTPase-activating protein-binding protein 1 -8.67 kcal/mol<br>Human plakophilin 2 isoform a (PKP2a) - 8.32 kcal/mol<br>SmgGDS-558 -8.2 kcal/mol<br>Human Heme Oxygenase-1 -8.1 kcal/mol<br>G3BP2 NTF2-like domain in complex with a peptide -4.18 kcal/mol<br><br>**In vitro against SARS-CoV-2 (NIV2020-770 isolate) in Vero CCL-81 cells**<br>**IC_{50}** 12 µM	[116]

				ACE2<br><br>Human Sirtuin homolog 5<br><br>Kinase and Ubiquitin-associated domains of MARK3/Par-1<br><br>NTF2 domain of Ras GTPase-activating protein-binding protein 1<br><br>Human plakophilin 2 isoform a (PKP2a)<br><br>SmgGDS-558<br><br>Human Heme Oxygenase-1<br><br>G3BP2 NTF2-like domain in complex with a peptide		
281.	H84T-BanLec	Lectin	Synthetic	S-protein	**In vitro against SARS-CoV-2 in VeroE6**<br>**EC_{50}**<br>HKU-001a 6.02 nM<br>B.1.1.529 (Omicron) 6.63 nM<br>B.1.617.2 (Delta) 5.87 nM<br>B.1.1.7 (Alpha) 7.71 nM<br>P.3 (Theta) 10.46 nM<br><br>**In vivo using golden Syrian hamster model at 4 dpi**<br>**Two groups received of 15 mg/kg once daily intraperitoneal**<br>6 h before virus challenge $P < 0.05$<br>24 h after virus challenge $P < 0.05$<br>**Two groups received 1.5 mg/kg once daily Intranasal**<br>6 h before virus challenge $P < 0.05$<br>24 h after virus challenge $P < 0.05$<br><br>So it has prophylactic and therapeutic effect	[117]
282.	Bisdemethoxycurcumin	Terpene	*Curcuma longa*	Mpro<br>Nsp15	**Docking score (AutoDock4 Tool)**<br>Mpro -7.3 Kcal/mol<br>Nsp15 -6.56 Kcal/mol	[14]
283.	Demethoxycurcumin	Terpene	*Curcuma zedoaria*		**Docking score (AutoDock4 Tool)**<br>Mpro -7.02 Kcal/mol<br>Nsp15 -7.51 Kcal/mol	
284.	Scutellarin	Flavonoid	*Scoparia dulcis*		**Docking score (AutoDock4 Tool)**<br>Mpro -7.13 Kcal/mol<br>Nsp15 -6.97 Kcal/mol	
285.	(S,E)-3-(2-((3S,4aR,6aS,7R,10aS,10bR)-6a,10b-dimethyl-8-methylene-3-(3-nitrophenyl)decahydro-1H-naphtho[2,1-	Andrographolides	Semisynthetic	Mpro<br>PLpro<br>S-protein<br>NSP15<br>RdRp	**Docking score (PyRx 0.8 tool)**<br>Mpro − 8.2 Kcal/mol<br>PLpro − 8.1 Kcal/mol<br>S-protein − 8.4 Kcal/mol<br>NSP15 − 8.1 Kcal/mol<br>RdRp − 8.5 Kcal/mol	[118]

	d][1,3]dioxin-7-yl)ethylidene)-4-hydroxydihydrofuran-2(3H)-one					
286.	(4S,E)-3-(2-((1R,3S,4aR,6aR,7R,10aR)-3-(4-chlorophenyl)-1,6a-dimethyl-8-methylenedecahydro-1H-naphtho[2,1-d][1,3]dioxin-7-yl)ethylidene)-4-hydroxydihydrofuran-2(3H)-one				**Docking score (PyRx 0.8 tool)**<br>Mpro − 7.9 Kcal/mol<br>PLpro − 7.8 Kcal/mol<br>S-protein − 8.7 Kcal/mol<br>NSP15 − 8.6 Kcal/mol<br>RdRp − 7.9 Kcal/mol	
287.	(+)-Usnic acid	Dibenzofuran compound	Mycosphaerella sp	Mpro	**Docking score (Glide)** −7.70 kcal/mol.<br>**ΔG (MM-GBSA)** –52.05 kcal/mol<br><br>**In vitro against the SARS-CoV-2 (KCDC,βCoV/ Korea / KCDC 2020) in vero cell**<br>**IC_{50}** 7.99 μM<br>**In vitro against the beta variant (B.1.351)**<br>**IC_{50}** 2.92 μM	[119]
288.	Sodium usnate (NaU)		*Mycosphaerella* sp		**In vitro against the SARS-CoV-2 (KCDC,βCoV/ Korea / KCDC 2020) in vero cell**<br>**IC_{50}** 5.33 μM	
289.	Cysteamine HCl	Thiol	Synthetic	S-protein	**In vitro against SARS-CoV-2 in Vero-TMPRSS2**<br>IC_{50}<br>Wild type (Washington) 1.252 mM<br>Alpha variant 1.528 mM<br>Beta variant 0.799 mM<br>Gamma variant 1.976 mM<br>Lambda variant 0.860 mM<br>Delta variant (subvariant 1) 1.066 mM<br>Delta variant (subvariant 2) 1.700 mM<br>Delta variant (subvariant 3) 1.373 mM<br>Omicron variant (subvariant 1) 0.671 mM<br>Omicron variant (subvariant 2) 1.006 mM	[120]
290.	Aurasperone A	Naphthopyrones	*Aspergillus niger*	Mpro	**Docking score (AutoDock Vina)** -8.1 kcal/mol<br>**ΔG (FEP)** -9.8 kcal/mol	[121]
291.	Batzelladines H	Alkaloid	***Marine sponge belonging to the genera*** *Batzella, Clathria and Monanchora*	Mpro	**Docking score (AutoDock Vina 1.2.2)** - 7.12 kcal/mol<br>**ΔG (MM-GBSA)** - 24.21 ± 6.32 kcal/mol<br>**In Vitro using Vero E6 cells infected with SARS-CoV-2 virus**<br>**IC_{50}** 12.25 μM	[122]
292.	5α-cholesta-4(27), 24-dien-3β, 23 β-diol	Steroid	*Ophiocoma Dentata Marine echinoderms*	Mpro<br>Nsp10<br>RdRp	**ΔG (MOE software)**<br>Mpro −24.68 kcal/mol<br>Nsp10 −23.47 kcal/mol<br>RdRp −29.86 kcal/mol<br><br>**% Inhibition at a concentration of 5 ng/μl (12.48 μM) =** 95%	[123]
293.	Ethyl *(2R, 3R, 4S, 5S)*-3''-((*E*)-*p*-methylbenzylidene)-2',4''-dioxo-4-(*p*-methylphenyl)-1''-((*S*)-1-phenylethyl) dispiro [indoline-3',2-pyrrolidine-3,5''-piperidine]-5-carboxylate	Pyrrolidine	Synthetic	Mpro	**ΔG (MMGPSA)** -35.2 kcal/mol	[124]
294.	Corilagin	Phenolic	*Euphorbia fischeriana*	Mpro	**Docking score (glide)** -7.643<br>**ΔG PrimeEnergy (MM/ GBSA)** -13412.45 kJ/mole	[125]

295.	Oxyresveratrol (HIT-2)	Phenolic	*Spirotropis longifolia*	Mpro	**Docking score (Glide)** -7.065<br>**ΔG PrimeEnergy (MM/ GBSA)** -13441.8 kJ/mole	
296.	Nephthoside monoacetate	Tetraprenyltoluquinol	*Nephthea chabrolii*	Mpro	**Docking score (Autodock Vina)** –8.8 kcal/mol	[126]
297.	Epirubicin	Anthracycline	*Synthetic*	Mpro	**Docking score (GOLD version 5.8)** 71.90<br>**ΔG (MM-GBSA)** -25.1 Kcal/mol	[8]
298.	Bortezomib	Pyrazine			**Docking score (GOLD version 5.8)** 67.26	
299.	3,5-Dicaffeoylquinic acid	Quinic acids	*Cressa cretica*	Mpro	**Docking score (Glide)** -6.375 kcal/mol<br>**ΔG (MM-GBSA)** -45.62 kcal/mol	[127]
300.	Theaflavin 3,3'digallate **(TF3)**	Theaflavin	*Camellia sinensis*	RdRp	**Docking score (smina server a fork of AutoDock Vina)** -14.92 kcal/mol<br>**ΔG (MMPBSA)** -93.59 ± 43.97 kJ/mol	[128]
301.	Theaflavin 3-gallate **(TF2a)**	Theaflavin		PLpro	**Docking score (smina server a fork of AutoDock Vina)** -10.90 kcal/mol<br>**ΔG (MMPBSA)** -96.88 ± 25.39 kJ/mol	
302.	Procyanidin B2	Flavonoids		Mpro	**Docking score (smina server a fork of AutoDock Vina)** -11.68 kcal/mol<br>**ΔG (MMPBSA)** -139.78 ± 16.51 kJ/mol	
303.	Lumacaftor	Pyridine	Synthetic	TMPRSS2	**Docking score (AutoDock Vina)** -8.9 kcal/mol<br>**ΔG (MM-PBSA) -**16.764 ± 3.553 kcal/mol	[129]
304.	Ergotamine	Alkaloid	*Claviceps Purpurea*	TMPRSS2	**Docking score (AutoDockVina)** -8.9 kcal/mol<br>**ΔG (MM-PBSA) -** 17.678 ±3.718 kcal/mol	
305.	N-(2-(2-acetyl-6-methoxy-1H-indol-3-yl)ethyl)butyramide	Indole	Synthetic	Mpro	**Docking score (AutoDockVina)** - 7.5 kcal/mol<br>**Docking score (AutoDock 4.2)** - 6.6 Kcal/ mol<br>**ΔG (MMGBSA)** -28 kcal/mol	[130]
306.	[=(3S,5S)-methyl 5-(hydroxymethyl)-3-(6-(4-methylpiperazin-1-yl)-9H-purin-9-yl)tetrahydrofuran-3-carboxylate]	Nucleoside analogue	Synthetic	RdRp	**Geometric shape complementarity score** (PatchDock server) 19.052	[131]
307.	Setomimycin	Tetrahydroanthracene	*Streptomyces* sp "Actinobacterium"	Mpro	**Docking score (Glide)** − 7.462 kcal/mol<br>**ΔG (MMGBSA)** − 71.864 kcal/mol<br>**In vitro against SARS-CoV-2 Mpro enzyme**<br>**IC_{50}** 12.02± 0.046 μM.	[132]
308.	Sinigrin	Polyphenol	*Isatis indigotica*	Mpro	**Docking score (AutoDock Vina)** -7.8 kcal/mol<br>**ΔG (MM-GBSA)** -41.75 kcal/mol	[133]
309.	Hesperetin				**Docking score (AutoDock Vina)** -7.9 kcal/mol<br>**ΔG (MM-GBSA)** -44.05 kcal/mol	
310.	Betamethasone	Steroid	Synthetic	Mpro	**Docking score (AutoDock Vina)** -7.8 kcal/mol<br>**ΔG (MM-GBSA)** -45.85 ± 1.48 kcal/mol	[134]
311.	Dexamethasone				**Docking score (AutoDock Vina)** -7.9 kcal/mol<br>**ΔG (MM-GBSA)** -42.76 ± 1.46 kcal/mol	
312.	Doxycycline	Tetracycline	Semisynthetic	RdRp	**Docking score (AutoDock Vina)** -7.3 Kcal/mol<br>**ΔG** (**MM-PBSA**) -163.980 kj/mol<br>**In vitro against IHUMI-3 strain**<br>**EC_{50}** 5.8 ± 1.6 μM<br>**EC_{90}** 21.7± 5.9 μM,	[135]
				E-protein	**Docking score (PyRx)** −9.1 kcal/mol	[36]
313.	Rhamnocitrin	Flavonoid	*Artemisia annua.*	Mpro	**Docking score (Glide)** −7.83 kcal/mol<br>**ΔG (MM-GBSA)** −49.53 kcal/mol	[136]

314.	Malabaricone C	Butanone	*Myristica fragrans* ***(Houtt.)***	Mpro	**Docking score (AutoDock Vina)** -5.9 kcal/mol<br>**In vitro against Mpro**<br>**IC_{50}** 319.17 uM	[137]
315.	Malabaricone B	Resorcinol		Mpro	**Docking score (AutoDock Vina)** -5.8 kcal/mol<br>**In vitro against Mpro**<br>**IC_{50}** 45.4 uM	
316.	Licarin A	Lignan		ACE2<br>Mpro	**Docking score (AutoDock Vina)**<br>ACE2 -8.7 kcal/mol<br>Mpro -8.2 kcal/mol<br>**In vitro against Mpro**<br>**IC_{50}** 668.63 nM<br>**In vitro against ACE2**<br>**IC_{50}** 3.59 uM	
317.	Licarin B			ACE2<br>Mpro	**Docking score (AutoDock Vina)**<br>ACE2 -9.7 kcal/mol<br>Mpro -7.9 kcal/mol<br>**In vitro against Mpro**<br>**IC_{50}** 558.99 nM<br>**In vitro against ACE2**<br>**IC_{50}** 430.11 nM	
318.	Licarin C	Benzofuran		ACE2<br>Mpro	**Docking score (AutoDock Vina)**<br>ACE2 -8.2 kcal/mol<br>Mpro -7.0 kcal/mol<br>**In vitro against Mpro**<br>**IC_{50}** 4.84 uM<br>**In vitro against ACE2**<br>**IC_{50}** 9.42 uM	
319.	Linagliptin	Piperidin	Synthetic	Mpro	**Docking score (AutoDock Vina)** –8.6 ± 0.55 kcal/mol<br>**ΔG (MMGBSA)** –31.83 ± 0.07 kcal/mol	[138]
320.	Caffeine	Alkaloid	*Theobroma cacao*		**Docking score (AutoDock Vina)** –5.6 ± 0.30 kcal/mol<br>**ΔG (MMGBSA) -**8.91 ± 0.02 kcal/mol	
321.	Mulberroside E	Phenols	*Morus alba*	Mpro	**Docking score (Auto dock Vina Wizard of PyRx software)** -7.0 kcal/mol<br>**ΔG (MM-PBSA) -**141.443 ± 9.313 kJ/mol	[139]
322.	Emblicanin A	Tannins	*Phyllanthus emblica*	RdRp	**Docking score (Auto dock Vina Wizard of PyRx software)** -9.2 kcal/mol<br>**ΔG (MM-PBSA) -**89.424 ± 3.130 kJ/mol	
323.	1-(4-hydroxyphenyl)-7-(3,4-dihydroxyphenyl)-1 6-heptadiene-3,5-dione	Diarylheptanoid	*Curcuma longa*	Mpro	**Docking score (Glide)** -12.779 kcal/mol<br>**ΔG (MM/GBSA)** -82.84 kcal/mol	[140]<br>[141]
324.	Tetrahydroxycurcumin	Diarylheptanoid		RdRp	**Docking score (Glide)** -9.289 kcal/mol<br>**ΔG (MM/GBSA) -**95.08 kcal/mol	
325.	4''-(4'''-hydroxyphenyl)-2''-oxo-3''-butenyl-3-(4'-hydroxyphenyl-3'-methoxy)-propenoate	Phenylpropene		S-protein	**Docking score (Glide)** -11.654 kcal/mol<br>**ΔG (MM/GBSA)** -81.34 kcal/mol	
326.	Tricin	Flavonoid	*Livistona decipiens Becc. (Arecaceae)*	Mpro	**Docking score (MOE 2019.01)** -6.7 kcal/mol	[142]
327.	Ferulic acid	Hydroxycinnamic acid	*Vitis amurensis*	Mpro	**Docking score(MOE 2019.01)** -5.4 Kcal/mol	[15]
				M-protein	**Docking score(PyRx)** −8.3 kcal/mol	[36]

328.	Naringenin	Flavonoid		Mpro	**Docking score(MOE 2019.01)** -7.8 Kcal/mol	[15]
329.	Catechin	Flavonoid			**Docking score(MOE 2019.01)** -7.9 Kcal/mol	
330.	Tert-butyl N-[4-[[5-chloro-4-[1-(4-methylphenyl)sulfonyl pyrrolo[2,3-b]pyridin-3-yl]pyrimidin-2-yl]amino]piperidin-1-yl]sulfonylcarbamate	Nitrogenous	*Rhazya stricta*	RBD of S-protein	**Docking score (AutoDock Vina)** − 8.9 Kcal/mol<br>**ΔG (MM-PBSA)** − 68.8 kJ/mol	[143]
331.	3-[[4-chloro-2,5-dioxo-1-(4-phenoxyphenyl)pyrrol-3-yl]amino]-N-(3,4-dichlorophenyl)benzamide				**Docking score (AutoDock Vina)** − 8.7 Kcal/mol<br>**ΔG (MM-PBSA)** − 71.6 kJ/mol	
332.	2,5-dichloro-N-(6-(4-((5-chloro-2-fluorophenyl)sulfonamido)phenyl)-4-oxo-4,5-dihydro-1H-pyrazolo[3,4-d]pyrimidin-3-yl)benzenesulfonamide				**Docking score (AutoDock Vina)** − 8.5 Kcal/mol<br>**ΔG (MM-PBSA)** − 74.9 kJ/mol	
333.	6,6'-(1-Methyl-1H-pyrrole-2,5-diyl)bis[1-acetyl-3-p-tolyl-1,6-dihydro-1,2,4-triazin-5(2H)-one]				**Docking score (AutoDock Vina)** − 8.5 Kcal/mol<br>**ΔG (MM-PBSA)** − 75.4 kJ/mol	
334.	N-[2,6-dichloro-4-[[3-[4-[(3,4-dimethyl-1,2-oxazol-5-yl)sulfamoyl]anilino]quinoxalin-2-yl]sulfamoyl]phenyl]acetamide				**Docking score (AutoDock Vina)** − 8.5 Kcal/mol<br>**ΔG (MM-PBSA)** − 60.9 kJ/mol	
335.	4-((3-ammonio-3-((R)-4-ammonio-5-(((R)-4,6-difluoro-2,3-dihydro-1H-inden-1-yl)amino)-5-oxopentyl)cyclobutyl)amino)-2-methylpyridin-1-ium	Pyrrolidin	Synthetic	TMPRSS2	**Docking score (Glide)** -14.982 kcal/mol<br><br>**ΔG (MM/GBSA)** -88.97 kcal/mol	[144]
336.	(1R,3S,4S)-3-((R)-3-ammonio-3-(4-(((1S,3S)-3-hydroxypyrrolidin-1-ium-1-yl)methyl)phenyl)propyl)-1-(4-(ammoniomethyl)benzyl)-4-hydroxypyrrolidin-1-ium	Pyrrolidin			**Docking score (Glide)** -14.758 kcal/mol<br>**ΔG (MM/GBSA)** -88.56 kcal/mol	
337.	(1R,3R,4S)-3-((S)-3-ammonio-6-(1,4-dihydro-3H-benzo[d][1,2]oxazin-3-yl)-6-oxohexyl)-1-(4-(ammoniomethyl)benzyl)-4-	Pyrrolidin			**Docking score (Glide)** -14.561 kcal/mol<br>**ΔG (MM/GBSA)** -87.97 kcal/mol	

	hydroxypyrrolidin-1-ium					
338.	Amentoflavoe	Flavonoid	*Torreya nucifera*	Mpro	**Docking score (AutoDock Vina)** -9.2 kcal/mol<br>**ΔG (MM/GBSA)** -59.57 ± 0.35 kcal/mol	[145]
339.	Bilobetin				**Docking score (AutoDock Vina)** -9.1 kcal/mol<br>**ΔG (MM/GBSA)** -66.31 ± 0.16 kcal/mol	
340.	Ginkgetin				**Docking score (AutoDock Vina)** -9.0 kcal/mol<br>**ΔG (MM/GBSA)** -63.62 ± 0.41 kcal/mol	
341.	(2E)-2-cyano-N-(1,5-dimethyl-3-oxo-2-phenyl-2,3-dihydro-1H-pyrazol-4-yl)-3-[5-(2-nitrophenyl)-2-furyl]acrylamide	Nitrogenous	Synthetic	Mpro	**Docking score (AutoDock Vina)** –9.2 kcal/mol<br>**Covalent docking affinity (Schrodinger program)** –6.088 kcal/mol<br>**ΔG (MM/GBSA)** –55.05 kcal/mol	[146]
342.	(E)-2-cyano-N-cyclopentyl-3-(2-(2-fluorophenoxy)-9-methyl-4-oxo-4H-pyrido[1,2-a]pyrimidin-3-yl)acrylamide	Nitrogenous	Synthetic		**Docking score (AutoDock Vina)** –8.6 kcal/mol<br>**Covalent docking affinity (Schrodinger program)** –6.665 kcal/mol<br>**ΔG (MM/GBSA)** –55.27 kcal/mol	
343.	(E)-3-(5-(4-fluorophenyl)furan-2-yl)-2-(1H-indole-3-carbonyl)acrylonitrile	Indol	Synthetic		**Docking score (AutoDock Vina)** –8.5 kcal/mol<br>**Covalent docking affinity (Schrodinger program)** –6.03 kcal/mol<br>**ΔG (MM/GBSA)** –40.73 kcal/mol	
344.	Uncarine F	Alkaloid	*Uncaria tomentosa*<br>Family Rubiaceae	ACE-2<br>RBD S-protein	**Docking score (AutoDockTools)**<br>ACE-2 –7.1 kcal/mol<br>RBD S-protein –6.1 kcal/mol	[147]
345.	Speciophylline	Alkaloid			**Docking score (AutoDockTools)**<br>ACE-2 -6.8 kcal/mol<br>RBD S-protein -6.8 kcal/mol	
346.	Uncaric acid	Terpene			**Docking score (AutoDockTools)**<br>ACE-2 -7.0 kcal/mol<br>RBD S-protein -5.5 kcal/mol	
347.	N-((S)-3-cyclohexyl-1-oxo-1-(((S)-1-oxo-3-((S)-2-oxopyrrolidin-3-yl)propan-2-yl)amino)propan-2-yl)-1H-indole-2-carboxamide<br>**(FB2001)**	Pyrrolidin	Synthetic	Mpro	**In vitro in Vero E6 cells against:**<br>**EC_{50}** :<br>**SARS-CoV-2** 0.42 ± 0.08 µM<br>**SARS-CoV-2 (Alpha)** 0.39 ± 0.01 µM<br>**SARS-CoV-2 (Beta)** 0.28 ± 0.11 µM<br>**SARS-CoV-2 (Delta)** 0.27 ± 0.05 µM<br>**SARS-CoV-2 (Omicron)** 0.26 ± 0.06 µM<br>**In vivo using K18-hACE2 mice(7–8 weeks) infected with SARS-CoV-2 delta**<br>**Lung viral loads:**<br>Day 2:<br>FB2001-100 mg/kg =1.02 log10 copies/g<br>FB2001-200 mg/kg = 1.60 log10 copies/g<br>Day 4:<br>FB2001-100 mg/kg = 0.67 log10 copies/g<br>FB2001-200 mg/kg = 1.14 log10 copies/g<br><br>**Brain viral loads**<br>Day 4<br>FB2001-100 mg/kg = 2.37 log10 copies/g<br>FB2001-200 mg/kg = 5.26 log10 copies/g<br><br>Effective dosage = 200 mg twice daily	[148]
348.	3-(2-((1-benzyl-5-chloro-1H-benzo[d]imidazol-2-yl)thio)-3-(furan-2-	Nitrogenous	Synthetic	Mpro	**In vitro against Mpro**<br>**IC_{50}** 14.9 µM	[149]

	yl)propanoyl)oxazolidin-2-one					
349.	1-(2-(o-tolylthio)phenyl)-4-(2-(trifluoromethyl)benzyl)piperazine	Nitrogenous	Synthetic	Mpro	**Docking results (AutoDock)** -8.02 kcal/mol	[150]
350.	Phenyl(4-(2-(0-tolylthio)phenyl)piperazin-1-yl)methanone				**Docking results (AutoDock)** -8.65 kcal/mol	
351.	(3-chloro-4-fluorophenyl)-4-(2-((2,4-dimethyl phenyl)<br>thio)phenyl)piperazine-1-carboxamide				**Docking results (AutoDock)** -9.26 kcal/mol	
352.	4-(2-((2,4-dimethylphenyl)thio)phenyl)-N-(pyridin-2-yl)<br>piperazine-1-carboxamide				**Docking results (AutoDock)** -8.59 kcal/mol	
353.	Nimotuzumab	Nitrogenous	Synthetic	EGFR blockade	A prospective, noncontrolled, multicenter phase I/II trial<br>On 41 patients (31 severe and 10 moderate)<br><br>Treatment consisted of nimotuzumab IV infusions over 2 h up to a maximum of 3 doses, every 72 h.<br>loading dose = 200 mg<br>subsequent doses =100 mg.<br><br>19.5% of the 41 patients needed mechanical ventilation after nimotuzumab.<br><br>**Recovery rates :**<br>80.6% for severe<br>90% for high-risk moderate patients	[151]
354.	Caffeic acid hexoside	Phenolic	*Sargassum wightii*	RBD of S-protein	**Docking score (PyRx software)**<br>caffeic acid hexoside -6.4 kcal/mol	[152]
355.	Phloretin	Phenolic			**Docking score (PyRx software)** -6.3 kcal/mol	
356.	Cholestan-3-ol, 2-methylene-, (3beta, 5 alpha) **(CMBA)**	Sterol	*Corallina officinalis*		**Docking score (PyRx software)** -6.0 kcal/mol	
357.	Paritaprevir	Nitrogenous	Synthetic	Adipose differentiation-related protein (ADRP)<br>Mpro	**Docking results (Autodock Vina v1.1.2 software)**<br>ADRP -36.2 kcal/mol<br>Mpro -32.2 kcal/mol<br>**ΔG (GBSA)**<br>ADRP -44.19 ± 0.61 kcal/mol	[153]
358.	Simeprevir	Nitrogenous	Synthetic	Mpro	**Docking results (Autodock Vina v1.1.2 software)**<br>-37.2 kcal/mol<br>**ΔG (GBSA)** -45.26 ± 0.16 kcal/mol	
				N-protein	**Docking score(PyRx)** −8.7 kcal/mol	[36]
359.	ZINC89341287	Terpene	*Astraeous hygrometricus*	Native Mpro	**Docking results(AutoDock Vina 1.1.2 software)** -7.7 kcal/mol<br><br>**ΔG(MM-PBSA)** - 83.738 kJ/mol	[154]
360.	ZINC12128321			Mutant Mpro	**Docking results(AutoDock Vina 1.1.2 software)** -7.3 kcal/mol<br>**ΔG(MM-PBSA)** -240.083 kJ/mol	

<table>
<tr><td>361.</td><td>Holyrine A</td><td>Alkaloid</td><td>Marine actinomycete</td><td rowspan="3"></td><td rowspan="3">In vitro against mNG-SARS-CoV-2 in Calu-3 cells
EC_{50}
Holyrine A 282 nM
Alotaketal C 106 nM
Bafilomycin D 38 nM

In vitro against SARS-CoV-2 Delta, Omicron BA.1, BA.2 and BA.5 infection in Calu-3 cells
EC_{50}
<table>
<tr><td></td><td>holyrine A</td><td>alotaketal C</td><td>bafilomycin D</td></tr>
<tr><td>SARS-CoV-2 Delta</td><td>Nuc 600 nM
dsRNA 1.0 µM</td><td>Nuc 4.9 µM
dsRNA 3.9 µM</td><td>Nuc 2.0 nM
dsRNA 50 nM</td></tr>
<tr><td>Omicron BA.1</td><td>Nuc 1.7 µM
dsRNA 1.6 µM</td><td>Nuc 700 nM
dsRNA 400 nM</td><td>Nuc 13.4 nM
dsRNA 67.3 nM</td></tr>
<tr><td>Omicron BA.2</td><td>Nuc 44 nM
dsRNA 394 nM</td><td>Nuc 700 nM
dsRNA700 nM</td><td>Nuc 4.6 nM
dsRNA 22 nM</td></tr>
<tr><td>Omicron BA.5</td><td>Nuc 80 nM
dsRNA 120 nM</td><td>Nuc 300 nM
dsRNA 700 nM</td><td>Nuc 43.9 nM
dsRNA 38.6 nM</td></tr>
</table>

Synergy scores of bafilomycin D and N-0385 using four reference models :
Loewe 26.5
ZIP 20.9
HAS , 26.4
Bliss 23</td><td rowspan="3">[155]</td></tr>
<tr><td>362.</td><td>Alotaketal C</td><td>Terpene</td><td>Phorbas sp.</td></tr>
<tr><td>363.</td><td>Bafilomycin D</td><td>Macrolide</td><td>Streptomyces sp</td></tr>
<tr><td>364.</td><td>(4S,12 aR)-N-(2,4-difluorobenzyl)-7-ethoxy-4- methyl-6,8-dioxo-3,4,6,8,12,12 a -hexahydro-2 H -pyrido[1´,2´:4,5]pyrazino[2,1- b][1,3]oxazine-9-carboxamide</td><td>Nitrogenous</td><td>Synthetic</td><td>Nsp16</td><td>Docking score (iGEM- DOCK version2.1 software)
-118.06 kcal/mol</td><td>[156]</td></tr>
<tr><td>365.</td><td>Neohesperidin</td><td>Flavonoid</td><td>Citrus aurantium</td><td>RBD S-protein of BA.2</td><td>Docking results (Autodock Vina) -7.8 kcal/mol
ΔG (MM-PBSA) 164.1 ± 17.5 kJ/mol</td><td>[157]</td></tr>
<tr><td>366.</td><td>1-(2,3-dihydrobenzo[b][1,4]dioxin-6-yl)-2-(furan-2-yl)-4,5-diphenyl-1 H -imidazole (DDFDI)</td><td>Imidazole derivative</td><td>Synthetic</td><td>RBD of S-protein

ADP ribose phosphatase of NSP3

Mpro

Nsp9</td><td>Docking score (Argus Lab 4.0)
6W41 -11.08 kcal/mol
ADP ribose phosphatase of NSP3 -9.75 kcal/mol
Mpro -10.62 kcal/mol
Nsp9 -12.25 kcal/mol</td><td>[158]</td></tr>
<tr><td>367.</td><td>Diethyl(4-hydroxyphenyl)[(pyridine-4-yl)amino]methylphosphonate</td><td>Pyridine</td><td>Synthetic</td><td>Mpro
RdRp</td><td>Docking score (Autodock 4.2.6)
Mpro −6.40 Kcal/mol
RdRp -8.40 Kcal/mol</td><td>[159]</td></tr>
<tr><td>368.</td><td>6-Amino-1-(4-chlorophenyl)-4-(2,4-dichlorophenyl)-3-methyl-1,4-dihydropyrano[2,3-c]pyrazole-5-carbonitrile</td><td>Nitrogenous</td><td>Synthetic</td><td>Mpro
and suppressing host cell cytokine release (TNF-α and IL-6)</td><td>In vitro against Mpro
IC_{50} 1.834 µM
In vitro against (SARS-CoV-2 isolate EGY/WAT-2 VACCERA) in Vero E6

% inhibttion at:
1 µM = 20.46 ± 0.03%
10 µM =91.23 ± 0.25%</td><td>[160]</td></tr>
</table>

					**In vivo**<br>Twelve Sprague Dawley Rat received 100 mg/kg of 27 compound using 100 µL carrageen to induce inflammation showing dropping in levels of TNF-α and IL-6 from :<br>587 to 385 pg/mL for TNF-α<br>82 to 64 pg/mL for IL-6<br><br>**Docking score (glide)** -16.459 Kcal/mol	
369.	2-Bromo- N ’-(2-((4-oxo-3,4-dihydroquinazolin-2-yl)thio)acetyl)benzohydrazide	Nitrogenous	Synthetic	Mpro	**In vitro against NRC-03-nhCoV virus in Vero-E6 cells**<br>**IC_{50}** 21.4 µM<br>**Docking score (MOE)** −12.45 Kcal/mol	[161]
370.	4-Nitro- N ’-(2-((4-oxo-3,4,5,6,7,8-hexahydroquinazolin-2-yl)thio)acetyl)benzohydrazide				**In vitro against NRC-03-nhCoV virus in Vero-E6 cells**<br>**IC_{50}** 38.45 µM<br>**Docking score (MOE)** −13.99 Kcal/mol	
371.	4-Chloro- N ’-(2-((4-oxo-3,4,5,6,7,8-hexahydroquinazolin-2-yl)thio)acetyl)benzohydrazide				**In vitro against NRC-03-nhCoV virus in Vero-E6 cells**<br>**IC_{50}** 26.4 µM<br>**Docking score (MOE)** −12.07 Kcal/mol	
372.	Thonzonium bromide	Nitrogenous	Synthetic	Mpro	**In vitro against Mpro**<br>**IC_{50}** 2.04 ± 0.25 µM	[162]
373.	Metformin	Nitrogenous	Synthetic	Activation of AMPK	**In vitro against SARS-CoV-2 strain B.1.1.8 (virus ID-hCoV-19/India/ TG-CCMB-L1021/2020; isolate; EPI_ISL_458046) in Calu3 and Caco2 cell lines**<br>**IC_{50}**<br>0.4 mM **in Calu3 cells**<br>1.43 mM **in Caco2 cells**	[163]
374.	4-(aminomethyl)-N-[4-methyl-3-[(4-pyridin-3-yl-1,3-thiazol-2-yl)amino]phenyl]benzamide **(CID10409602)**	Nitrogenous	Synthetic	Mpro	**Docking score (AutoDock4.2)** -10.50	[164]
375.	N-[4-methyl-3-[(4-pyridin-3-yl-1,3-thiazol-2-yl)amino] phenyl]-3-morpholin-4-ylbenzamide **(CID59789397)**	Nitrogenous			**Docking score (AutoDock4.2)** -10.10 kcal/mol	
376.	[2-methyl-5-[[4-(morpholin-4-ylmethyl)benzoyl]amino] phenyl]-(4-pyridin-3-yl-1,3-thiazol-2-yl)azanium **(CID143003625)**	Nitrogenous			**Docking score (AutoDock4.2)** -9.94 kcal/mol	
377.	Ensitrelvir	Nitrogenous	Synthetic	Mpro	**In vitro against SARS-CoV-2 Delta variant (lineage B.1.617.2)**<br>**EC_{50}**<br>**293 T-hACE2-TMPRSS2** 26 ± 6.65 nM<br>**Vero-TMPRSS2** 407 ± 21.3 nM<br>**Vero-TMPRSS2 with CP-100356** 69 ± 11.2 nM<br><br>**In vitro against**<br>**EC_{50}**<br>Alpha 46.0 ± 5.5 nM<br>Beta 35.1 ± 3.5 nM	[165]

					Gamma 61.7 ± 3.9 nM<br>Delta 34.8 ± 5.9 nM<br>Omicron/BA.1 23.9±3.6 nM<br><br>**In vivo** using Syrian hamsters infected with SARS-CoV-2 Delta, followed by oral administration of 200 mg/kg (b.i.d.) from 0 hpi resulted in a more than 105-fold decrease in viral RNA load, and the titers were under the detection limit in hamsters at 4 dpi	
378.	UKR1129266	Nitrogenous	Synthetic	PLpro	**Docking score (C-docker)** 32.27<br>**IC_{50}** 0.90.mu.M	[166]
379.	N-([1,1'-biphenyl]-4-yl)-N-((R)-2-oxo-2-(((S)-1-phenylethyl)amino)-1-(pyridin-3-yl)ethyl)furan-2-carboxamide	Nitrogenous	Synthetic	Mpro	**In vitro against Mpro**<br>**IC_{50}** 0.2 μM<br>**In vitro against SARS-CoV-2**<br>**EC_{50}**<br>**Vero E6** 1.27 μM<br>**Calu-3 cells** 3.03 μM	[167]
380.	N-([1,1'-biphenyl]-4-yl)-2,2-dichloro-N-((R)-2-oxo-2-(((S)-1-phenylethyl)amino)-1-(pyridin-3-yl)ethyl)acetamide	Nitrogenous			**In vitro against Mpro**<br>**IC_{50}** 0.43 μM<br>**In vitro against SARS-CoV-2**<br>**EC_{50}**<br>**Vero E6** 0.90 μM<br>**Caco2-hACE2** 2.05 μM<br>**Calu-3** 2.00 μM	
381.	LYSC69	Peptide	Synthetic	S-protein	**In vitro against SARS-CoV-2 pseudovirus in HEK293T-ACE2 cells**<br>**EC_{50}** 0.82 μM	[168]
382.	LYSC70				**In vitro against SARS-CoV-2 pseudovirus in HEK293T-ACE2 cells**<br>**EC_{50}** 0.91 μM	
383.	LYSC71				**In vitro against SARS-CoV-2 pseudovirus in HEK293T-ACE2 cells**<br>**EC_{50}** 2.67 μM	
384.	LYSC72				**In vitro against SARS-CoV-2 pseudovirus in HEK293T-ACE2 cells**<br>**EC_{50}** 2.52 μM	
385.	Withanolide	Sterol	*Athenaea velutina* (Solanaceae)	Mpro	**Docking score (Autodock 1.5.6)** -8.3 kcal/mol<br>**ΔG(MM/PBSA)** -135.566 ± 18.614 kJ/mol	[169]
				NSP16/10	**Docking score(AutoDock Vina)** –8.9 Kcal/mol<br>**ΔG (MM-PBSA)** –102.249 ± 26.084 kJ/mol	[170]
386.	5-(2-(4-Chlorobenzylidene) hydrazineyl)-N,N,3-trimethyl-4-(4-nitrophenyl)-4,9-dihydro-<br>1H-pyrazolo[4',3':5,6]pyrido[2,3-d]pyrimidin-7-amine	Nitrogenous	Synthetic	Mpro	**Docking score (Auto Dock Vina)** -8.4 Kcal/mol<br>**In vitro against against SARS-CoV-2 (Alpha strain, isolate hCoV-19/Egypt/NRC-3/2020 SARS-CoV-2 "NRC-03-nhCoV" virus) in Vero-E6 cells**<br>**IC_{50}** 1.2 μM	[171]
387.	5-(2-(4-Bromobenzylidene) hydrazineyl)-N,N,3-trimethyl-4-(4-nitrophenyl)-4,9-dihydro-1H-				**Docking score (Auto Dock Vina)**-8.3 Kcal/mol<br>**In vitro against against SARS-CoV-2 (Alpha strain, isolate hCoV-19/Egypt/NRC-3/2020 SARS-CoV-2 "NRC-03-nhCoV" virus) in Vero-E6 cells**<br>**IC_{50}** 2.34 μM	

	pyrazolo[4',3':5,6]pyrido[2,3-d]pyrimidin-7-amine				
388.	(E)-N,N,3-Trimethyl-5-(2-(4-methylbenzylidene)hydrazineyl)-4-(4-nitrophenyl)-4,9-dihydro-1H-pyrazolo[4',3':5,6]pyrido[2,3-d]pyrimidin-7-amine				**Docking score (Auto Dock Vina)**-8.5 Kcal/mol<br>**In vitro against against SARS-CoV-2 (Alpha strain, isolate hCoV-19/Egypt/NRC-3/2020 SARS-CoV-2 "NRC-03-nhCoV" virus) in Vero-E6 cells**<br>**IC_{50}** 2.3 µM	
389.	N-Propyl-3β-O-[2, 4-di-O-(α-l-rhamnopyranosyl)-β-d-glucopyranosyl]-lup-20 (29)-ene-28-oic acid	Saponin	Synthetic	S-protein	**In vitro against Omicron S2**<br>**K_d** 85.2 pM<br>**In vitro in 293 T-ACE2 cells against:**<br>**EC_{50}**<br>Omicron 4.66 ± 0.52 µM<br>Delta 4.25 ± 0.37 µM<br>N501Y 2.73 ± 0.31 µM<br>D614G 3.01 ± 0.25 µM<br>E484K 4.75 ± 0.58 µM<br>P681H 5.19 ± 0.86 µM	[172]
390.	P. tomentosa extract	-	*Paulownia tomentosa*	Mpro<br>S-protein	**In vitro against Viral isolate (BetaCov/Italy/CDG1/2020 |EPI ISL 412973| 2020–02–20) in Vero E6 cells**<br>**IC_{50}** 0.0035 mg/ml	[173]
391.	Dolutegravir	Nitrogenous	Synthetic	NSP16/10	**Docking score(AutoDock Vina)** –8.6 Kcal/mol<br>**ΔG (MM-PBSA) -**117.936 ± 16.505 kJ/mol	[170]
392.	Forsythoside A	<u>Hydroxycinnamic acid</u>	*Forsythia suspensa (Thunb.) Vahl*	Mpro	**In vitro against Mpro**<br>**IC_{50}** 22.26 µM	[16]
393.	Linoleic acid	Fatty acid			**In vitro against Mpro**<br>**IC_{50}** 47.09 µM.	
394.	Aurintricarboxylic acid	Quinomethanes	Synthetic	PLpro	**In vitro against PLpro**<br>**IC_{50}** 30 µM<br>**In vitro against SARS-CoV-2 in Vero E6 cells**<br>**IC_{50}** 50 µM<br>**In vivo using in Syrian hamster infected intra-nasally with SARS-CoV-2 every day for four days**<br>**Viral load from the oral swabs**<br>15 mg/kg (p= 0.0592)<br>30 mg/kg (p=0.0580)<br>and 45 mg/kg (p= 0.0439)	[174]
395.	Urtica dioica agglutinin	Lectin	*Urtica dioica* l. (Urticaceae)	RBD of S protein (RBD^{Omic})	**Docking score (CLUSPRO 2.0 web server)** −9.4 kcal/mol<br>**ΔG (MM-PBSA)** -87.201 +/- 9.589 kJ/mol	[175]
396.	Isoliensinine	Isoquinoline	*Nelumbo nucifera*	Mpro	**Docking score** -8.4 kcal/mol<br>**In vitro against Mpro**<br>**IC_{50}** 29.93 µM	[176]
397.	Cytinus hypocistis extract (CUAE)	Tannins rich extract	*Cytinus hypocistis*	Mpro	**In vitro against SARS-CoV-2 in vero Cells**<br>**EC_{50}** 2.34 µM/ml	[177]
398.	Tellimagrandin II	Ellagitannin			**Mean relative score (Autodock Vina)** 0.94<br>**ΔG MM-PBSA** -14.98 ± 11.656 kJ/mol	
399.	R. arboreum petals hot aqueous extract	-	*Rhododendron arboreum*	-	**In vitro in infected Vero E6 cells**<br>**%Inhibition at 1mg/ml** 80%<br>**IC_{50}** 173 µM /ml	[178]
400.	5-O-Feruloyl-quinic acid	Quinic acid		Mpro<br>ACE2	**Docking score (PyRx Virtual Screening software (version 0.8)**<br>Mpro -7.2 kcal/mol<br>ACE2 -8.6 kcal/mol<br>**ΔG (MM-GBSA)**<br>Mpro -38.54 kcal/mol<br>ACE2 -25.86 kcal/mol	

Table S2

n.	Compound	Study design	Outcome	Ref.
1.	Oral L-Arginine	A double-blind randomized trial in 169 patients receiving oral L-Arginine.	A reduction of respiratory support at 10 and 20 days.<br>A reduction of the time to hospital discharge.<br>A decrease in IL-2, IL-6, and IFN-γ, while increasing the levels of IL-10.	[179]
2.	Oral Favipiravir, Camostat and Ciclesonide combo versus monotherapy	An open-label, single-center phase 3 randomized clinical trial,<br>of 121 enrolled patients, 56 received monotherapy and 61 received combination therapy.	The median time to discharge was lower in the combination therapy vs monotherapy group.	[180]
3.	Saliravira®	A randomized, controlled, open-label clinical trial in outpatients treated for 10 days.	At day 8, mean rate of viral load was 50% lower compared to the control group ($p < 0.05$).<br>Out of 14 patients, 10 witnessed improved COVID-19 symptoms.<br>Mean treatment duration of intervened group was 4.9 days lower than the control group.<br>None of the treated group required hospitalization compared to the control group.	[181]
4.	Dexamethasone and Methylprednisolone	A randomized clinical trial in 143 patients under 80 years of age with moderate to severe COVID-19 were enrolled.<br>Patients were randomly assigned into two groups: dexamethasone (8 mg/day) and methylprednisolone (60 mg/day in two divided doses).	The duration of hospitalization was significantly (p <0.001) shorter in the dexamethasone group.<br>The duration of oxygen therapy in the dexamethasone group was significantly (p <0.001) shorter.	[182]
5.	Melatonin	Single-center, double-blind, randomized clinical trial including 74 hospitalized patients with mild to moderate COVID-19. Patients received Melatonin 3 mg three times daily for 14 days.	Cough, dyspnea, and fatigue, as well as the level of CRP and the pulmonary involvement had significantly improved (p <0.05).<br>The mean time of hospital discharge and recovery was significantly shorter (p <0.05).	[183]
6.	Nitazoxanide	A randomized double-blind placebo-controlled clinical trial of 379 patients; 184 treated with nitazoxanide at dose of 600 mg twice daily for five days.	Only 1 out of 184 (0.5%) patients in the treated group progressed to severe illness.<br>Compared to placebo, Nitazoxanide-treated participants	[184]

			experienced reductions in median TSR (3.1 days, $p = 0.09$) and recovery time (5.2 days, $p < 0{\cdot}01$).	
7.	*Nigella sativa* oil (NSO)	An open label randomized clinical trial in 173 patients with mild COVID-19 divided into control (87) or treatment groups (86), with the latter receiving 500 mg NSO (MARNYS® Cuminmar) twice daily for 10 days.	The percentage of recovered patients in NSO group was significantly high (p = 0.001).<br>The mean duration to recovery was shorter for patients receiving NSO ($p = 0.001$).	[185]
8.	Fluvoxamine	In TOGETHER randomized, placebo-controlled clinical trial, 741 patients administered 100 mg Fluvoxamine twice daily for 10 days and 756 patients were on placebo.	The absolute number of serious adverse events, Lower respiratory tract infections, hospital admissions in patients with confirmed COVID-19 and number of patients requiring mechanical ventilation associated with fluvoxamine was lower than for placebo.	[186]
9.	Giloy Ghanvati and Swasari Ras, Ashwagandha , Tulsi Ghanvati and Anu taila	A placebo controlled randomized double-blind pilot clinical trial.<br>Regimen: 1 g Tinospora cordifolia (Giloy Ghanvati), 2 g of traditional herbo-mineral formulation (Swasari Ras), 0.5 g of Withania somnifera (Ashwagandha), and 0.5 gm Ocimum sanctum (Tulsi Ghanvati) orally twice daily for 7 days.	71.1 % of the treatment group recovered versus 50.0 % of placebo-treated patients on the third day of treatment. At day 7, all the treatment group was recovered as compared to only 60.0 % in the placebo group.<br>Compared to placebo, the treatment combo reduced serum levels of hs-CRP, IL-6 and TNF-α by 12.4, 2.5 and 20 folds, respectively. The risk of delayed recovery among the treatment group was reduced by 40%.	[187]
10	Favipiravir	A randomized, open-label, parallel-arm, multicenter, phase 3 trial in 150 patients; 75 received favipiravir orally (day 1: 1800 mg BID and days 2–14: 800 mg BID).	Treatment reduced the median time of viral shedding and recovery to 5 and 3 days versus 7 and 5, respectively in the placebo.	[188]
11.	A standardized green propolis extract (EPP-AF®)	A randomized, controlled, open-label, single-center trial in 124 hospitalized adult COVID-19; 40 were assigned to EPP-AF® 400 mg/day, 42 to EPP-AF® 800 mg/day, and 42 to the control group.	The length of hospital stay post-intervention was shorter in both propolis groups than in the control group.<br>Propolis did not significantly affect the need for oxygen supplementation.<br>In the high dose propolis group, there was a lower rate of acute kidney injury than in the controls.	[189]

			No patient had propolis treatment discontinued due to adverse events.	
12.	Telmisartan	A parallel-group, randomized, two-arm, open-label, adaptive, multicenter superiority trial, receiving 80 mg twice daily for 14 days.	Telmisartan reduced the morbidity and mortality in hospitalized patients.	[190]
13.	Xuanfei Baidu Decoction (XBD) combined with conventional drug therapy	A pilot randomized clinical trial in 42 patients; 19 were randomly assigned to XBD (1 bag/time 200 ml, 2 times/day) plus conventional medicine while 20 patients only received conventional medicine for 1 week.	Clinical symptoms such as fever, cough, fatigue and loss of appetite were improved.<br>White blood cells and lymphocytes count returned to normal with a significant reduction of the C-reactive protein and erythrocyte sedimentation rate in the combo group versus those on conventional medicine.	[191]
14.	Tocilizumab	A non-controlled, prospective clinical trial in 76 patients with severe or critical SARS-CoV-2 infection, 42 patients received tocilizumab at a dose of 400 mg as a single dose via intravenous infusion.	Tocilizumab administration reduced the need for invasive ventilation to 14% and improved the clinical outcome in 83% of patients.	[192]
15.	Shenhuang Granule	An open-label, multicenter, randomized, controlled clinical trial,<br>54 patients were assigned to the control group and 57 to the SHG group (twice a day for 14 days).	Improvement in clinical outcomes in both severe and critical patients.<br>Administration of SHG in the severe category also reduced the rate of advancement to critical status and the percentage of patients receiving invasive ventilator therapy.	[193]
16.	Tixagevimab–cilgavimab	A phase 3, randomised, double-blind, placebo-controlled trial for non-hospitalised adults with mild to moderate COVID-19, receiving either a single tixagevimab–cilgavimab (n= 456) 600 mg dose or placebo (n=454).	Tixagevimab–cilgavimab group had statistically and clinically significant protection against progression to severe COVID-19 or death.	[194]
17.	Raloxifene	A phase 2 multicenter, randomized, placebo-controlled trial in adult patients with mild to moderate COVID-19 receving receive oral placebo (n=21), raloxifene 60 mg (n=24), or raloxifene 120 mg (n=23) by self-administration for a maximum of two weeks.	Raloxifene reduced the time of viral shedding in mild to moderate COVID-19 patients. Patients on 60 mg and 120 mg raloxifene didn't require a supplemental oxygen and/or mechanical ventilation.	[195]
18.	Heparin	Randomized controlled trials.	In moderately ill patients, heparin reduced death rate and the need for mechanical ventilation. However,	[196]

			heparin didn't benefit severely ill patients.	
19.	Ivermectin	A double-blind, randomized placebo-controlled trial.	In the ivermectin arm, a lower viral load and less viable cultures were seen compared to the controls.	[197]
20.	Remdesivir	A systematic review and meta-analysis of randomized controlled trials.	Remdesivir was associated with higher rates of hospital discharge, but it didn't show a significant reduction in mean time to clinical improvement or mortality.	[198]
21.	Azvudine (FNC)	A randomized, open label, controlled clinical trial on 20 Chinese patients.	The nucleic acid negative conversion (NANC) rate in FNC group was 100% after 4 days of treatment.	[199]
22.	Nimotuzumab	A prospective, noncontrolled, multicenter phase I/II trial on 41 patients (31 severe and 10 moderate) Nimotuzumab IV infusions were administered over 2 h up to a maximum of 3 doses, every 72 h (loading dose = 200 mg, subsequent doses =100 mg).	19.5% of nimotuzumab-treated patients required mechanical ventilation.<br>Recovery rates were 80.6% in severe cases and 90% in high-risk moderate patients.	[151]

references

1. Narkhede, R.R., et al., *Recognition of Natural Products as Potential Inhibitors of COVID-19 Main Protease (Mpro): In-Silico Evidences.* Nat Prod Bioprospect, 2020. **10**(5): p. 297-306.
2. van de Sand, L., et al., *Glycyrrhizin Effectively Inhibits SARS-CoV-2 Replication by Inhibiting the Viral Main Protease.* Viruses, 2021. **13**(4): p. 609.
3. Muhseen, Z.T., et al., *Computational Determination of Potential Multiprotein Targeting Natural Compounds for Rational Drug Design Against SARS-COV-2.* Molecules, 2021. **26**(3): p. 674.
4. Sinha, S.K., et al., *Identification of bioactive compounds from Glycyrrhiza glabra as possible inhibitor of SARS-CoV-2 spike glycoprotein and non-structural protein-15: a pharmacoinformatics study.* Journal of Biomolecular Structure and Dynamics, 2021. **39**(13): p. 4686-4700.
5. Fatima, S.W., S. Alam, and S.K. Khare, *Molecular and structural insights of β-boswellic acid and glycyrrhizic acid as potent SARS-CoV-2 Envelope protein inhibitors.* Phytomedicine Plus, 2022. **2**(2): p. 100241.
6. Vardhan, S. and S.K. Sahoo, *Computational studies on the interaction of SARS-CoV-2 Omicron SGp RBD with human receptor ACE2, limonin and glycyrrhizic acid.* Computers in Biology and Medicine, 2022. **144**: p. 105367.
7. Fu, L., et al., *Both Boceprevir and GC376 efficaciously inhibit SARS-CoV-2 by targeting its main protease.* Nat Commun, 2020. **11**(1): p. 4417.
8. Mandour, Y.M., D.P. Zlotos, and M. Alaraby Salem, *A multi-stage virtual screening of FDA-approved drugs reveals potential inhibitors of SARS-CoV-2 main protease.* Journal of Biomolecular Structure and Dynamics, 2022. **40**(5): p. 2327-2338.

9. Elzupir, A.O., *Inhibition of SARS-CoV-2 main protease 3CLpro by means of α-ketoamide and pyridone-containing pharmaceuticals using in silico molecular docking.* Journal of Molecular Structure, 2020. **1222**: p. 128878.
10. Zhang, L., et al., *Crystal structure of SARS-CoV-2 main protease provides a basis for design of improved α-ketoamide inhibitors.* Science, 2020. **368**(6489): p. 409-412.
11. Jin, Z., et al., *Structure of Mpro from SARS-CoV-2 and discovery of its inhibitors.* Nature, 2020. **582**(7811): p. 289-293.
12. Abian, O., et al., *Structural stability of SARS-CoV-2 3CLpro and identification of quercetin as an inhibitor by experimental screening.* International Journal of Biological Macromolecules, 2020. **164**: p. 1693-1703.
13. Shaldam, M.A., et al., *In silico screening of potent bioactive compounds from honeybee products against COVID-19 target enzymes.* Environmental Science and Pollution Research, 2021. **28**(30): p. 40507-40514.
14. Sharma, A., et al., *In-silico screening of plant-derived antivirals against main protease, 3CLpro and endoribonuclease, NSP15 proteins of SARS-CoV-2.* Journal of Biomolecular Structure and Dynamics, 2022. **40**(1): p. 86-100.
15. Souid, I., A. Korchef, and S. Souid, *In silico evaluation of Vitis amurensis Rupr. Polyphenol compounds for their inhibition potency against COVID-19 main enzymes Mpro and RdRp.* Saudi Pharmaceutical Journal, 2022. **30**(5): p. 570-584.
16. Jiang, H., et al., *Systematic identification of chemical components in Fufang Shuanghua oral liquid and screening of potential active components against SARS-CoV-2 protease.* Journal of Pharmaceutical and Biomedical Analysis, 2023. **223**: p. 115118.
17. Jo, S., et al., *Flavonoids with inhibitory activity against SARS-CoV-2 3CLpro.* Journal of Enzyme Inhibition and Medicinal Chemistry, 2020. **35**(1): p. 1539-1544.
18. Chandra, A., et al., *In silico identification and validation of natural antiviral compounds as potential inhibitors of SARS-CoV-2 methyltransferase.* Journal of Biomolecular Structure and Dynamics, 2022. **40**(14): p. 6534-6544.
19. Gurung, A.B., et al., *Unravelling lead antiviral phytochemicals for the inhibition of SARS-CoV-2 Mpro enzyme through in silico approach.* Life Sciences, 2020. **255**: p. 117831.
20. Kwon, P.S., et al., *Sulfated polysaccharides effectively inhibit SARS-CoV-2 in vitro.* Cell Discovery, 2020. **6**(1): p. 50.
21. Wang, M., et al., *Remdesivir and chloroquine effectively inhibit the recently emerged novel coronavirus (2019-nCoV) in vitro.* Cell Res, 2020. **30**(3): p. 269-271.
22. Choy, K.-T., et al., *Remdesivir, lopinavir, emetine, and homoharringtonine inhibit SARS-CoV-2 replication in vitro.* Antiviral Research, 2020. **178**: p. 104786.
23. Ramirez, S., et al., *Overcoming Culture Restriction for SARS-CoV-2 in Human Cells Facilitates the Screening of Compounds Inhibiting Viral Replication.* Antimicrob Agents Chemother, 2021. **65**(7): p. e0009721.
24. Chiu, W., et al., *Development and optimization of a high-throughput screening assay for in vitro anti-SARS-CoV-2 activity: Evaluation of 5676 Phase 1 Passed Structures.* J Med Virol, 2022. **94**(7): p. 3101-3111.
25. Tahir ul Qamar, M., et al., *Structural basis of SARS-CoV-2 3CLpro and anti-COVID-19 drug discovery from medicinal plants.* Journal of Pharmaceutical Analysis, 2020. **10**(4): p. 313-319.
26. Joshi, R.S., et al., *Discovery of potential multi-target-directed ligands by targeting host-specific SARS-CoV-2 structurally conserved main protease.* Journal of Biomolecular Structure and Dynamics, 2021. **39**(9): p. 3099-3114.

27. Beck, B.R., et al., *Predicting commercially available antiviral drugs that may act on the novel coronavirus (SARS-CoV-2) through a drug-target interaction deep learning model.* Computational and Structural Biotechnology Journal, 2020. **18**: p. 784-790.
28. Banerjee, S., et al., *Drug Repurposing to Identify Nilotinib as a Potential SARS-CoV-2 Main Protease Inhibitor: Insights from a Computational and In Vitro Study.* Journal of Chemical Information and Modeling, 2021. **61**(11): p. 5469-5483.
29. Coelho, C., et al., *Biochemical screening for SARS-CoV-2 main protease inhibitors.* PLOS ONE, 2020. **15**(10): p. e0240079.
30. Zahran, E.M., et al., *Bioactivity Potential of Marine Natural Products from Scleractinia-Associated Microbes and In Silico Anti-SARS-COV-2 Evaluation.* Mar Drugs, 2020. **18**(12).
31. Wang, J., *Fast Identification of Possible Drug Treatment of Coronavirus Disease-19 (COVID-19) through Computational Drug Repurposing Study.* Journal of Chemical Information and Modeling, 2020. **60**(6): p. 3277-3286.
32. Dai, W., et al., *Structure-based design of antiviral drug candidates targeting the SARS-CoV-2 main protease.* Science, 2020. **368**(6497): p. 1331-1335.
33. Benítez-Cardoza, C.G. and J.L. Vique-Sánchez, *Potential inhibitors of the interaction between ACE2 and SARS-CoV-2 (RBD), to develop a drug.* Life Sciences, 2020. **256**: p. 117970.
34. Vincent, S., et al., *Molecular Docking Studies on the Anti-viral Effects of Compounds From Kabasura Kudineer on SARS-CoV-2 3CLpro.* Frontiers in Molecular Biosciences, 2020. **7**.
35. Bharadwaj, S., et al., *Exploration of natural compounds with anti-SARS-CoV-2 activity via inhibition of SARS-CoV-2 Mpro.* Brief Bioinform, 2021. **22**(2): p. 1361-1377.
36. Bhowmik, D., et al., *Identification of potential inhibitors against SARS-CoV-2 by targeting proteins responsible for envelope formation and virion assembly using docking based virtual screening, and pharmacokinetics approaches.* Infection, Genetics and Evolution, 2020. **84**: p. 104451.
37. Latha, D., et al., *In silico, in vitro screening of plant extracts for anti-SARS-CoV-2 activity and evaluation of their acute and sub-acute toxicity.* Phytomedicine Plus, 2022. **2**(2): p. 100233.
38. Xiao, T., et al., *Myricetin Inhibits SARS-CoV-2 Viral Replication by Targeting Mpro and Ameliorates Pulmonary Inflammation.* Frontiers in Pharmacology, 2021. **12**.
39. M, P., et al., *Unravelling high-affinity binding compounds towards transmembrane protease serine 2 enzyme in treating SARS-CoV-2 infection using molecular modelling and docking studies.* European Journal of Pharmacology, 2021. **890**: p. 173688.
40. Liu, H., et al., *Scutellaria baicalensis extract and baicalein inhibit replication of SARS-CoV-2 and its 3C-like protease in vitro.* Journal of Enzyme Inhibition and Medicinal Chemistry, 2021. **36**(1): p. 497-503.
41. Basu, S., et al., *Novel cyclohexanone compound as a potential ligand against SARS-CoV-2 main-protease.* Microbial Pathogenesis, 2020. **149**: p. 104546.
42. Haribabu, J., et al., *Synthesis of Palladium(II) Complexes via Michael Addition: Antiproliferative Effects through ROS-Mediated Mitochondrial Apoptosis and Docking with SARS-CoV-2.* Inorganic Chemistry, 2020. **59**(23): p. 17109-17122.
43. Vaidya, N.A. and R. Vyas, *Computational Studies of Hydroxychloroquine and Chloroquine Metabolites as Possible Candidates for Coronavirus (COVID-19) Treatment.* Frontiers in Pharmacology, 2020. **11**.
44. Yañez, O., et al., *In Silico Study of Coumarins and Quinolines Derivatives as Potent Inhibitors of SARS-CoV-2 Main Protease.* Frontiers in Chemistry, 2021. **8**.
45. Sinha, M., et al., *Analogue discovery of safer alternatives to HCQ and CQ drugs for SAR-CoV-2 by computational design.* Computers in Biology and Medicine, 2021. **130**: p. 104222.

46. Chidambaram, S., et al., *Synthesis of novel coumarin analogues: Investigation of molecular docking interaction of SARS-CoV-2 proteins with natural and synthetic coumarin analogues and their pharmacokinetics studies.* Saudi Journal of Biological Sciences, 2021. **28**(1): p. 1100-1108.
47. Rajagopal, K., et al., *Identification of some novel oxazine substituted 9-anilinoacridines as SARS-CoV-2 inhibitors for COVID-19 by molecular docking, free energy calculation and molecular dynamics studies.* Journal of Biomolecular Structure and Dynamics, 2021. **39**(15): p. 5551-5562.
48. Khater, S., et al., *Combining SARS-CoV-2 Proofreading Exonuclease and RNA-Dependent RNA Polymerase Inhibitors as a Strategy to Combat COVID-19: A High-Throughput in silico Screening.* Frontiers in Microbiology, 2021. **12**.
49. Almalki, S.A., et al., *Synthesis and characterization of new thiazole-based Co(II) and Cu(II) complexes; therapeutic function of thiazole towards COVID-19 in comparing to current antivirals in treatment protocol.* Journal of Molecular Structure, 2021. **1244**: p. 130961.
50. Dhanjal, J.K., et al., *Molecular mechanism of anti-SARS-CoV2 activity of Ashwagandha-derived withanolides.* International Journal of Biological Macromolecules, 2021. **184**: p. 297-312.
51. Yalçın, S., S. Yalçınkaya, and F. Ercan, *Determination of Potential Drug Candidate Molecules of the Hypericum perforatum for COVID-19 Treatment.* Current Pharmacology Reports, 2021. **7**(2): p. 42-48.
52. Malik, A., et al., *Inhibitory Potential of Phytochemicals on Interleukin-6-Mediated T-Cell Reduction in COVID-19 Patients: A Computational Approach.* Bioinformatics and Biology Insights, 2021. **15**: p. 11779322211021430.
53. Ibrahim, M.A.A., et al., *Blue Biotechnology: Computational Screening of Sarcophyton Cembranoid Diterpenes for SARS-CoV-2 Main Protease Inhibition.* Marine Drugs, 2021. **19**(7): p. 391.
54. Panikar, S., et al., *Essential oils as an effective alternative for the treatment of COVID-19: Molecular interaction analysis of protease (Mpro) with pharmacokinetics and toxicological properties.* Journal of Infection and Public Health, 2021. **14**(5): p. 601-610.
55. Nallusamy, S., et al., *Exploring Phytochemicals of Traditional Medicinal Plants Exhibiting Inhibitory Activity Against Main Protease, Spike Glycoprotein, RNA-dependent RNA Polymerase and Non-Structural Proteins of SARS-CoV-2 Through Virtual Screening.* Frontiers in Pharmacology, 2021. **12**.
56. Kashyap, T., et al., *Selinexor, a novel selective inhibitor of nuclear export, reduces SARS-CoV-2 infection and protects the respiratory system in vivo.* Antiviral Research, 2021. **192**: p. 105115.
57. Tripathi, M.K., et al., *Identification of bioactive molecule from Withania somnifera (Ashwagandha) as SARS-CoV-2 main protease inhibitor.* Journal of Biomolecular Structure and Dynamics, 2021. **39**(15): p. 5668-5681.
58. Shree, P., et al., *Targeting COVID-19 (SARS-CoV-2) main protease through active phytochemicals of ayurvedic medicinal plants - Withania somnifera (Ashwagandha), Tinospora cordifolia (Giloy) and Ocimum sanctum (Tulsi) - a molecular docking study.* J Biomol Struct Dyn, 2022. **40**(1): p. 190-203.
59. Zarezade, V., et al., *The identification of novel inhibitors of human angiotensin-converting enzyme 2 and main protease of Sars-Cov-2: A combination of in silico methods for treatment of COVID-19.* Journal of Molecular Structure, 2021. **1237**: p. 130409.
60. Falade, V.A., et al., *In silico investigation of saponins and tannins as potential inhibitors of SARS-CoV-2 main protease (M(pro)).* In Silico Pharmacol, 2021. **9**(1): p. 9.
61. Girgis, A.S., et al., *3-Alkenyl-2-oxindoles: Synthesis, antiproliferative and antiviral properties against SARS-CoV-2.* Bioorganic Chemistry, 2021. **114**: p. 105131.

62. Ghosh, R., et al., *Evaluation of green tea polyphenols as novel corona virus (SARS CoV-2) main protease (Mpro) inhibitors – an in silico docking and molecular dynamics simulation study.* Journal of Biomolecular Structure and Dynamics, 2021. **39**(12): p. 4362-4374.
63. Orfali, R., et al., *Sinapic Acid Suppresses SARS CoV-2 Replication by Targeting Its Envelope Protein.* Antibiotics, 2021. **10**(4): p. 420.
64. Huang, X., et al., *Identification of 13 Guanidinobenzoyl- or Aminidinobenzoyl-Containing Drugs to Potentially Inhibit TMPRSS2 for COVID-19 Treatment.* International Journal of Molecular Sciences, 2021. **22**(13): p. 7060.
65. Elzupir, A.O., *Molecular Docking and Dynamics Investigations for Identifying Potential Inhibitors of the 3-Chymotrypsin-like Protease of SARS-CoV-2: Repurposing of Approved Pyrimidonic Pharmaceuticals for COVID-19 Treatment.* Molecules, 2021. **26**(24): p. 7458.
66. Abdelmohsen, U.R., et al., *Natural coumarins as potential anti-SARS-CoV-2 agents supported by docking analysis.* RSC Advances, 2021. **11**(28): p. 16970-16979.
67. ter Ellen, B.M., et al., *Resveratrol and Pterostilbene Inhibit SARS-CoV-2 Replication in Air–Liquid Interface Cultured Human Primary Bronchial Epithelial Cells.* Viruses, 2021. **13**(7): p. 1335.
68. Samarth, N., R. Kabra, and S. Singh, *Anthraquinolone and quinolizine derivatives as an alley of future treatment for COVID-19: an in silico machine learning hypothesis.* Scientific Reports, 2021. **11**(1): p. 17915.
69. Soltane, R., et al., *Strong Inhibitory Activity and Action Modes of Synthetic Maslinic Acid Derivative on Highly Pathogenic Coronaviruses: COVID-19 Drug Candidate.* Pathogens, 2021. **10**(5): p. 623.
70. Alhadrami, H.A., et al., *Cnicin as an Anti-SARS-CoV-2: An Integrated In Silico and In Vitro Approach for the Rapid Identification of Potential COVID-19 Therapeutics.* Antibiotics, 2021. **10**(5): p. 542.
71. Tito, A., et al., *Pomegranate Peel Extract as an Inhibitor of SARS-CoV-2 Spike Binding to Human ACE2 Receptor (in vitro): A Promising Source of Novel Antiviral Drugs.* Frontiers in Chemistry, 2021. **9**.
72. Kandeel, M., et al., *Repurposing of FDA-approved antivirals, antibiotics, anthelmintics, antioxidants, and cell protectives against SARS-CoV-2 papain-like protease.* J Biomol Struct Dyn, 2021. **39**(14): p. 5129-5136.
73. Naidoo, D., et al., *Cyanobacterial metabolites as promising drug leads against the M(pro) and PL(pro) of SARS-CoV-2: an in silico analysis.* J Biomol Struct Dyn, 2021. **39**(16): p. 6218-6230.
74. Wahl, A., et al., *SARS-CoV-2 infection is effectively treated and prevented by EIDD-2801.* Nature, 2021. **591**(7850): p. 451-457.
75. Li, P., et al., *SARS-CoV-2 Omicron variant is highly sensitive to molnupiravir, nirmatrelvir, and the combination.* Cell Research, 2022. **32**(3): p. 322-324.
76. Johnson, D.M., et al., *Evaluation of molnupiravir (EIDD-2801) efficacy against SARS-CoV-2 in the rhesus macaque model.* Antiviral Res, 2023. **209**: p. 105492.
77. Zígolo, M.A., et al., *Virtual screening of plant-derived compounds against SARS-CoV-2 viral proteins using computational tools.* Science of The Total Environment, 2021. **781**: p. 146400.
78. Lim, C.T., et al., *Identifying SARS-CoV-2 antiviral compounds by screening for small molecule inhibitors of Nsp3 papain-like protease.* Biochem J, 2021. **478**(13): p. 2517-2531.
79. Zhao, Y., et al., *High-throughput screening identifies established drugs as SARS-CoV-2 PLpro inhibitors.* Protein Cell, 2021. **12**(11): p. 877-888.
80. Gangadevi, S., et al., *Kobophenol A Inhibits Binding of Host ACE2 Receptor with Spike RBD Domain of SARS-CoV-2, a Lead Compound for Blocking COVID-19.* The Journal of Physical Chemistry Letters, 2021. **12**(7): p. 1793-1802.

81. Qiao, J., et al., *SARS-CoV-2 M(pro) inhibitors with antiviral activity in a transgenic mouse model.* Science, 2021. **371**(6536): p. 1374-1378.
82. Forrestall, K.L., et al., *2-Pyridone natural products as inhibitors of SARS-CoV-2 main protease.* Chemico-Biological Interactions, 2021. **335**: p. 109348.
83. Fakhar, Z., et al., *Anthocyanin derivatives as potent inhibitors of SARS-CoV-2 main protease: An in-silico perspective of therapeutic targets against COVID-19 pandemic.* J Biomol Struct Dyn, 2021. **39**(16): p. 6171-6183.
84. Ghosh, R., et al., *Identification of polyphenols from Broussonetia papyrifera as SARS CoV-2 main protease inhibitors using in silico docking and molecular dynamics simulation approaches.* J Biomol Struct Dyn, 2021. **39**(17): p. 6747-6760.
85. Amporndanai, K., et al., *Inhibition mechanism of SARS-CoV-2 main protease by ebselen and its derivatives.* Nat Commun, 2021. **12**(1): p. 3061.
86. Abdelli, I., et al., *In silico study the inhibition of angiotensin converting enzyme 2 receptor of COVID-19 by Ammoides verticillata components harvested from Western Algeria.* J Biomol Struct Dyn, 2021. **39**(9): p. 3263-3276.
87. Wen, L., et al., *In silico structure-based discovery of a SARS-CoV-2 main protease inhibitor.* Int J Biol Sci, 2021. **17**(6): p. 1555-1564.
88. Aanouz, I., et al., *Moroccan Medicinal plants as inhibitors against SARS-CoV-2 main protease: Computational investigations.* J Biomol Struct Dyn, 2021. **39**(8): p. 2971-2979.
89. Kumar, A., et al., *Inhibition Potencies of Phytochemicals Derived from Sesame Against SARS-CoV-2 Main Protease: A Molecular Docking and Simulation Study.* Front Chem, 2021. **9**: p. 744376.
90. Rolta, R., et al., *In silico screening of hundred phytocompounds of ten medicinal plants as potential inhibitors of nucleocapsid phosphoprotein of COVID-19: an approach to prevent virus assembly.* J Biomol Struct Dyn, 2021. **39**(18): p. 7017-7034.
91. Thurakkal, L., et al., *An in-silico study on selected organosulfur compounds as potential drugs for SARS-CoV-2 infection via binding multiple drug targets.* Chemical Physics Letters, 2021. **763**: p. 138193.
92. Havranek, B. and S.M. Islam, *An in silico approach for identification of novel inhibitors as potential therapeutics targeting COVID-19 main protease.* Journal of Biomolecular Structure and Dynamics, 2021. **39**(12): p. 4304-4315.
93. Yang, M., et al., *Resveratrol inhibits the replication of severe acute respiratory syndrome coronavirus 2 (SARS-CoV-2) in cultured Vero cells.* Phytother Res, 2021. **35**(3): p. 1127-1129.
94. Wahedi, H.M., S. Ahmad, and S.W. Abbasi, *Stilbene-based natural compounds as promising drug candidates against COVID-19.* Journal of Biomolecular Structure and Dynamics, 2021. **39**(9): p. 3225-3234.
95. Du, A., et al., *Epigallocatechin-3-gallate, an active ingredient of Traditional Chinese Medicines, inhibits the 3CLpro activity of SARS-CoV-2.* Int J Biol Macromol, 2021. **176**: p. 1-12.
96. Rivero-Segura, N.A. and J.C. Gomez-Verjan, *In Silico Screening of Natural Products Isolated from Mexican Herbal Medicines against COVID-19.* Biomolecules, 2021. **11**(2).
97. Gyebi, G.A., et al., *Potential inhibitors of coronavirus 3-chymotrypsin-like protease (3CL(pro)): an in silico screening of alkaloids and terpenoids from African medicinal plants.* J Biomol Struct Dyn, 2021. **39**(9): p. 3396-3408.
98. Kundu, D., et al., *Identification of new anti-nCoV drug chemical compounds from Indian spices exploiting SARS-CoV-2 main protease as target.* J Biomol Struct Dyn, 2021. **39**(9): p. 3428-3434.
99. Hisham Shady, N., et al. *Sterols and Triterpenes: Antiviral Potential Supported by In-Silico Analysis*. Plants, 2021. **10**, DOI: 10.3390/plants10010041.
100. Owen, D.R., et al., *An oral SARS-CoV-2 M(pro) inhibitor clinical candidate for the treatment of COVID-19.* Science, 2021. **374**(6575): p. 1586-1593.

101. Abdelnabi, R., et al., *The oral protease inhibitor (PF-07321332) protects Syrian hamsters against infection with SARS-CoV-2 variants of concern.* Nature Communications, 2022. **13**(1): p. 719.
102. Wang, G., et al., *Dalbavancin binds ACE2 to block its interaction with SARS-CoV-2 spike protein and is effective in inhibiting SARS-CoV-2 infection in animal models.* Cell Research, 2021. **31**(1): p. 17-24.
103. Srivastava, N., et al., *A molecular dynamics simulation study of the ACE2 receptor with screened natural inhibitors to identify novel drug candidate against COVID-19.* PeerJ, 2021. **9**: p. e11171.
104. Oso, B.J., A.O. Adeoye, and I.F. Olaoye, *Pharmacoinformatics and hypothetical studies on allicin, curcumin, and gingerol as potential candidates against COVID-19-associated proteases.* J Biomol Struct Dyn, 2022. **40**(1): p. 389-400.
105. Farhat, A., et al., *Apigenin analogues as SARS-CoV-2 main protease inhibitors: In-silico screening approach.* Bioengineered, 2022. **13**(2): p. 3350-3361.
106. Yi, Y., et al., *Schaftoside inhibits 3CLpro and PLpro of SARS-CoV-2 virus and regulates immune response and inflammation of host cells for the treatment of COVID-19.* Acta Pharmaceutica Sinica B, 2022. **12**(11): p. 4154-4164.
107. Yi, Y., et al., *Natural triterpenoids from licorice potently inhibit SARS-CoV-2 infection.* Journal of Advanced Research, 2022. **36**: p. 201-210.
108. Hamdy, R., et al., *Comparative evaluation of flavonoids reveals the superiority and promising inhibition activity of silibinin against SARS-CoV-2.* Phytother Res, 2022. **36**(7): p. 2921-2939.
109. Jin, Y.-H., et al., *Natural Polyphenols, 1,2,3,4,6-O-Pentagalloyglucose and Proanthocyanidins, as Broad-Spectrum Anticoronaviral Inhibitors Targeting Mpro and RdRp of SARS-CoV-2.* Biomedicines, 2022. **10**(5): p. 1170.
110. Schäfer, A., et al., *Therapeutic treatment with an oral prodrug of the remdesivir parental nucleoside is protective against SARS-CoV-2 pathogenesis in mice.* Sci Transl Med, 2022. **14**(643): p. eabm3410.
111. Junior, N.N., et al., *In silico evaluation of lapachol derivatives binding to the Nsp9 of SARS-CoV-2.* J Biomol Struct Dyn, 2022. **40**(13): p. 5917-5931.
112. Rout, J., B.C. Swain, and U. Tripathy, *In silico investigation of spice molecules as potent inhibitor of SARS-CoV-2.* J Biomol Struct Dyn, 2022. **40**(2): p. 860-874.
113. Majumder, R. and M. Mandal, *Screening of plant-based natural compounds as a potential COVID-19 main protease inhibitor: an in silico docking and molecular dynamics simulation approach.* J Biomol Struct Dyn, 2022. **40**(2): p. 696-711.
114. Rakshit, G., et al., *Flavonoids as potential therapeutics against novel coronavirus disease-2019 (nCOVID-19).* J Biomol Struct Dyn, 2022. **40**(15): p. 6989-7001.
115. Wang, Q., et al., *Virtual screening of approved clinic drugs with main protease (3CL(pro)) reveals potential inhibitory effects on SARS-CoV-2.* J Biomol Struct Dyn, 2022. **40**(2): p. 685-695.
116. Chakraborty, R., et al., *In-silico screening and in-vitro assay show the antiviral effect of Indomethacin against SARS-CoV-2.* Computers in Biology and Medicine, 2022. **147**: p. 105788.
117. Chan, J.F., et al., *A molecularly engineered, broad-spectrum anti-coronavirus lectin inhibits SARS-CoV-2 and MERS-CoV infection in vivo.* Cell Rep Med, 2022. **3**(10): p. 100774.
118. Veerasamy, R. and R. Karunakaran, *Molecular docking unveils the potential of andrographolide derivatives against COVID-19: an in silico approach.* Journal of Genetic Engineering and Biotechnology, 2022. **20**(1): p. 58.
119. Oh, E., et al., *(+)-Usnic acid and its salts, inhibitors of SARS-CoV-2, identified by using in silico methods and in vitro assay.* Scientific Reports, 2022. **12**(1): p. 13118.
120. Thoene, J., et al., *In vitro activity of cysteamine against SARS-CoV-2 variants.* Molecular Genetics and Metabolism, 2022. **137**(1): p. 192-200.

121. ElNaggar, M.H., et al., *Aurasperone A Inhibits SARS CoV-2 In Vitro: An Integrated In Vitro and In Silico Study.* Marine Drugs, 2022. **20**(3): p. 179.
122. Elgohary, A.M., et al., *Investigating the structure-activity relationship of marine polycyclic batzelladine alkaloids as promising inhibitors for SARS-CoV-2 main protease (Mpro).* Computers in Biology and Medicine, 2022. **147**: p. 105738.
123. Abd El Hafez, M.S.M., et al., *Characterization, in-silico, and in-vitro study of a new steroid derivative from Ophiocoma dentata as a potential treatment for COVID-19.* Scientific Reports, 2022. **12**(1): p. 5846.
124. Toumi, A., et al., *Design of Novel Enantiopure Dispirooxindolopyrrolidine-Piperidones as Promising Candidates toward COVID-19: Asymmetric Synthesis, Crystal Structure and In Silico Studies.* Molecules, 2022. **27**(12): p. 3945.
125. Aleissa, M.S., et al., *Screening, molecular simulation & in silico kinetics of virtually designed covid-19 main protease inhibitors.* Journal of King Saud University - Science, 2022. **34**(8): p. 102283.
126. Abdelhafez, O.H., et al., *Natural metabolites from the soft coral Nephthea sp. as potential SARS-CoV-2 main protease inhibitors.* Natural Product Research, 2022. **36**(11): p. 2893-2896.
127. Shah, S., et al., *Prospecting for Cressa cretica to treat COVID-19 via in silico molecular docking models of the SARS-CoV-2.* Journal of Biomolecular Structure and Dynamics, 2022. **40**(12): p. 5643-5652.
128. Gogoi, M., et al., *Black tea bioactives as inhibitors of multiple targets of SARS-CoV-2 (3CLpro, PLpro and RdRp): a virtual screening and molecular dynamic simulation study.* Journal of Biomolecular Structure and Dynamics, 2022. **40**(15): p. 7143-7166.
129. Wang, S., X. Fang, and Y. Wang, *In Silico Screening of Novel TMPRSS2 Inhibitors for Treatment of COVID-19.* Molecules, 2022. **27**(13): p. 4210.
130. Hassam, M., et al., *Identification of potent compounds against SARs-CoV-2: An in-silico based drug searching against Mpro.* Computers in Biology and Medicine, 2022. **151**: p. 106284.
131. Defant, A., et al., *Synthesis of Nucleoside-like Molecules from a Pyrolysis Product of Cellulose and Their Computational Prediction as Potential SARS-CoV-2 RNA-Dependent RNA Polymerase Inhibitors.* International Journal of Molecular Sciences, 2022. **23**(1): p. 518.
132. Manhas, R.S., et al., *Setomimycin as a potential molecule for COVID-19 target: in silico approach and in vitro validation.* Molecular Diversity, 2022.
133. Ghosh, R., et al., *Depicting the inhibitory potential of polyphenols from Isatis indigotica root against the main protease of SARS CoV-2 using computational approaches.* Journal of Biomolecular Structure and Dynamics, 2022. **40**(9): p. 4110-4121.
134. Ghosh, R., et al., *Potential therapeutic use of corticosteroids as SARS CoV-2 main protease inhibitors: a computational study.* Journal of Biomolecular Structure and Dynamics, 2022. **40**(5): p. 2053-2066.
135. Alexpandi, R., et al., *Repurposing of Doxycycline to Hinder the Viral Replication of SARS-CoV-2: From in silico to in vitro Validation.* Frontiers in Microbiology, 2022. **13**.
136. Johnson, T.O., et al., *A Computational Approach to Elucidate the Interactions of Chemicals From Artemisia annua Targeted Toward SARS-CoV-2 Main Protease Inhibition for COVID-19 Treatment.* Frontiers in Medicine, 2022. **9**.
137. Ongtanasup, T., et al., *In silico investigation of ACE2 and the main protease of SARS-CoV-2 with phytochemicals from Myristica fragrans (Houtt.) for the discovery of a novel COVID-19 drug.* Saudi Journal of Biological Sciences, 2022. **29**(9): p. 103389.
138. Elzupir, A.O., *Caffeine and caffeine-containing pharmaceuticals as promising inhibitors for 3-chymotrypsin-like protease of SARS-CoV-2.* Journal of Biomolecular Structure and Dynamics, 2022. **40**(5): p. 2113-2120.

139. Sharma, A., et al., *Identification of natural inhibitors against prime targets of SARS-CoV-2 using molecular docking, molecular dynamics simulation and MM-PBSA approaches.* Journal of Biomolecular Structure and Dynamics, 2022. **40**(7): p. 3296-3311.
140. Emirik, M., *Potential therapeutic effect of turmeric contents against SARS-CoV-2 compared with experimental COVID-19 therapies: in silico study.* Journal of Biomolecular Structure and Dynamics, 2022. **40**(5): p. 2024-2037.
141. Li, S., *Chemical Composition and Product Quality Control of Turmeric (Curcuma longa L.).* Pharmaceutical Crops, 2011. **5**: p. 28-54.
142. El-hawary, S.S., et al., *Secondary metabolites of Livistona decipiens as potential inhibitors of SARS-CoV-2.* RSC Advances, 2022. **12**(30): p. 19505-19511.
143. Baeshen, N.A., et al., *In silico screening of some compounds derived from the desert medicinal plant Rhazya stricta for the potential treatment of COVID-19.* Scientific Reports, 2022. **12**(1): p. 11120.
144. Alzain, A.A., F.A. Elbadwi, and F.O. Alsamani, *Discovery of novel TMPRSS2 inhibitors for COVID-19 using in silico fragment-based drug design, molecular docking, molecular dynamics, and quantum mechanics studies.* Informatics in Medicine Unlocked, 2022. **29**: p. 100870.
145. Ghosh, R., et al., *Computer aided identification of potential SARS CoV-2 main protease inhibitors from diterpenoids and biflavonoids of Torreya nucifera leaves.* Journal of Biomolecular Structure and Dynamics, 2022. **40**(6): p. 2647-2662.
146. Paul, A.S., et al., *Cysteine focused covalent inhibitors against the main protease of SARS-CoV-2.* Journal of Biomolecular Structure and Dynamics, 2022. **40**(4): p. 1639-1658.
147. Yepes-Pérez, A.F., O. Herrera-Calderon, and J. Quintero-Saumeth, *Uncaria tomentosa (cat's claw): a promising herbal medicine against SARS-CoV-2/ACE-2 junction and SARS-CoV-2 spike protein based on molecular modeling.* Journal of Biomolecular Structure and Dynamics, 2022. **40**(5): p. 2227-2243.
148. Shang, W., et al., *In vitro and in vivo evaluation of the main protease inhibitor FB2001 against SARS-CoV-2.* Antiviral Research, 2022. **208**: p. 105450.
149. Deodato, D., N. Asad, and T.M. Dore, *Discovery of 2-thiobenzimidazoles as noncovalent inhibitors of SARS-CoV-2 main protease.* Bioorganic & Medicinal Chemistry Letters, 2022. **72**: p. 128867.
150. Suryavanshi, H., et al., *Design, synthesis and docking study of Vortioxetine derivatives as a SARS-CoV-2 main protease inhibitor.* DARU Journal of Pharmaceutical Sciences, 2022. **30**(1): p. 139-152.
151. Londres, H.D., et al., *Blocking EGFR with nimotuzumab: a novel strategy for COVID-19 treatment.* Immunotherapy, 2022. **14**(7): p. 521-530.
152. Bharathi, M., et al., *In Silico Screening of Bioactive Compounds of Representative Seaweeds to Inhibit SARS-CoV-2 ACE2-Bound Omicron B.1.1.529 Spike Protein Trimer.* Marine Drugs, 2022. **20**(2): p. 148.
153. Nunes, V.S., et al., *Antivirals virtual screening to SARS-CoV-2 non-structural proteins.* Journal of Biomolecular Structure and Dynamics, 2022. **40**(19): p. 8989-9003.
154. Nag, A., et al., *An in-silico pharmacophore-based molecular docking study to evaluate the inhibitory potentials of novel fungal triterpenoid Astrakurkurone analogues against a hypothetical mutated main protease of SARS-CoV-2 virus.* Computers in Biology and Medicine, 2023. **152**: p. 106433.
155. Pérez-Vargas, J., et al., *Discovery of lead natural products for developing pan-SARS-CoV-2 therapeutics.* Antiviral Research, 2023. **209**: p. 105484.

156. Garrepalli, S., et al., *Synthesis and characterization of two known and one new impurities of dolutegravir: In silico evaluation of certain intermediates against SARS CoV-2 O-ribose methyltransferase (OMTase).* Journal of Molecular Structure, 2023. **1271**: p. 133992.
157. Singh, J.K., et al., *Neohesperidin and spike RBD interaction in omicron and its sub-variants: In silico, structural and simulation studies.* Computers in Biology and Medicine, 2023. **152**: p. 106392.
158. Rajaraman, D., et al., *One-pot synthesis, NMR, quantum chemical approach, molecular docking studies, drug-likeness and in-silico ADMET prediction of novel 1-(2,3-dihydrobenzo[b][1,4]dioxin-6-yl)-2-(furan-2-yl)-4,5-diphenyl-1H-imidazole derivatives.* Journal of Molecular Structure, 2023. **1273**: p. 134314.
159. kerkour, R., et al., *Novel α-aminophosphonate derivates synthesis, theoretical calculation, Molecular docking, and in silico prediction of potential inhibition of SARS-CoV-2.* Journal of Molecular Structure, 2023. **1272**: p. 134196.
160. Malebari, A.M., et al., *Exploring the dual effect of novel 1,4-diarylpyranopyrazoles as antiviral and anti-inflammatory for the management of SARS-CoV-2 and associated inflammatory symptoms.* Bioorganic Chemistry, 2023. **130**: p. 106255.
161. Abdel-Mohsen, H.T., et al., *Discovery of novel thioquinazoline-N-aryl-acetamide/N-arylacetohydrazide hybrids as anti-SARS-CoV-2 agents: Synthesis, in vitro biological evaluation, and molecular docking studies.* Journal of Molecular Structure, 2023. **1276**: p. 134690.
162. Wang, R., et al., *Discovery and mechanism of action of Thonzonium bromide from an FDA-approved drug library with potent and broad-spectrum inhibitory activity against main proteases of human coronaviruses.* Bioorganic Chemistry, 2023. **130**: p. 106264.
163. Parthasarathy, H., et al., *Metformin suppresses SARS-CoV-2 in cell culture.* Virus Research, 2023. **323**: p. 199010.
164. Gurung, A.B., et al., *Masitinib analogues with the N-methylpiperazine group replaced – A new hope for the development of anti-COVID-19 drugs.* Journal of King Saud University - Science, 2023. **35**(1): p. 102397.
165. Sasaki, M., et al., *S-217622, a SARS-CoV-2 main protease inhibitor, decreases viral load and ameliorates COVID-19 severity in hamsters.* Sci Transl Med, 2023. **15**(679): p. eabq4064.
166. Gao, H., R. Dai, and R. Su, *Computer-aided drug design for the pain-like protease (PLpro) inhibitors against SARS-CoV-2.* Biomedicine & Pharmacotherapy, 2023. **159**: p. 114247.
167. Tan, B., et al., *SARS-CoV-2 Main Protease Drug Design, Assay Development, and Drug Resistance Studies.* Acc Chem Res, 2023. **56**(2): p. 157-168.
168. Ma, L., et al., *Teicoplanin derivatives block spike protein mediated viral entry as pan-SARS-CoV-2 inhibitors.* Biomedicine & Pharmacotherapy, 2023. **158**: p. 114213.
169. Abreu Alves, P., et al., *Withanolides of Athenaea velutina with potential inhibitory properties against SARS coronavirus main protease (mpro): molecular modeling studies.* Journal of Biomolecular Structure and Dynamics, 2023: p. 1-9.
170. Malik, A., et al., *In silico screening of phytochemical compounds and FDA drugs as potential inhibitors for NSP16/10 5' methyl transferase activity.* Journal of Biomolecular Structure and Dynamics, 2023. **41**(1): p. 221-233.
171. Alamshany, Z.M., et al., *Synthesis and Molecular Docking Study of Novel Pyrimidine Derivatives against COVID-19.* Molecules, 2023. **28**(2).
172. Liu, M., et al., *Discovery and structural optimization of 3-O-β-Chacotriosyl betulonic acid saponins as potent fusion inhibitors of Omicron virus infections.* Bioorg Chem, 2023. **131**: p. 106316.

173. Magurano, F., et al., *A potential host and virus targeting tool against COVID-19: Chemical characterization, antiviral, cytoprotective, antioxidant, respiratory smooth muscle relaxant effects of Paulownia tomentosa Steud.* Biomed Pharmacother, 2023. **158**: p. 114083.
174. Arya, R., V. Prashar, and M. Kumar, *Identification and characterization of aurintricarboxylic acid as a potential inhibitor of SARS-CoV-2 PLpro.* Int J Biol Macromol, 2023: p. 123347.
175. Sabzian-Molaei, F., et al., *Urtica dioica agglutinin (UDA) as a potential candidate for inhibition of SARS-CoV-2 Omicron variants: In silico prediction and experimental validation.* Phytomedicine, 2023. **111**: p. 154648.
176. Khamto, N., et al., *Discovery of Natural Bisbenzylisoquinoline Analogs from the Library of Thai Traditional Plants as SARS-CoV-2 3CL(Pro) Inhibitors: In Silico Molecular Docking, Molecular Dynamics, and In Vitro Enzymatic Activity.* J Chem Inf Model, 2023.
177. de Araujo, I.G., et al., *Potential of plant extracts in targeting SARS-CoV-2 main protease: an in vitro and in silico study.* J Biomol Struct Dyn, 2023: p. 1-10.
178. Lingwan, M., et al., *Phytochemical rich Himalayan Rhododendron arboreum petals inhibit SARS-CoV-2 infection in vitro.* Journal of Biomolecular Structure and Dynamics, 2023. **41**(4): p. 1403-1413.
179. Trimarco, V., et al., *Beneficial effects of L-Arginine in patients hospitalized for COVID-19: New insights from a randomized clinical trial.* Pharmacological Research, 2023. **191**: p. 106702.
180. Terada, J., et al., *Favipiravir, camostat, and ciclesonide combination therapy in patients with moderate COVID-19 pneumonia with/without oxygen therapy: An open-label, single-center phase 3 randomized clinical trial.* eClinicalMedicine, 2022. **49**: p. 101484.
181. Khorshiddoust, R.R., et al., *Efficacy of a multiple-indication antiviral herbal drug (Saliravira®) for COVID-19 outpatients: A pre-clinical and randomized clinical trial study.* Biomedicine & Pharmacotherapy, 2022. **149**: p. 112729.
182. Dastenae, Z.H., et al., *Comparison of the effect of intravenous dexamethasone and methylprednisolone on the treatment of hospitalized patients with COVID-19: a randomized clinical trial.* International Journal of Infectious Diseases, 2022. **122**: p. 659-664.
183. Farnoosh, G., et al., *Efficacy of a Low Dose of Melatonin as an Adjunctive Therapy in Hospitalized Patients with COVID-19: A Randomized, Double-blind Clinical Trial.* Archives of Medical Research, 2022. **53**(1): p. 79-85.
184. Rossignol, J.-F., et al., *A randomized double-blind placebo-controlled clinical trial of nitazoxanide for treatment of mild or moderate COVID-19.* eClinicalMedicine, 2022. **45**: p. 101310.
185. Koshak, A.E., et al., *Nigella sativa for the treatment of COVID-19: An open-label randomized controlled clinical trial.* Complementary Therapies in Medicine, 2021. **61**: p. 102769.
186. Reis, G., et al., *Effect of early treatment with fluvoxamine on risk of emergency care and hospitalisation among patients with COVID-19: the TOGETHER randomised, platform clinical trial.* The Lancet Global Health, 2022. **10**(1): p. e42-e51.
187. Devpura, G., et al., *Randomized placebo-controlled pilot clinical trial on the efficacy of ayurvedic treatment regime on COVID-19 positive patients.* Phytomedicine, 2021. **84**: p. 153494.
188. Udwadia, Z.F., et al., *Efficacy and safety of favipiravir, an oral RNA-dependent RNA polymerase inhibitor, in mild-to-moderate COVID-19: A randomized, comparative, open-label, multicenter, phase 3 clinical trial.* International Journal of Infectious Diseases, 2021. **103**: p. 62-71.
189. Silveira, M.A.D., et al., *Efficacy of Brazilian green propolis (EPP-AF®) as an adjunct treatment for hospitalized COVID-19 patients: A randomized, controlled clinical trial.* Biomedicine & Pharmacotherapy, 2021. **138**: p. 111526.
190. Duarte, M., et al., *Telmisartan for treatment of Covid-19 patients: An open multicenter randomized clinical trial.* eClinicalMedicine, 2021. **37**: p. 100962.

191. Xiong, W.-z., et al., *Efficacy of herbal medicine (Xuanfei Baidu decoction) combined with conventional drug in treating COVID-19:A pilot randomized clinical trial.* Integrative Medicine Research, 2020. **9**(3): p. 100489.
192. Dastan, F., et al., *Promising effects of tocilizumab in COVID-19: A non-controlled, prospective clinical trial.* International Immunopharmacology, 2020. **88**: p. 106869.
193. Zhou, S., et al., *Traditional Chinese medicine shenhuang granule in patients with severe/critical COVID-19: A randomized controlled multicenter trial.* Phytomedicine, 2021. **89**: p. 153612.
194. Montgomery, H., et al., *Efficacy and safety of intramuscular administration of tixagevimab–cilgavimab for early outpatient treatment of COVID-19 (TACKLE): a phase 3, randomised, double-blind, placebo-controlled trial.* The Lancet Respiratory Medicine, 2022. **10**(10): p. 985-996.
195. Nicastri, E., et al., *A phase 2 randomized, double-blinded, placebo-controlled, multicenter trial evaluating the efficacy and safety of raloxifene for patients with mild to moderate COVID-19.* eClinicalMedicine, 2022. **48**: p. 101450.
196. Sholzberg, M., et al., *Randomized trials of therapeutic heparin for COVID-19: A meta-analysis.* Research and Practice in Thrombosis and Haemostasis, 2021. **5**(8): p. e12638.
197. Biber, A., et al., *The effect of ivermectin on the viral load and culture viability in early treatment of nonhospitalized patients with mild COVID-19 – a double-blind, randomized placebo-controlled trial.* International Journal of Infectious Diseases, 2022. **122**: p. 733-740.
198. Al-Abdouh, A., et al., *Remdesivir for the treatment of COVID-19: A systematic review and meta-analysis of randomized controlled trials.* Contemporary Clinical Trials, 2021. **101**: p. 106272.
199. Yu, B. and J. Chang, *Azvudine (FNC): a promising clinical candidate for COVID-19 treatment.* Signal Transduction and Targeted Therapy, 2020. **5**(1): p. 236.
