# Supplementary material for: Comprehensive review on COVID-19: etiology, pathogenicity, and treatment
Source: Front Med (Lausanne). 2025 Sep 23;12:1569013. doi: 10.3389/fmed.2025.1569013 (PMC12502089; doi:10.3389/fmed.2025.1569013)
Supplement: Supplementary file 2 [file Data_Sheet_2.pdf]

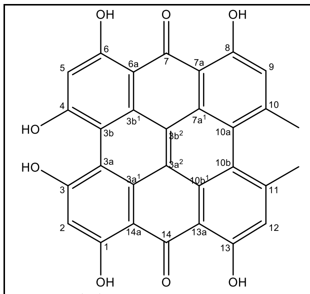 Hypericin	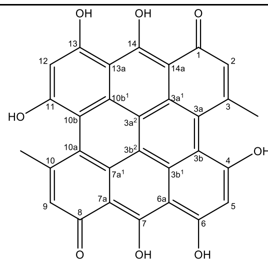 Isohypericin	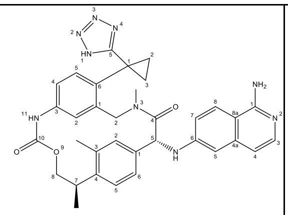 (2r,15r)-2-[(1-aminoisoquinolin-6-Yl)amino]-4,15,17-trimethyl-7-[1-(1h-tetrazol-5-Yl)cyclopropyl]-13-Oxa-4,11-diazatricyclo [14.2.2.1~6,10~]henicosa-1(18),6(21),7,9,16,19-hexaene-3,12-dione	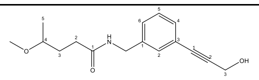 N-[[3-(3-hydroxyprop-1-ynyl)phenyl]methyl]-4-methoxypentanamide
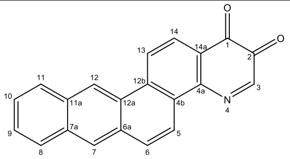 Anthrachinolinchinon	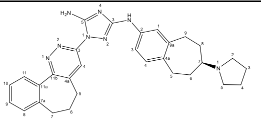 Bemcentinib	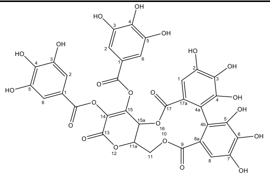 Emblicanin A	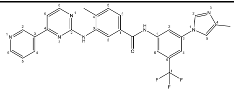 Nilotinib
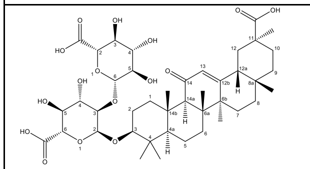 Glycyrrhizin	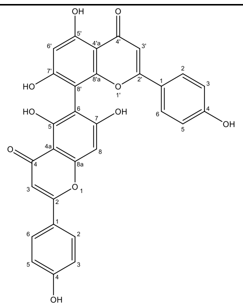 Agathisflavone	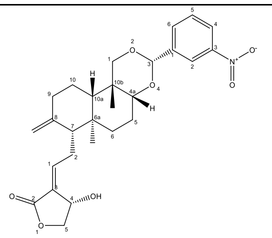 (S,E)-3-(2-((3S,4aR,6aS,7R,10aS,10bR)-6a,10b-dimethyl-8-methylene-3-(3-nitrophenyl)decahydro-1H-naphtho[2,1-d][1,3]dioxin-7-yl)ethylidene)-4-hydroxydihydrofuran-2(3H)-one	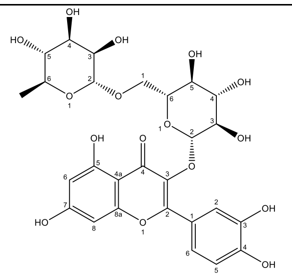 Rutin
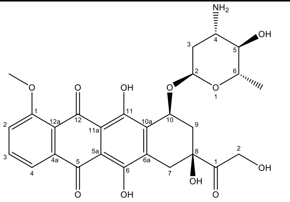 Epirubicin	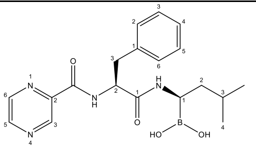 Bortezomib	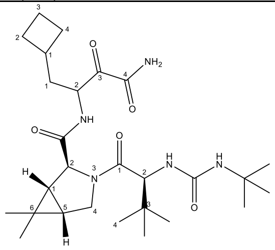 Boceprevir	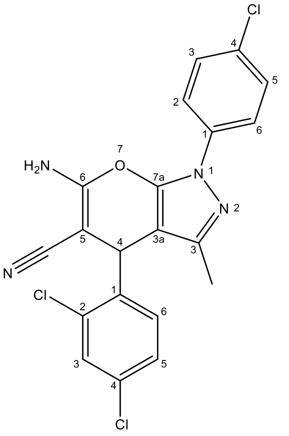 6-Amino-1-(4-chlorophenyl)-4-(2,4-dichlorophenyl)-3-methyl-1,4-

			dihydropyrano[2,3-c]pyrazole-5-carbonitrile
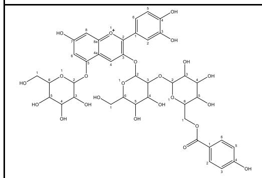 [6-[2-[2-(3,4-dihydroxyphenyl)-7-hydroxy-5-[3,4,5-trihydroxy-6-(hydroxymethyl)oxan-2-yl]oxychromenylium-3-yl]oxy-4,5-dihydroxy-6-(hydroxymethyl)oxan-3-yl]oxy-3,4,5-trihydroxyoxan-2-yl]methyl 4-hydroxybenzoate (Compound ID 131831710)	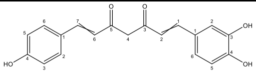 1-(4-hydroxyphenyl)-7-(3,4-dihydroxyphenyl)-1 6-heptadiene-3,5-dione	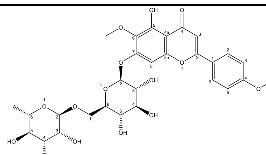 Pectolinarin	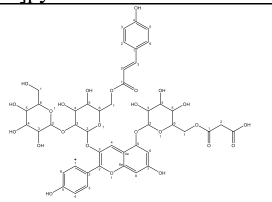 3-[[6-[3-[4,5-dihydroxy-6-[[(*E*)-3-(4-hydroxyphenyl)prop-2-enoyl]oxymethyl]-3-[3,4,5-trihydroxy-6-(hydroxymethyl)oxan-2-yl]oxyoxan-2-yl]oxy-7-hydroxy-2-(4-hydroxyphenyl)chromenylium-5-yl]oxy-3,4,5-trihydroxyoxan-2-yl]methoxy]-3-oxopropanoic acid (Compound ID 131751762)
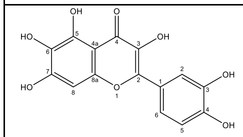 Quercetagetin	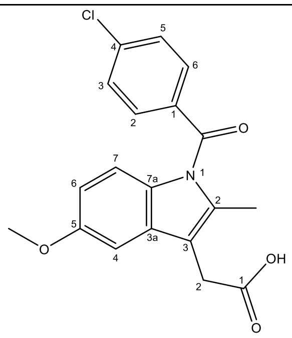 Indomethacin	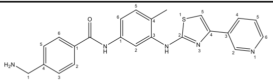 4-(aminomethyl)-N-[4-methyl-3-[(4-pyridin-3-yl-1,3-thiazol-2-yl)amino]phenyl]benzamide	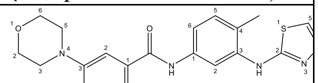 N-[4-methyl-3-[(4-pyridin-3-yl-1,3-thiazol-2-yl)amino]phenyl]-3-morpholin-4-ylbenzamide
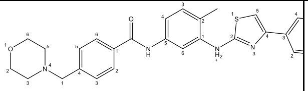 [2-methyl-5-[[4-(morpholin-4-ylmethyl)benzoyl]amino]phenyl]-(4-pyridin-3-yl-1,3-thiazol-2-yl)azanium **(CID143003625)**	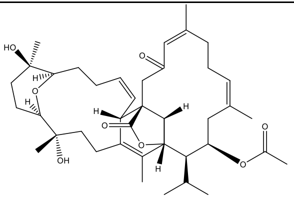 Bislatumlide A	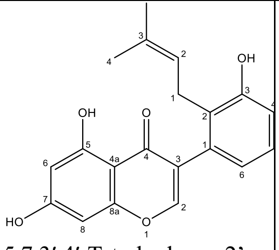 5,7,3′,4′-Tetrahydroxy-2’-(3,3-dimethylallyl) isoflavone	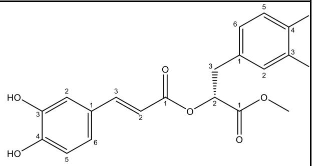 Methyl rosmarinate
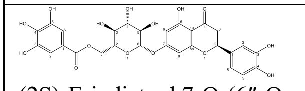 (2S)-Eriodictyol 7-O-(6″-O-galloyl)-beta-D-glucopyranoside	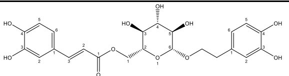 Calceolarioside B	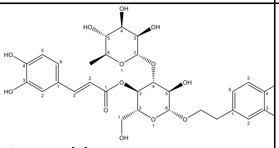 Acetoside	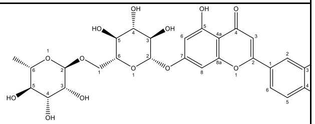 Luteolin 7-rutinoside

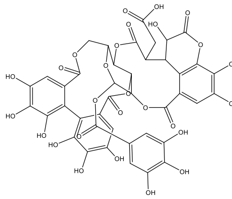 Chebulagic acid	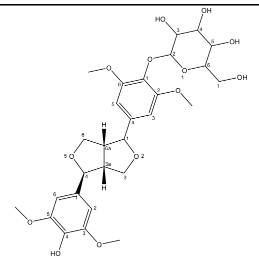 Syrigaresinol	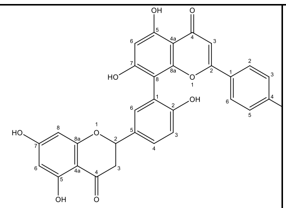 2,3-Dihydroamentoflavone	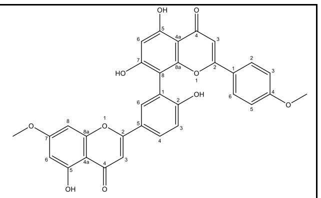 Podocarpusflavon-B
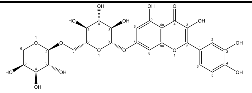 Quercimeritrin 6''-O-L-arabinopyranoside	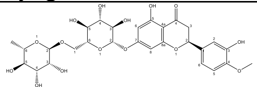 Hesperidin	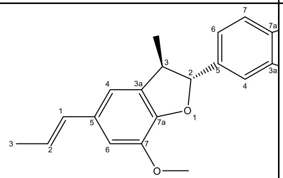 Licarin B	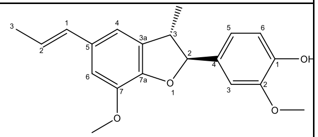 Licarin A
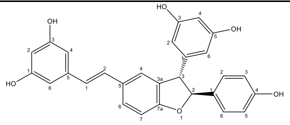 Delta-viniferin	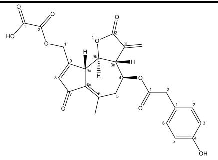 Lactucopicrin 15	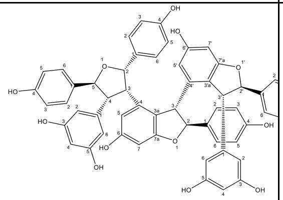 Kobophenol A	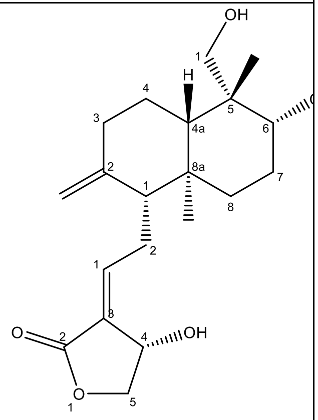 Andrographolide
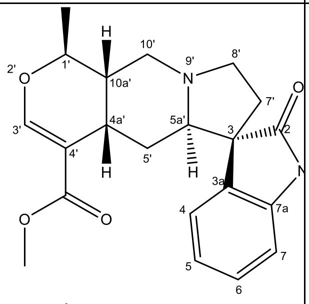 Uncarine F	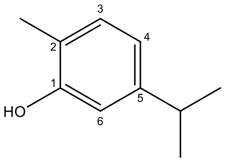 Isothymol	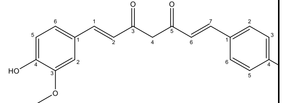 Demethoxycurcumin	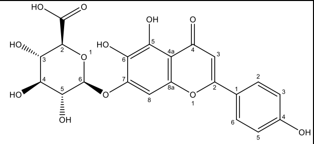 Scutellarin
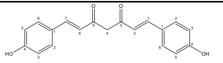 Bisdemethoxycurcumin	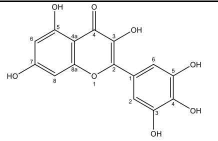 Myricetin	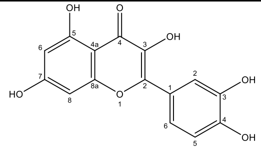 Quercetin	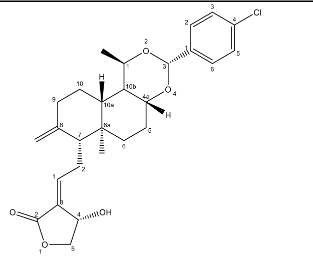 (4S,E)-3-(2-((1R,3S,4aR,6aR,7R,10aR)-3-(4-chlorophenyl)-1,6a-dimethyl-8-methylenedecahydro-1H-naphtho[2,1-d][1,3]dioxin-7-yl)ethylidene)-4-

			hydroxydihydrofuran-2(3H)-one
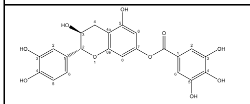<br>Catechin-7-o-gallate	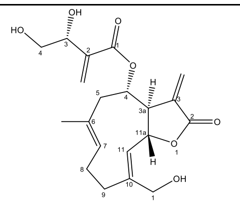<br>Cnicin	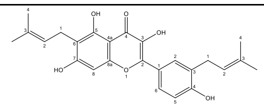<br>Glyasperin A	
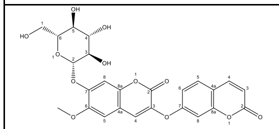<br>Daphnorin	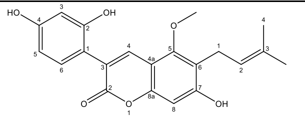<br>Glycycoumarin	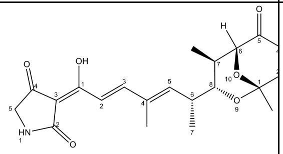<br>Tirandamycin A	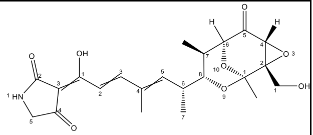<br>Isotirandamycin B
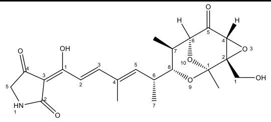<br>Tirandamycin B	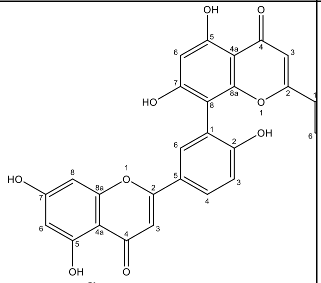<br>Amentoflavone	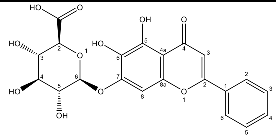<br>Baicalin	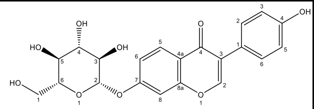<br>Daidzin
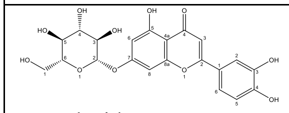<br>Luteoloside	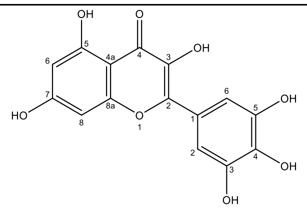<br>Myricetin	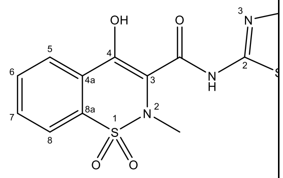<br>Meloxicam	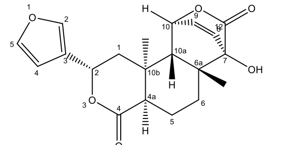<br>Columbin
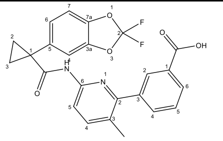<br>Lumacaftor	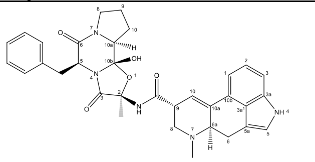<br>Ergotamine	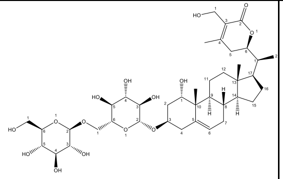<br>Withanoside-IV	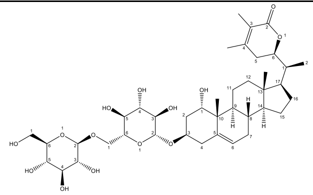<br>Withanoside V
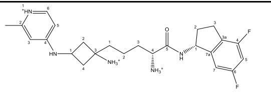<br>4-((3-ammonio-3-((R)-4-ammonio-5-(((R)-4,6-difluoro-2,3-dihydro-1H-inden-1-yl)amino)-5-oxopentyl)cyclobutyl)amino)-2-methylpyridin-1-ium	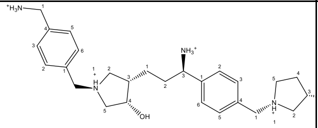<br>(1R,3S,4S)-3-((R)-3-ammonio-3-(4-(((1S,3S)-3-hydroxypyrrolidin-1-ium-1-yl)methyl)phenyl)propyl)-1-(4-(ammoniomethyl)benzyl)-4-hydroxypyrrolidin-1-ium	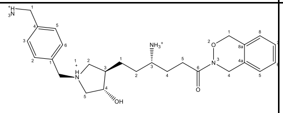<br>(1R,3R,4S)-3-((S)-3-ammonio-6-(1,4-dihydro-3H-benzo[d][1,2]oxazin-3-yl)-6-oxohexyl)-1-(4-(ammoniomethyl)benzyl)-4-hydroxypyrrolidin-1-ium	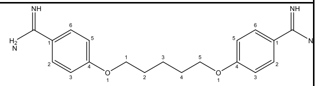<br>Pentamidine

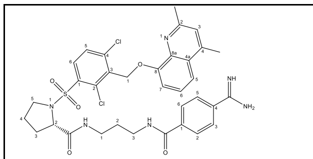<br>Anatibant	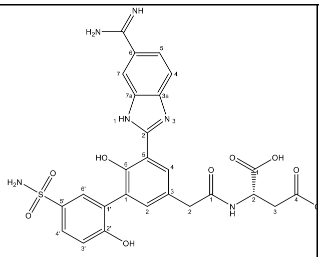<br>PCI-27483	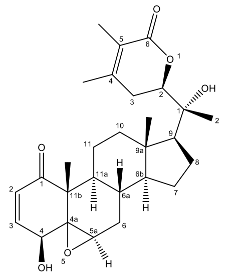<br>Withanolide	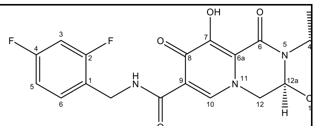<br>Dolutegravir
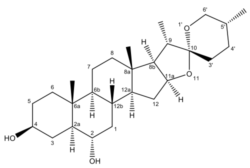<br>Chlorogenin	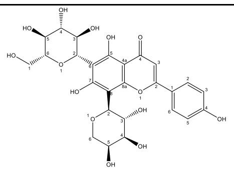<br>Schaftoside	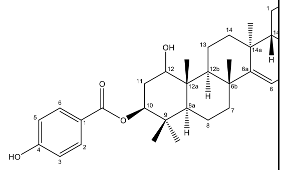<br>1-Hydroxyaleuritolic acid 3-p-hydroxybenzoate	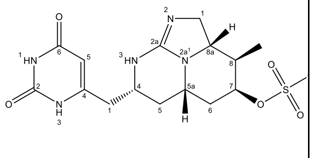<br>Deoxycylindrospermopsin
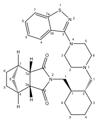<br>Lurasidone	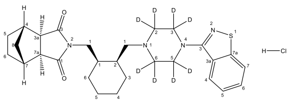<br>Lurasidone exo	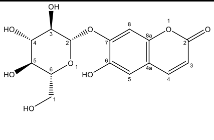<br>Cichoriin	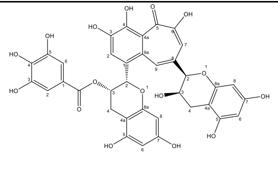<br>Theaflavin 3-gallate
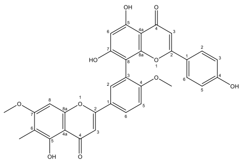<br>Taiwanhomoflavone A	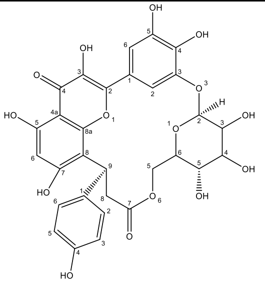<br>Nympholide A	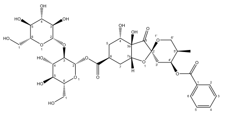<br>Phyllaemblicin B	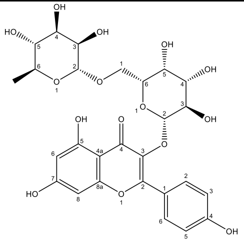<br>Biorobin
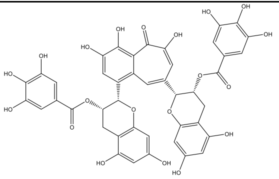<br>Theaflavin 3,3'digallate	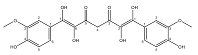<br>Tetrahydroxycurcumin	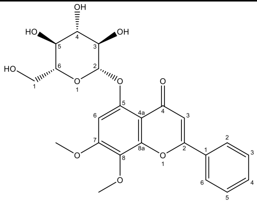<br>Andrographidine C	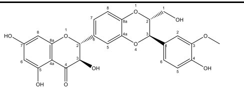<br>Silibinin
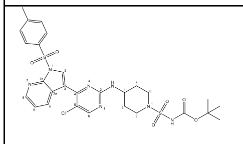<br>Tert-butyl N-[4-[[5-chloro-4-[1-(4-methylphenyl)sulfonylpyrrolo[2,3-b]pyridin-3-yl]pyrimidin-2-	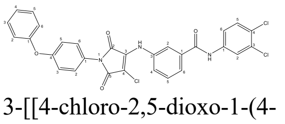<br>3-[[4-chloro-2,5-dioxo-1-(4-phenoxyphenyl)pyrrol-3-yl]amino]-N-(3,4-dichlorophenyl)benzamide	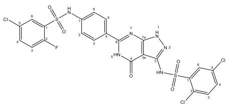<br>2,5-dichloro-N-(6-(4-((5-chloro-2-fluorophenyl)sulfonamido)phenyl)-4-oxo-4,5-dihydro-1H-pyrazolo[3,4-	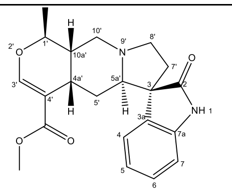<br>Speciophylline

yl]amino]piperidin-1-yl]sulfonylcarbamate		d]pyrimidin-3-yl)benzenesulfonamide	
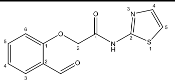<br>2-(2-formylphenoxy)-N-(thiazol-2-yl)acetamide	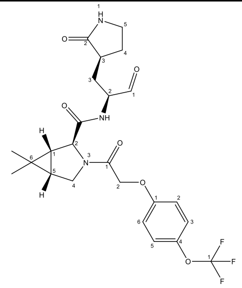<br>(1R,2S,5S)-6,6-dimethyl-N-((S)-1-oxo-3-((S)-2-oxopyrrolidin-3-yl)propan-2-yl)-3-(2-(4- (trifluoromethoxy) phenoxy) acetyl)-3-azabicyclo[3.1.0]hexane-2-carboxamide	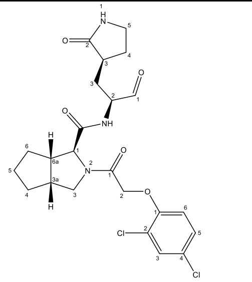<br>(1S,3aR,6aS)-2-(2-(2,4-dichlorophenoxy)acetyl)-N-((S)-1-oxo-3-((S)-2-oxopyrrolidin-3-yl)propan-2-yl)octahydrocyclopenta[c]pyrrole-1-carboxamide	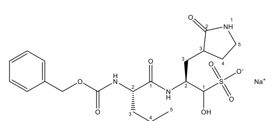<br>GC376
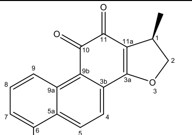<br>Dihydrotanshinone I	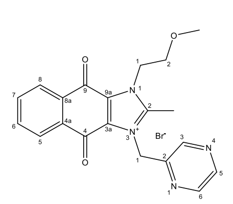<br>Sepantronium bromide	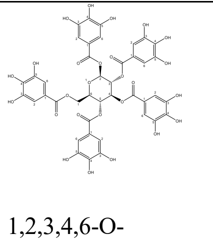<br>1,2,3,4,6-O-Pentagalloylglucose	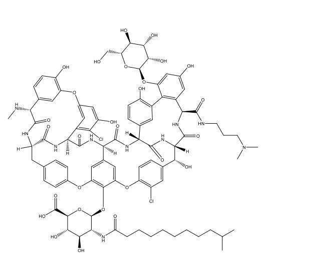<br>Dalbavancin
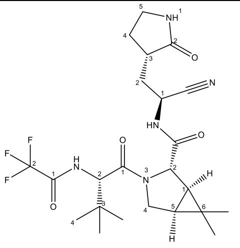<br>Nirmatrelvir	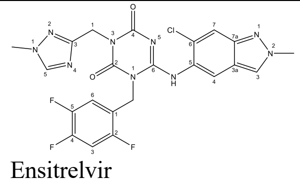<br>Ensitrelvir	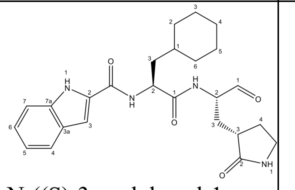<br>N-((S)-3-cyclohexyl-1-oxo-1-(((S)-1-oxo-3-((S)-2-oxopyrrolidin-3-yl)propan-2-yl)amino)propan-2-yl)-1H-indole-2-carboxamide (FB2001)	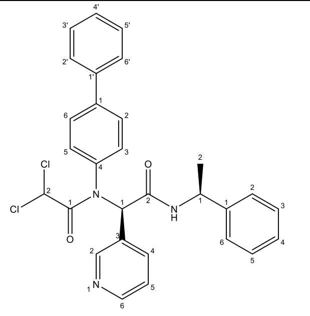<br>N-([1,1'-biphenyl]-4-yl)-2,2-dichloro-N-((R)-2-oxo-2-(((S)-1-phenylethyl)amino)-1-(pyridin-3-yl)ethyl)acetamide
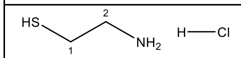<br>Cysteamine HCl	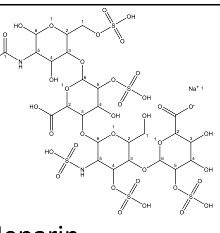<br>Heparin	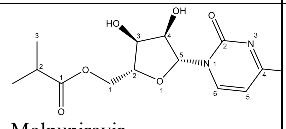<br>Molnupiravir	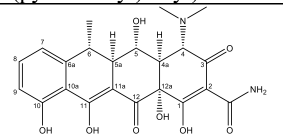<br>Doxycycline

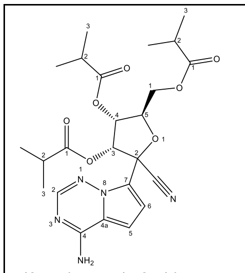<br>(3R,4R,5R)-2-(4-aminopyrrolo[2,1-f][1,2,4]triazin-7-yl)-2-cyano-5-((isobutyryloxy)methyl)tetrahydrofuran-3,4-diyl bis(2-methylpropanoate**)**	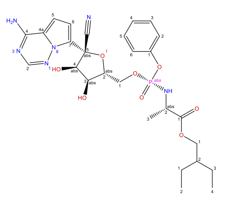<br>Remdesivir	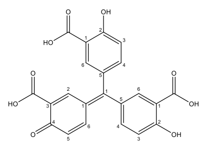<br>Aurintricarboxylic acid	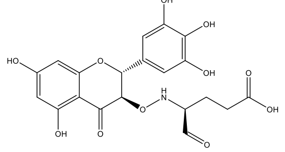<br>,5,7,3′,4′,5′-hexahydroxy flavanone -3-O-beta-D-glucopyranoside
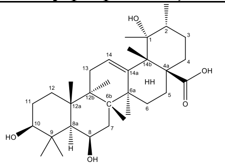<br>Uncaric acid	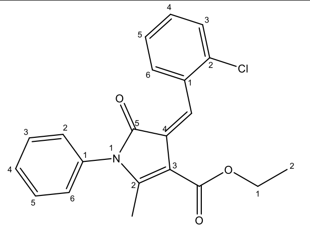<br>N′,N″-[oxybis(4,1-phenylenecarbonyl)]bis(3-methoxybenzohydrazide) (7781334)	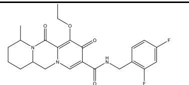<br>(4S,12 aR)-N-(2,4-difluorobenzyl)-7-ethoxy-4- methyl-6,8-dioxo-3,4,6,8,12,12 a -hexahydro-2 H -pyrido[1´,2´:4,5]pyrazino[2,1- b][1,3]oxazine-9-carboxamide	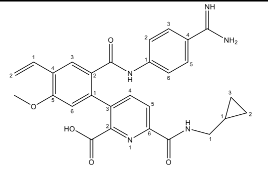<br>Avoralstat
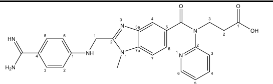<br>Dabigatran	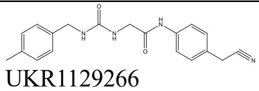<br>UKR1129266	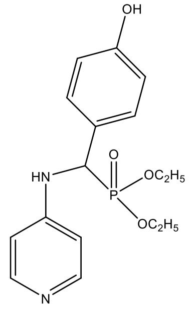<br>Diethyl(4-hydroxyphenyl)[(pyridine-4-yl)amino]methylphosphonate	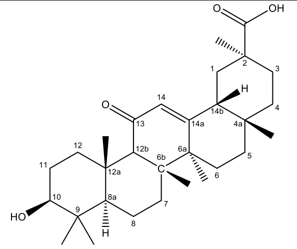<br>Glycyrrhetinic acid
